# A statistical perspective on baseline adjustment in pharmacogenomic genome-wide association studies of quantitative change

**DOI:** 10.1038/s41525-022-00303-2

**Published:** 2022-06-09

**Authors:** Hong Zhang, Aparna Chhibber, Peter M. Shaw, Devan V. Mehrotra, Judong Shen

**Affiliations:** 1 Biostatistics and Research Decision Sciences, Merck & Co., Inc., Rahway, NJ 07065 USA; 2 Genetics and Biomarker Sciences, Merck & Co., Inc, West Point, PA 19446 USA; 3 Biostatistics and Research Decision Sciences, Merck & Co., Inc, North Wales, PA 19454 USA; 4 Present Address: Bristol Myers Squibb, Lawrenceville, NJ 08540 USA

**Keywords:** Pharmaceutics, Pharmacogenomics

## Abstract

In pharmacogenetic (PGx) studies, drug response phenotypes are often measured in the form of change in a quantitative trait before and after treatment. There is some debate in recent literature regarding baseline adjustment, or inclusion of pre-treatment or baseline value as a covariate, in PGx genome-wide association studies (GWAS) analysis. Here, we provide a clear statistical perspective on this baseline adjustment issue by running extensive simulations based on nine statistical models to evaluate the influence of baseline adjustment on type I error and power. We then apply these nine models to analyzing the change in low-density lipoprotein cholesterol (LDL-C) levels with ezetimibe + simvastatin combination therapy compared with simvastatin monotherapy therapy in the 5661 participants of the IMPROVE-IT (IMProved Reduction of Outcomes: Vytroin Efficacy International Trial) PGx GWAS, supporting the conclusions drawn from our simulations. Both simulations and GWAS analyses consistently show that baseline-unadjusted models inflate type I error for the variants associated with baseline value if the baseline value is also associated with change from baseline (e.g., when baseline value is a mediator between a variant and change from baseline), while baseline-adjusted models can control type I error in various scenarios. We thus recommend performing baseline-adjusted analyses in PGx GWASs of quantitative change.

## Introduction

Pharmacogenetic studies (PGx) aim to identify genetic biomarkers associated with efficacy and safety of drugs^1^. Drug response phenotypes are often measured in the form of change from pre-treatment (baseline) values of a quantitative trait. The goal of this type of analysis is to identify genetic effects on drug related changes independent of the baseline values. For many quantitative measures used in drug response analyses, the magnitude of change in a score with treatment is correlated with the pre-treatment value. This correlation may be driven by true associations between these values or may be introduced through intra-subject variability, errors in the quantitative measurements used, or because of the regression-to-the-mean phenomenon, a phenomenon in which repeated measures of a value will tend to come closer to the mean of the group from which they are sampled^2^. Because genetic variants can be associated with the baseline values of a trait, care must be taken in adequately controlling for any correlation between pre-treatment and post-treatment values. However, there is considerable debate in the literature on the best approach to preventing false associations between a variable of interest (such as genotype) and the quantitative change driven by associations with the baseline values^3–5^. Specifically, in the context of PGx studies, a recent publication explores the impact of adjusting for baseline by conducting a series of GWASs for response to statin therapy as measured by change in low-density lipoprotein cholesterol (LDL-C) and supports the approach for not adjusting baseline values in GWAS analysis of quantitative change from baseline (CFB)^6^.

In this paper, we use systematic simulations and rigorous statistical modeling to explore this issue and make recommendations on the best analysis approach. We have studied the relationship among different statistical models, including baseline-adjusted and unadjusted models. More specifically, we have conducted extensive simulations to compare the type I error and power performance of nine statistical models (Methods) and demonstrate that: (1) baseline-unadjusted models tend to generate inflated type I errors when the baseline value is a mediator, i.e., when the baseline value is associated with the genotype and meanwhile associated with CFB or explains at least some of the association between genotype and CFB, while baseline-adjusted models can control type I error in all simulated scenarios; (2) the power of the baseline-adjusted and unadjusted models are similar when there is no mediator effect; and (3) the power of the baseline-adjusted model is higher than the unadjusted model when the genetic effect is in a different direction from the mediator effect. Based on the simulation results, we further argue that, although measurement errors could inflate the type I error of the baseline-adjusted models, not adjusting the baseline is also likely to cause type I error inflation due to the potential mediator effect of the baseline. We thus recommend performing baseline-adjusted analyses as the primary analysis. If type I error rate from baseline-adjusted analyses is still inflated which may be caused by measurement error, baseline-unadjusted analyses may be performed for further diagnosis. The main points from our simulations are further supported by the analyses of change in LDL-C using IMPROVE-IT PGx data based on nine statistical models (Methods).

## Results

Nine statistical models were evaluated in both simulations and in the analysis of a real PGx GWAS dataset. These include models that measure change as both the transformed residuals of log-fold-CFB and the CFB ratio. The log-fold change from the baseline endpoints was also analyzed directly without transforming residuals. In addition, a two degree of freedom (2df) test that incorporates a treatment by genotype interaction term was also tested. All models were evaluated both with and without adjusting for natural log-transformed baseline values.

### Simulation results: type I error

**Fig. 1:**
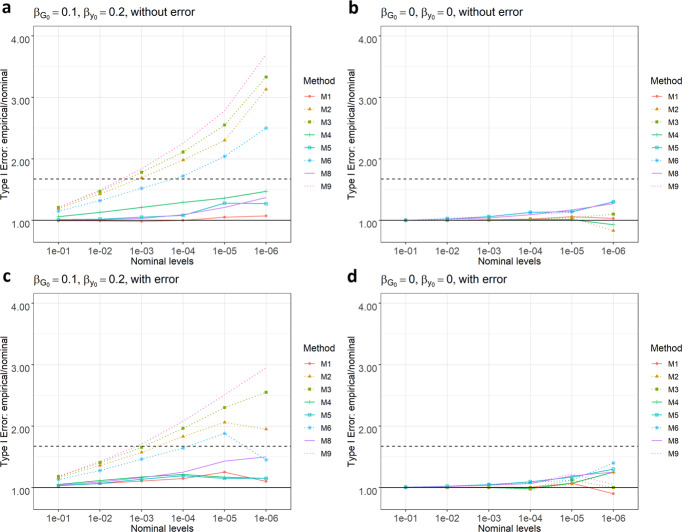
Ratios between empirical type I error rates and the nominal α levels. Upper panels (**a** and **b**): no measurement errors. Lower panels (**c** and **d**): white noise measurement error (normal relative error rate with mean zero). Dotted curves: baseline-unadjusted models. Horizontal dash line: the ratio ($\alpha$ + 3*SE)/$\alpha$ = 1 + 3*SE/$\alpha \approx 1.67$, where SE is the margin of error calculated as $\sqrt {\frac{{\alpha \ast (1 - \alpha )}}{n}}$, $\alpha = 10^{ - 6}$ is the nominal level and $n = 2 \times 10^7$ is the number of simulations. M1–M9 are defined in the “Methods” section.

The type I error simulation results based on various simulation settings (Methods) are summarized in Fig. 1. When the mediator effect $\beta _{G_0} \ast \beta _{y_0} \,\ne\, 0$ (i.e., when there is both an association between genotype and baseline value or $\beta _{G_0} \,\ne\, 0$ and an association between baseline value and CFB or $\beta _{y_0} \,\ne\, 0$ in Fig. 1a, c), the baseline-unadjusted models (dotted curves) can inflate the type I errors as high as $3.5 \times \alpha$, where $\alpha$ is the nominal type I error rate, $\beta _{G_0}$ is the effect size of the association between genotype and baseline and $\beta _{y_0}$ measures the dependence of CFB on the baseline. The baseline-adjusted models (solid curves), however, can control the type I errors at the nominal levels. This is true regardless of the transformations applied (log vs. percentage), statistical tests (1df vs. 2df), regression approaches (1-step vs. 2-step) and measurement errors (no error vs. white noises). More variation observed at the smaller $\alpha$ levels is due to Monte-Carlo errors, i.e., simulation-driven variability. We further explored different settings of $\beta _{G_0}$ and $\beta _{y_0}$. As evidenced in Supplementary Figs. 1 and 2, the results are consistent with different combinations of $\beta _{G_0}$ and $\beta _{y_0}$. If their product is not zero, the baseline-unadjusted models will inflate the type I errors substantially in both the presence and absence of measurement error for almost all $\alpha$ (except for very large $\alpha$). Clifton and Clifton derived the correlation between the baseline score and CFB score and showed that there is always a correlation (usually negative, also see an example from our IMPROVE-IT data in Supplementary Fig. 3) between the change score and baseline score^7^, which means $\beta _{y_0}$ is usually a negative value. Thus, the mediator effect usually depends on $\beta _{G_0}$, the baseline genetic effect size.

The impacts of different measurement errors on the type I errors were also examined. As shown in Supplementary Fig. 4, we find that the type I errors of baseline-adjusted models are inflated only if the errors of baseline and post-treatment measurements are in different direction (configuration iii). In contrast, if the measurement errors are both white noises (configuration i), or if they are in the same direction (configuration ii), the type I errors of baseline-adjusted models are well-controlled.

In the sensitivity analysis of modeling post-treatment values, Supplementary Fig. 5 shows that the type I errors of such models (M5* and M6*) follow the same pattern with CFB models (M5 and M6). That is, if the mediator effect is non-zero, then the baseline-unadjusted models will have inflated type I errors. The baseline-adjusted models again can control type I error well except for measurement error configuration iii.

### Simulation results: power

**Fig. 2:**
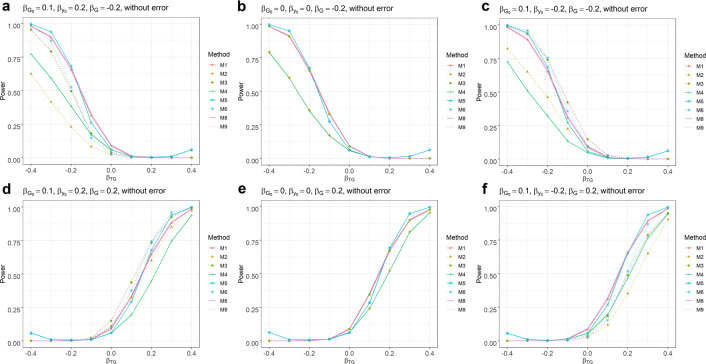
Power comparison between baseline-adjusted and unadjusted models. Upper panels (**a**–**c**): $\beta _G = - 0.2$. Lower panels (**d**–**f**): $\beta _G = 0.2$. First, second, third column: $\left( {\beta _{G_0},\beta _{y_0}} \right) = \left( {0.1,0.2} \right),\left( {0,0} \right),(0.1, - 0.2)$, respectively. $\alpha = 10^{ - 6}$, which is consistent with the type I error simulation. M1–M9 are defined in the “Methods” section.

The power simulation results based on various simulation settings (Methods) are summarized in Fig. 2. Note that the power presented is not adjusted for potentially inflated type I error rates for the baseline-unadjusted models. The power of the baseline-adjusted models is higher than the corresponding baseline-unadjusted models under the condition that the mediator effect $\beta _{G_0} \ast \beta _{y_0}$ is in a different direction from the genetic effects $\beta _G$. For example, given baseline is often negatively associated with CFB^7^, the condition is met when baseline genetic effect $\beta _{G_0}$ is in the same direction of the genetic effects $\beta _G$, that is, when an allele is associated with both higher baseline value and larger CFB or when an allele is associated with both lower baseline value and smaller CFB. The baseline-unadjusted models have higher power when baseline genetic effect $\beta _{G_0}$ is in the opposite direction of the genetic effects $\beta _G$ given baseline is negatively associated with CFB. However, note that such power advantage of the baseline-unadjusted model comes (at least partially) at the cost of higher type I error described above. If there is no mediator effect (Fig. 2b, e), then the baseline adjustment will not affect power. This observation sheds light on understanding the different *p* values generated by the baseline-adjusted or unadjusted models for the top signals in the IMPROVE-IT GWAS data analysis. We will discuss the power comparison more in the “IMPROVE-IT GWAS analysis results from nine statistical models” section.

Another observation worth mentioning is that when the genetic interaction effect $\beta _{GT}$ is larger, the power of the 2df test (model M5 or M6) is higher than the power of a corresponding 1df test (model M1 or M3). This is not surprising since we explicitly specify an interaction term in the 2df model (M5) to model the interaction effect. This term will capture the interaction effect if it is present. However, when there isn’t any interaction effect, the interaction term will be pure noise thus adding it in the model reduces the power.

More power simulation results are shown in Supplementary Figs. 6–8 across different combinations of genetic effects $(\beta _G,\beta _{GT})$ and parameters $(\beta _{G_0},\beta _{y_0})$. The observation is consistent: if the genetic effects are in different direction from the mediator effect $( {\beta _{G_0} \ast \beta _{y_0}} )$, then the power of the baseline-adjusted model is higher; otherwise, the unadjusted models have higher power (prior to adjusting for type I error inflation).

We also compare the power of post-treatment value models (M5*, M6* and *Q* test) with the CFB models M5 and M6. Supplementary Fig. 9 shows that the baseline-adjusted model M5* has comparable power with M5. The power of *Q* test is the lowest among all methods.

### Simulation results: conclusions

By conducting the extensive type I error and power simulations, we have demonstrated several critical points:

1. Baseline adjustment is necessary when the baseline value is a mediator for the effect of G on CFB (meaning in addition to the direct G effect on CFB, G can influence CFB through its association with the baseline value). Note that this may not inflate the entire QQ plot as the number of variants associated with the baseline should not be large. However, the type I error rates for those variants associated with the baseline will be inflated if the mediator effect is unaccounted for. Theoretically speaking, when the mediator effect is unaccounted for, we are testing $\beta _{y_0} \ast \beta _{G_0} + \beta _G$ in the unadjusted model. Even if the parameter of interest $\beta _G = 0$, a non-zero $\beta _{y_0} \ast \beta _{G_0}$ can still drive a spurious association.
2. The baseline adjustment model can control the type I error under various scenarios, even when there are normal (white noise) random errors added to the baseline and post-baseline measurements.
3. The power comparison between the baseline-adjusted and unadjusted models depends on the mediator effect ($\beta _{y_0} \ast \beta _{G_0}$) and the genetic effect ($\beta _G$). If they are in different directions, the baseline-adjusted model has higher power. If they are in the same direction, the unadjusted model’s power is higher before adjusting for inflated type I errors. In general, if $|\beta _{y_0} \ast \beta _{G_0} + \beta _G| > |\beta _G|$, the power of the unadjusted model is higher compared to the adjusted model, though at the cost of higher type I error as noted in 1).
4. The joint test (2df test) of $\beta _G = 0 \, \& \, \beta _{GT} = 0$ is more desirable if we would like to detect strong interaction between treatment and genotype. When we don’t expect or are not interested in the interaction, such as in Oni-Orisan et al.’s paper^6^, a 1df test of $\beta _G = 0$ is more powerful.

### IMPROVE-IT GWAS analysis results from nine statistical models

**Fig. 3:**
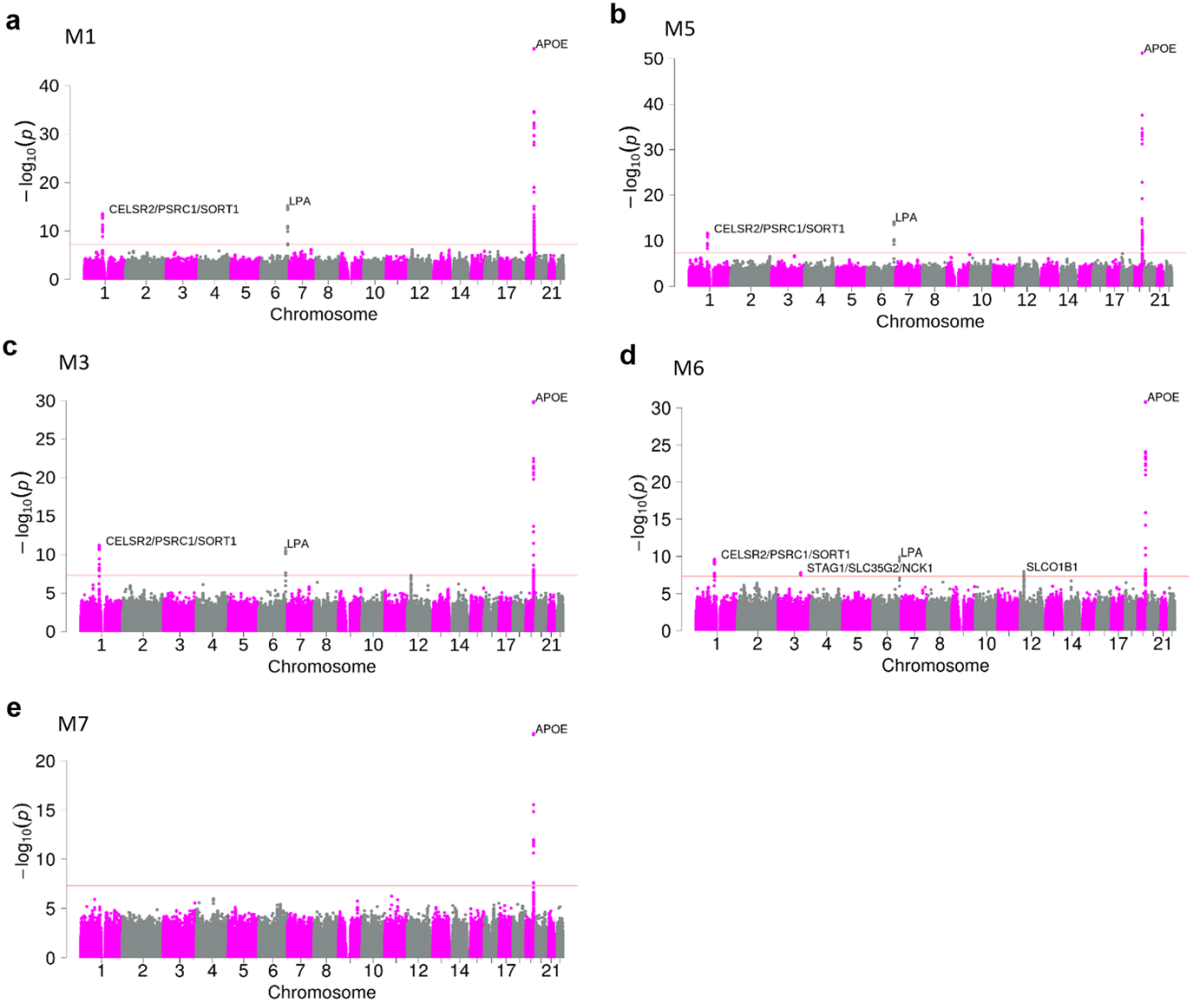
Manhattan plots for five genome-wide association studies (GWAS) of drug-induced change in low-density lipoprotein cholesterol (LDL-C) from IMPROVE-IT PGx study. These five Manhattan plots are from Model 1 (**a**, M1), Model 5 (**b**, M5), Model 3 (**c**, M3), Model 6 (**d**, M6) and Model 7 (**e**, M7). M1 used log-fold-CFB as phenotype, adjusted for baseline LDL-C and used a 2-step approach in the regression, in which residuals were obtained by regressing out the covariates and then inverse normally transformed. M3 was the same as M1 except that it did not adjust for baseline LDL-C in the model. Both M1 and M3 yielded the same three significant loci. M5 used log-fold-CFB as the phenotype, adjusted baseline LDL-C, and used the 2-df test in the regression. M6 was the same as M5 except that it did not adjust for baseline LDL-C in the model. M5 yielded three significant loci while M6 yielded two additional significant loci (*STAG1/SLC35G2/NCK1*, *SLCO1B1*) on chromosome 3 and 12, respectively. M7 used log-baseline as the phenotype for the baseline association test, which yielded one significant locus on chromosome 19. The horizontal red line represents the whole-genome significant p value threshold 5e−08. All tests were two-sided.

There was no evidence of overall genomic inflation^8^ based on a genomic inflation factor (*λ*) value in this European population with 5661 subjects. The genomic inflation factors from the nine GWAS analyses based on the nine statistical models (Methods) are 1.02, 1.01, 1.01, 1.01, 1.00, 1.00, 1.01, 1.02 and 1.01, respectively (Supplementary Fig. 10). The association results from the nine GWAS analyses of LDL-C response are summarized in Fig. 3, Supplementary Fig. 11, Table 1 and Supplementary Table 1. Five lead variants in the genome-wide significant loci (*CELSR2*/*PSRC1*/*SORT1*, *STAG1*/*SLC35G2*/*NCK1*, *LPA*, *SLCO1B1* and *APOE*) were detected by at least one of the nine GWASs from the nine statistical models. The GWAS using the log-fold-CFB adjusting for the natural log-transformed baseline level (statistical model M1) revealed variants from three loci that met genome-wide significance (*CELSR2*/*PSRC1*/*SORT1*, *LPA* and *APOE*; Fig. 3a and Table 1). The GWAS from M3 (same as M1, but without baseline adjustment) yielded the same three loci (Fig. 3c and Table 1) that met genome-wide significance, though with larger *p* value and smaller $\beta _{G_0}$. The smaller *p* values for the adjusted models are consistent with the simulation results that suggest power will be larger for baseline-adjusted models when the mediator effect $( {\beta _{y_0} \ast \beta _{G_0}} )$ is in the opposite direction from $\beta _G$. Similar results were observed when the CFB ratio was used as the endpoint: both the baseline-adjusted model M4 and the unadjusted model M2 yielded the same three loci (Supplementary Fig. 11-M4 and M2 and Supplementary Table 1), and unadjusted models resulted in larger *p* values and smaller $\beta _{G_0}$. It is worthwhile noting that the *CELSR2*/*PSRC1*/*SORT1* locus was identified in all four models (M1, M3, M5 and M6) in our GWAS (Fig. 3a–d) while it was not detected by the baseline-unadjusted models in Oni-Orisan et al.’s paper^6^. In addition, we observed very small differences in use of baseline difference $\left( {\ln {{{\mathrm{y}}}}_1 - \ln {{{\mathrm{y}}}}_0} \right)$ vs. CFB ratio $\left( {\frac{{{{{\mathrm{y}}}}_1 - {{{\mathrm{y}}}}_0}}{{{{{\mathrm{y}}}}_0}}} \right)$ as a phenotype.

**Table 1:** All five lead variants from the GWAS analyses of natural log-transformed CFB of LDL-C, LDL-C percent change and natural log-transformed baseline LDL-C based on five statistical models: M1 (baseline adjustment, 1-df test and 2-step regression), M3 (baseline un-adjustment, 1-df test and 2-step regression), M5 (baseline adjustment and 2-df test), M6 (baseline un-adjustment and 2-df test) and M7 (baseline association only).

Gene	SNP	CHR	BP	MA	MAF	Model^a^	$\beta _{{{{\boldsymbol{y}}}}_0}$^b^	$\beta _{{{\boldsymbol{G}}}}$ or $\beta _{{{{\boldsymbol{G}}}}_0}$^c^	P_G	$\beta _{{{{\boldsymbol{GT}}}}}$^d^	P_GT^d^	P_2df^d^
*CELSR2*/*PSRC1*/*SORT1*	rs599839	1	109822166	G	0.240	M1 (BJ-1dfT-2SR)	−0.490	−0.172	2.92E−14			
						M3 (BuJ-1dfT-2SR)		−0.156	6.19E−12			
						M5 (BJ-2dfT)	−0.493	−0.052	1.12E−12	0.026	8.12E−02	2.22E−12
						M6 (BuJ-2dfT)		−0.049	1.74E−10	0.029	6.04E−02	2.44E−10
						M7 (Baseline only)		−0.008	6.58E−02^e^			
*STAG1*/*SLC35G2*/*NCK1*	rs79929954	3	136623748	G	0.015	M1 (BJ-1dfT-2SR)	−0.490	0.215	7.81E−03^e^			
						M3 (BuJ-1dfT-2SR)		0.214	7.91E−03^e^			
						M5 (BJ-2dfT)	−0.486	0.079	2.68E−03	−0.244	3.04E−06	2.05E−07^e^
						M6 (BuJ-2dfT)		0.080	3.23E−03	−0.285	1.78E−07	1.58E−08
						M7 (Baseline only)		−0.002	8.92E−01^e^			
*LPA*	rs10455872	6	161010118	G	0.080	M1 (BJ-1dfT-2SR)	−0.490	0.287	5.94E−16			
						M3 (BuJ-1dfT-2SR)		0.240	1.38E−11			
						M5 (BJ-2dfT)	−0.499	0.090	6.82E−15	−0.050	3.06E−02	6.53E−15
						M6 (BuJ-2dfT)		0.078	1.13E−10	−0.049	4.28E−02	1.20E−10
						M7 (Baseline only)		0.024	3.68E−04^e^			
*SLCO1B1*	rs4149056	12	21331549	C	0.162	M1 (BJ-1dfT-2SR)	−0.490	0.129	6.92E−07^e^			
						M3 (BuJ-1dfT-2SR)		0.141	5.20E−08^e^			
						M5 (BJ-2dfT)	−0.485	0.043	3.76E−07	−0.028	9.10E−02	5.95E−07^e^
						M6 (BuJ-2dfT)		0.050	1.33E−08	−0.036	3.75E−02	1.12E−08
						M7 (Baseline only)		−0.013	9.64E−03^e^			
*APOE*	rs1065853	19	45413233	T	0.089	M1 (BJ-1dfT-2SR)	−0.490	−0.491	2.13E−48			
						M3 (BuJ-1dfT-2SR)		−0.388	1.32E−30			
						M5 (BJ-2dfT)	−0.535	−0.164	3.91E−50	0.081	1.59E−04	5.87E−52
						M6 (BuJ-2dfT)		−0.130	7.99E−30	0.082	2.73E−04	1.52E−31
						M7 (Baseline only)		−0.064	2.35E−23			

*SNP* single nucleotide polymorphism, *CHR* chromosome, *BP* base pair, *MA* minor allele, *MAF* minor allele frequency, $\beta _{y_0}$ effect size of baseline variable, *β*_*G*_ effect size of G (genotype) on CFB (from M1, M3, M5 and M6), $\beta _{G_0}$ effect size of G (genotype) on baseline (from M7), *P_G* *p* value of G (genotype), *β*_*GT*_ effect size of G*T (genotype by treatment interaction), *P_GT* *p* value of G*T (genotype by treatment interaction), *P_2df* *p* value of 2df test (joint test of genotype and genotype*treatment interaction).

^a^BJ: Baseline-adjusted; BuJ: Baseline-unadjusted; 1dfT: 1 degree of freedom test; 2dfT: 2 degree of freedom test or joint test of genotype and genotype*treatment interaction; 1SR: 1-step regression; 2SR: 2-step regression. In M1, M3, M5 and M6, difference of natural log-transformed Simvastatin and Ezetimibe/Simvastatin on low-density lipoprotein cholesterol levels were used for analysis.

^b^Effects calculated for the nature log-transformed baseline LDL-C.

^c^Effects calculated with respect to the minor allele. A negative value indicates more intense drug (Simvastatin and Ezetimibe/Simvastatin) LDL-C lowering.

^d^Results were only available in the 2df test model M5 and M6, which also tests the genotype*treatment interaction and joint test of genotype and genotype*treatment interaction.

^e^Not reaching genome-wide significance (*p* < 5E−08). For 2df test methods, *p* values from the 2df test (P_2df) were used.

The 1-step regression with baseline adjustment model M8 gave the same three statistically significant loci (Supplementary Fig. 11-M8 and Supplementary Table 1) and M9, the unadjusted version of M8, yielded one more statistically significant locus (*SLCO1B1*) on chromosome 12 (Supplementary Fig. 11-M8 and Supplementary Table 1). This is consistent with our simulation results that the 1-step regression models provided slightly larger power than the 2-step regression models (e.g., M9 vs. M3 in Fig. 2). The smaller *p* value for the unadjusted models for the *SLCO1B1* locus (Table 1) could be explained by the fact that the unadjusted model has higher power for detecting the signals whose mediator effect is in the same direction with the genetic effect; for *SLCO1B1*, the effect allele for the lead variant was associated with a higher baseline LDL-C and lower treatment benefit. However, it is quite possible that this power advantage is (at least partially) due to inflated type I error as evidenced in the simulation results. In addition, when the 2df test (joint test of G and GT interaction) was used, the baseline-adjusted model M5 detected the same loci (Fig. 3b and Table 1) as M1 and M8. M6, the unadjusted 2df test model, was able to identify one more locus (*STAG1*/*SLC35G2*/*NCK1*; Fig. 3d and Table 1) as compared to M9 and M3. The strong genotype*treatment interaction effect (*β*_*GT*_ in Table 1) and the small interaction *p* value (P_GT in Table 1) for this locus explains why it was only detected with the 2df test.

**Fig. 4:**
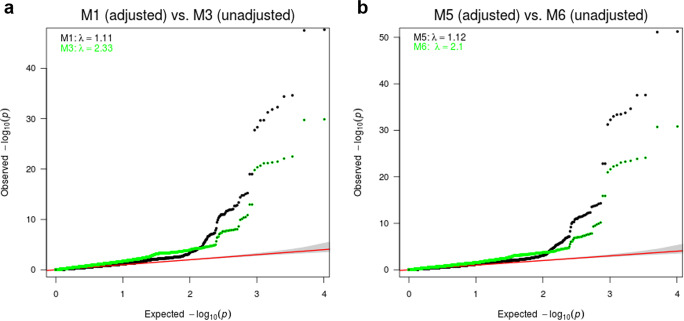
QQ plots of the *p* values between two sets of the baseline-adjusted models (black) vs. the baseline-unadjusted models (green) from the four GWAS analyses based on four models with log-CFB endpoint. M1 vs. M3 (**a**) M5 vs. M6 (**b**). The variants were first filtered based on the baseline association *p* value <1e−03 from M7 and 10,187 SNPs were used for both plots. These variants showed clear mediator effect $\beta _{G_0} \ast \beta _{y_0} \ne 0$. The red line was the diagonal line and the 95% confidence interval polygon in each QQ plot was based on the *p* values from the baseline-adjusted model (M1 in **a** and M5 in **b**).

To further explore the impact of baseline adjustment on type I error, we selected a subset of 10,187 variants whose baseline association *p* values are less than 1e−03 from M7 (the baseline association model). Given a clear negative correlation between the baseline and the CFB in IMPROVE-IT data (Supplementary Fig. 3), it is reasonable to assume that these variants show some evidence of mediator effect, i.e., $\beta _{G_0} \ast \beta _{y_0} \,\ne\, 0$. Figure 4 and Supplementary Fig. 12 summarize the quantile-quantile plots of these variants’ *p* values generated by M1–M9 except for M7. The comparisons uniformly showed that baseline-unadjusted models had much larger genomic inflation factors (*λ* = 2.10–2.47) than the baseline-adjusted models (*λ* = 1.10–1.19). This observation is consistent with our simulation results that the baseline-unadjusted models can heavily inflate the type I errors for those variants with non-zero mediator effect. The baseline-adjusted models, however, control type I error well regardless of the mediator effect.

To further examine the influence of baseline adjustment on power, we compared the top variants’ association results that were identified by the baseline-adjusted and unadjusted models respectively. Table 1 shows that as long as the estimated $\beta _{y_0} \ast \beta _{G_0}$ is of a different sign than the estimated $\beta _G$, then the baseline-adjusted models will have smaller *p* values than the corresponding unadjusted models (e.g., while comparing M3 vs. M1, M2 vs. M4, M6 vs. M5 and M9 vs. M8). The only exception is rs79929954 (M1 vs. M3) in the *STAG1*/*SLC35G2*/*NCK1* locus whose baseline association *p* value is $P_{G_0} = 0.892$ and $\beta _{G_0} = - 0.002$. However, this observation is consistent with our simulation results that the baseline-adjusted and unadjusted models provide very similar power (*p* values from M1 and M3 are 7.81E−03 and 7.91E−03, respectively) if there is no genotype and baseline association (or $\beta _{G_0}$ is very close to 0). We also observed that the smaller the $P_{G_0}$ (e.g., larger the association of variant and baseline), the larger difference of association strength (i.e., the CFB association *p* values) tends to be observed between the baseline-adjusted and unadjusted models in our LDL-C real GWAS example (given $\beta _{y_0}$ is a negative value around −0.5). For instance, the baseline-genotype association of the rs1065853 SNP in the *APOE* locus was very strong ($\beta _{G_0}$ = −0.064 and P_G = 2.35E−23), which led to the large difference between the baseline-adjusted model M1 (P_G = 2.13E−48) and the baseline-unadjusted model M3 (P_G = 1.32E−30). In contrast, when there was no association between the baseline and the rs79929954 SNP in the *STAG1*/*SLC35G2*/*NCK1* locus ($\beta _{G_0}$ = −0.002 and P_G = 8.92E−01), we observed little difference between the baseline-adjusted mode M1 (P_G = 7.81E−03) and the baseline-unadjusted mode M3 (P_G = 7.91E−03). On the other hand, the baseline-unadjusted models detected two loci that the adjusted models failed to detect: *STAG1*/*SLC35G2*/*NCK1* (M6) and *SLCO1B1* (M6 and M9). In both cases, the estimated $\beta _{y_0} \ast \beta _{G_0}$ are in the same direction as the estimated $\beta _G$, which is the signal pattern that favors the unadjusted model as shown in the simulation. However, these two loci may not remain statistically significant if we take the potential type I error inflation of the unadjusted models into consideration.

GWAS analyses using the 2df test models (or the joint test of $\beta _G = 0 \, \& \, \beta _{GT} = 0$, M5 and M6) confirmed that the 2df test is more powerful than the 1df test when there is a large interaction effect between the SNP and treatment. For example, the 2df test provided much stronger evidence of association (M5 *p* = 5.95E−07, M6 *p* = 1.12E−08) for SNP rs79929954 in the *STAG1*/*SLC35G2*/*NCK1* locus, while the *p* values of the 1df test models (M1–M4, M8 and M9) were at 1E−03 level since there was a strong genotype by treatment interaction effect (P_GT = 1.78E−07 in M6, Table 1 and Fig. 1). A similar pattern was observed for SNP rs4149056 in the *SLCO1B1* locus (M6 *p* = 1.12E−08 < M9 *p* = 1.33E−08) due to the moderate genotype by treatment interaction effect (P_GT = 3.75E−02 in M6, Table 1 and Fig. 1).

## Discussion

Using statistical simulations, we explore the impact of baseline adjustment in statistical models analyzing the association between genetic variants and change in quantitative measures between pre- (baseline) and post-treatment values. We find that, in situations in which a genetic variant is associated with both the baseline and CFB values and where there is a correlation between baseline and CFB, failure to adjust for the baseline value results in substantial inflation of test statistics. This inflation will result in not only higher risk of false positive associations between genotype and CFB, but also inaccurate assessment of the magnitude of quantitative CFB driven by genotype. We further evaluate the impact of normally distributed measurement errors and find that they may indeed inflate the type I error of baseline-adjusted models in the (unlikely) situation where the measurement errors are qualitatively different in the baseline and post-baseline. However, when the measurement errors are white noises or they are of the same sign, the measurement errors have little impact on the type I error of baseline-adjusted models. We acknowledge that there may be type I error inflation in baseline-adjusted models caused by other types of measurement errors not modeled here. Further research is clearly needed to fully understand the inflation caused by measurement errors in other scenarios. On the other hand, the type I error inflation of baseline-unadjusted models caused by the mediator effect is found in our simulation and in the IMPROVE-IT GWAS analysis as well (Fig. 4 and Supplementary Fig. 12). For those variants with at least some association with baseline values, the baseline-unadjusted models show clear type I error inflation while the baseline-adjusted models do not. This observation suggests that the mediator effect of the baseline should be considered in order to properly control type I errors.

Multiple approaches have been recommended in the literature to control for this type of mediator effect, including use of the ratio of change as an endpoint instead of or in addition to including the baseline value as a covariate in the model. Consistent with prior reports^6^, we find that use of the CFB ratio without baseline adjustment is not sufficient to address the mediator effect. Analysis of post-treatment values directly with baseline values included as a covariate is also recommended in the literature as an approach to address the mediator effect; this approach was also tested by ref. ^6^. Because this model is similar to the log-fold change from the baseline model with adjustment of coefficients by a constant^7^, we did not test this model separately in this work.

In addition to the baseline adjustment, other aspects of the statistical models also play an important role in the analysis of quantitative change. No single approach is universally the most powerful test for all the different signal patterns as shown in the power comparison between the 2df test and 1df test. The 2df joint test of $\beta _G = 0 \, \& \, \beta _{GT} = 0$ is the most robust test to identify a potential interaction effect (Supplementary Fig. 7) and is recommended when the data include both treatment and control arms and no prior information available regarding interaction with the genotype. The 2df test can be used as the screening step in a GWAS to assess the combined prognostic and predictive association of each genetic variant to drug response to declare statistical significance. To help interpret 2df test results, it is also recommended to generate the *p* value and effect estimate for the genotype and the interaction separately. All these results provide a comprehensive picture of any signals associated with drug response.

Compared with a 2-step regression approach, 1-step regression provides similar or slightly larger power. However, a 2-step regression approach generally runs faster than a 1-step regression approach in GWAS analysis since, for each SNP, only a univariate regression is needed. For 1-step regression, the phenotype needs to be appropriately transformed so that the type I error can be controlled. For a 2-step regression, appropriate transformation of the residuals is necessary to make valid statistical inference, for example, inverse normal transformation of the residuals to ensure normal distribution before the second step for genotype association analysis (Supplementary Figs. 13 and 14). For more discussion on the 2-step vs. 1-step regression and more robust approaches by combining both, please refer to McCaw et al.’s paper ^9^.

In addition to the simulations, we also explored the various statistical models in a real PGx analysis dataset, using data from the IMPROVE-IT study of cholesterol lowering drugs (statin vs. statin + ezetimibe). Despite differences in the patient population and in treatments received for a subset of patients, the GWAS results from Oni-Orisan et al.’s paper^6^ and our IMPROVE-IT are largely consistent. In both analyses, the loci at *SORT1*, *LPA*, and *APOE* become more significant after baseline adjustment. Oni-Orisan et al. argued that the *SORT1* locus was a false positive because a heterogeneity *Q* test was not able to replicate the finding^6^. Our simulation results showed that this *Q* test is less powerful compared with other more commonly used models. Thus, it is not surprising that the heterogeneity *Q* test may not replicate the results from the adjusted models. In our dataset, the *SORT1* locus was clearly associated with change in LDL-C with treatment in all statistical models used, suggesting that this locus is in fact associated with response to lipid-lowering treatment. Interestingly, the *SORT1* locus was strongly associated with baseline LDL-C in Oni-Orisan et al.’s paper^6^, but only weakly trended to an association with baseline LDL-C in the IMPROVE-IT dataset, perhaps because the locus is more strongly associated with variability in LDL-C levels in among those first initiating statin treatments rather than those in a high-risk population as enrolled in the IMPROVE-IT study. Conversely, the *LPA* locus showed only a nominally significant association with baseline LDL-C in Oni-Orisan et al.’s paper^6^ but was clearly associated with baseline LDL-C in the IMPROVE-IT dataset. Again, differences in the patient population may explain these observations. Two loci at *LDLR* and *APOB* just met genome-wide significance thresholds in Oni-Orisan et al.’s paper (in their baseline-adjusted analyses)^6^ but were not detected in the IMPROVE-IT analyses (Supplementary Table 2); this is not surprising given the smaller sample size in the IMRPOVE-IT dataset. These loci have not been associated with LDL change in other statin response GWASs, though the dataset used in Oni-Orisan et al.’s paper is larger than those previously analyzed.

From the perspective of type I error control, selecting baseline-unadjusted vs. adjusted models for PGx CFB analysis is a trade-off between mitigating measurement error problems and accounting for the mediator effect. Our simulations and IMPROVE-IT GWAS analyses demonstrate the impact of the mediator effect on analysis results, resulting in higher type I errors in unadjusted models under the simulation settings tested. However, in our simulations under certain conditions the power of the unadjusted models was slightly higher than that of the adjusted models, and the type I errors from adjusted models were higher. Further, while ideally a quantitative change analysis would rely on multiple repeated measures to minimize the impact of measurement errors, we assume for most large studies repeated measurements are not likely to be generated. Therefore, guided by the results from our extensive simulations and real GWAS analyses, we recommend performing baseline-adjusted analyses as the primary analysis. Baseline-unadjusted analyses may be conducted only if the type I error rate of baseline-adjusted model is inflated, which may be caused by measurement error of very specific type (e.g., different directions of the error baseline vs. post-baseline). In addition, we recommend use of the most general 2-df test statistical model for PGx studies with both treatment and control arms, especially when it is expected that there are strong G*T interaction effects for some markers and/or when there are underlying genetic related difference between the two arms. Finally, careful post-GWAS biological and clinical interpretation along with independent replication are essential in separating false positives from true association signals.

## Methods

### Statistical models for GWAS analysis

Nine statistical models are considered for our simulations and real GWAS analyses. Assume ${{{\mathrm{y}}}}_1$ is the on-treatment response value and ${{{\mathrm{y}}}}_0$ is the baseline response values, these nine models are:

- M1 (log-fold-CFB, adjusted, 1df test, 2-step: INT residuals): $\ln {{{\mathrm{y}}}}_1 - \ln {{{\mathrm{y}}}}_0\sim \beta _0 + \beta _{y_0}\ln {{{\mathrm{y}}}}_0 + \beta _T{{{\mathrm{T}}}} + \beta _X{{{\mathrm{X}}}}$; ${\rm{INTed}}\,{\rm{Res}}\sim \beta _G{{{\mathrm{G}}}}$,
- M2 (CFB ratio, unadjusted, 1df test, 2-step: INT residuals): $\frac{{{{{\mathrm{y}}}}_1 - {{{\mathrm{y}}}}_0}}{{{{{\mathrm{y}}}}_0}}\sim \beta _0 + \beta _T{{{\mathrm{T}}}} + \beta _X{{{\mathrm{X}}}}$; ${\rm{INTed}}\,{\rm{Res}}\sim \beta _G{{{\mathrm{G}}}}$,
- M3 (log-fold-CFB, unadjusted, 1df test, 2-step: INT residuals): $\ln {{{\mathrm{y}}}}_1 - \ln {{{\mathrm{y}}}}_0\sim \beta _0 + \beta _T{{{\mathrm{T}}}} + \beta _X{{{\mathrm{X}}}}$; ${\rm{INTed}}\,{\rm{Res}}\sim \beta _G{{{\mathrm{G}}}},$
- M4 (CFB ratio, adjusted, 1df test, 2-step: INT residuals): $\frac{{{{{\mathrm{y}}}}_1 - {{{\mathrm{y}}}}_0}}{{{{{\mathrm{y}}}}_0}}\sim \beta _0 + \beta _{y_0}{{{\mathrm{y}}}}_0 + \beta _T{{{\mathrm{T}}}} + \beta _X{{{\mathrm{X}}}}$; ${\rm{INTed}}\,{\rm{Res}}\sim \beta _G{{{\mathrm{G}}}}$,
- M5 (log-fold-CFB, adjusted, 2df test, 1-step): $\ln {{{\mathrm{y}}}}_1 - \ln {{{\mathrm{y}}}}_0\sim \beta _0 + \beta _{y_0}\ln {{{\mathrm{y}}}}_0 + \beta _T{{{\mathrm{T}}}} + \beta _G{{{\mathrm{G}}}} + \beta _{GT}\left( {{{{\mathrm{GxT}}}}} \right) + \beta _X{{{\mathrm{X}}}}$,
- M6 (log-fold-CFB, unadjusted, 2df test, 1-step): $\ln {{{\mathrm{y}}}}_1 - \ln {{{\mathrm{y}}}}_0\sim \beta _0 + \beta _T{{{\mathrm{T}}}} + \beta _G{{{\mathrm{G}}}} + \beta _{GT}\left( {{{{\mathrm{GxT}}}}} \right) + \beta _X{{{\mathrm{X}}}}$,
- M7 (baseline association): $$\ln {{{\mathrm{y}}}}_0\sim \beta _0 + \beta _{G_0}{{{\mathrm{G}}}} + \beta _X{{{\mathrm{X}}}}$$,
- M8 (log-fold-CFB, adjusted, 1df test, 1-step): $\ln {{{\mathrm{y}}}}_1 - \ln {{{\mathrm{y}}}}_0\sim \beta _0 + \beta _{y_0}\ln {{{\mathrm{y}}}}_0 + \beta _T{{{\mathrm{T}}}} + \beta _G{{{\mathrm{G}}}} + \beta _X{{{\mathrm{X}}}}$,
- M9 (log-fold-CFB, unadjusted, 1df test, 1-step): $\ln {{{\mathrm{y}}}}_1 - \ln {{{\mathrm{y}}}}_0\sim \beta _0 + \beta _T{{{\mathrm{T}}}} + \beta _G{{{\mathrm{G}}}} + \beta _X{{{\mathrm{X}}}}$.

where X are covariates, G is genotype, T is treatment, $\beta _0$ is intercept, $\beta _{y_0}$ is baseline coefficient, $\beta _T$ is treatment coefficient, $\beta _X$ are covariates’ coefficients, $\beta _{G_0}$ is genotype coefficient for baseline (from M7), $\beta _G$ is genotype coefficient for CFB (from M1–M6 and M8–M9), $\beta_{GT}$ is genotype by treatment interaction coefficient, ln is natural log transformation function and “INTed Res” represents the inverse normal transformed residuals.

The first four models M1–M4 are the same as the four models M_a_–M_d_ discussed in Oni-Orisan et al.’s paper^6^, which use the one degree of freedom (1df) tests for the main genotype effect assuming no genetic-by-treatment interaction. M1 and M3 model the log-fold-CFB with and without baseline adjustment respectively. M4 and M2 are similarly defined for the phenotype of the CFB ratio. All these four models take a 2-step regression approach. In the first step, residuals are obtained by regressing the response on non-genetic covariates including the treatment (*T*), covariates ***X*** and the baseline variable (if needed). The residuals are further inverse normal transformed (INTed). In the second step, the (INTed) residuals are directly regressed on the genotype (G). M5 and M6 use the two degree of freedom (2df) tests for modeling the log-fold-CFB with or without baseline adjustment, respectively. In PGx studies with more than one arm, patients’ clinical outcomes could be influenced by both main genotype effect and genotype by treatment interaction effects. The 2df joint test of the main effect and the interaction effect usually increases power for detecting signals in PGx studies with small to moderate sample sizes compared with only testing the interaction effect or the main genotype effect separately^10^. M7 is the baseline association model. To evaluate the impact of the 2-step and the 1-step regression approaches, we also run two additional models M8 and M9 with 1-step regression in parallel with M1 and M3 with 2-step regression for the log-fold-CFB.

It is straightforward to see that M5 is the most general out of these nine models. M1 (M8) is a special case of M5 when $\beta _{GT} = 0$ while M6 is a special case when $\beta _{y_0} = 0$. M_3_ is even more restricted by fixing $\beta _{GT}$ and $\beta _{y_0} = 0$ at the same time. It is worth mentioning that mis-specifying $\beta _{GT}$ may only affect the power performance but fixing $\beta _{y_0} = 0$ could inflate the type I errors as evidenced in the simulation section.

In addition to the aforementioned models of CFB, we also considered the following models of post-baseline measurements and Cochran’s *Q* test as a sensitivity analysis.

- M5* (log-post-treatment, adjusted, 1df test, 1-step): $\ln {{{\mathrm{y}}}}_1\sim \beta _0 + \beta _{y_0}\ln {{{\mathrm{y}}}}_0 + \beta _T{{{\mathrm{T}}}} + \beta _G{{{\mathrm{G}}}} + \beta _X{{{\mathrm{X}}}}$,
- M6* (log-post-treatment, unadjusted, 1df test, 1-step): $\ln {{{\mathrm{y}}}}_1\sim \beta _0 + \beta _T{{{\mathrm{T}}}} + \beta _G{{{\mathrm{G}}}} + \beta _X{{{\mathrm{X}}}}$,
- Cochran’s *Q* test (as described in Neupane et al.’s paper^11^)*:* Let $\beta _{Gi},i = 0,1,$ be the regression coefficients of $G$ in the model $\ln {{{\mathrm{y}}}}_{{{\mathrm{i}}}}\sim \beta _0 + \beta _T{{{\mathrm{T}}}} + \beta _{Gi}{{{\mathrm{G}}}} + \beta _X{{{\mathrm{X}}}},\;i = 0,1$, respectively. The Cochran’s *Q* statistic is defined as $Q = \mathop {\sum }\nolimits_{i = 0}^1 \omega _i( {\hat \beta _{Gi} - \hat \beta } )^2$, where $\hat \beta = \mathop {\sum }\nolimits_{i = 0}^1 \omega _i\hat \beta _{Gi}/\mathop {\sum }\nolimits_{i = 0}^1 \omega _i$, $\omega _i = 1/s_i^2$, $s_i^2$ is the estimated variance of $\hat \beta _{Gi}$.

We can verify that the *Q* statistic is equal to $( {\hat \beta _{G1} - \hat \beta _{G0}} )^2\!/\!(s_0^2 + s_1^2)$ which follows a chi-squared distribution with degree of freedom 1 if these two betas are independent. However, in our context, they are likely to be positively correlated. Thus, $s_0^2 + s_1^2$ overestimates the variance of $\hat \beta _{G1} - \hat \beta _{G0}$ which makes the *p* values calculated by chi-squared distribution too conservative. Indeed, our simulation results confirm that *Q* test is not powered to detect CFB association.

### Simulation settings

We simulate the baseline $y_0$ and the CFB from M5 and M7, respectively, in the following way:

1 $$\ln {{{\mathrm{y}}}}_0 = 4.6 + \beta _{G_0}{{{\mathrm{G}}}} + {\it{\epsilon }}_0,$$

2 $$\ln {{{\mathrm{y}}}}_1 - \ln {{{\mathrm{y}}}}_0 = - 0.25{{{\mathrm{T}}}} - 0.01{{{\mathrm{X}}}} + \beta _{y_0}\ln {{{\mathrm{y}}}}_0 + \beta _G{{{\mathrm{G}}}} + \beta _{GT}{{{\mathrm{GT}}}} + {\it{\epsilon }}_1,$$

where treatment variable $T \sim {\rm{bernouli}}(0,1)$, age variable $X \sim {\rm{uniform}}(18,65)$, genotype $G \sim {\rm{binomial}}(n,2,\;{\rm{MAF}})$ with sample size $n = 1000$, ${\rm{MAF}} = 0.2.$ The regression errors ${\it{\epsilon }}_0$ and ${\it{\epsilon }}_1$ are assumed to be independent and follow a standard normal distribution. Parameters $\beta _{G_0}$ measures the effect size of the baseline association, $\beta _{y_0}$ measures the dependence of CFB on the baseline. $\beta _G$ and $\beta _{GT}$ measure the direct genetic main effect and interaction effect on CFB, respectively. Under $H_0$, we consider $\beta _{G_0} = - 0.1,\;0,\;0.1$, $\beta _{y_0} = - 0.2,\;0,\;0.2$. Under $H_1,$ in addition to the settings under $H_0$, we consider each of $\beta _G$ and $\beta _{GT}$ to be $- 0.4, - 0.3, \ldots ,0.4,$ respectively. We also consider measurement errors. Specifically, we assume the relative error rates to be independent normal variables, $r_i \sim N\left( {\mu _i,\sigma = \frac{1}{4}} \right),i = 0,1.$ Three configurations of $\mu _i$ were used: (1) $\mu _i \equiv 0$ (white noise); (2) $\mu _i \equiv 0.25$ (biased toward the same direction); (3) $\mu _0 = - 0.25$, $\mu _1 = 0.25$ (biased toward different direction). Then, the observed responses under measurement errors are $\left( {1 + r_0} \right)y_0$ and $(1 + r_1)y_1$. Such variable will generate error rates between −0.49 to 0.49 with 95% probability. In the rare cases, the simulated responses are also bounded from below at 1 to ensure a valid log transform. We simulate $2 \times 10^7$ repetitions under the null hypotheses and $2 \times 10^3$ repetitions under the alternative hypotheses. We compare the type I error and power of M1–M9 except for M7. The empirical type I error and power are calculated as the proportion of *p* values less than a nominal level $\alpha$. For type I error comparison, we also calculated the variance of empirical type I error as $\sigma ^2 = \alpha (1 - \alpha )/n$^12^. A margin of error $= 3\sigma$ was used to determined if the empirical type I error is inflated, i.e., significantly higher than the nominal level.

### Mediator effect

**Fig. 5:**
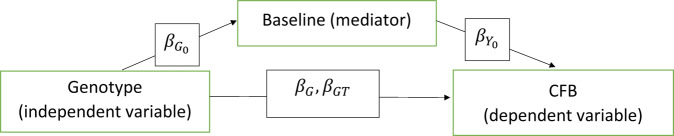
Illustration of baseline as a mediator effector in the analysis of CFB in PGx studies. This mediator effector, if existing, must be accounted for, e.g., by baseline adjustment.

Plugging Eq. (1) into Eq. (2), we can see that the $G$ effect on CFB is $\beta _G + \beta _{G_0}\ast\beta _{y_0}$. Under the null hypothesis that $\beta _G = 0$, $G$ is still associated with CFB by $\beta _{G_0} \ast \beta _{y_0}$, which is called the mediator effect. The mediator, in our model, is the baseline measurement, e.g., the SNPs not only influences the CFB directly but also by influencing the baseline. This mediator effect must be accounted for, e.g., by baseline adjustment, to correctly estimate the $\beta _G$, otherwise it will inflate the type I error rates. The underlying relationship between genotype and CFB, which is mediated by the baseline variable, is described in Fig. 5.

### Analysis of IMPROVE-IT PGx GWAS data

We applied the nine statistical models to GWAS analyses of Ezetimibe response in IMPROVE-IT. In this PGx study, we were interested in discovering genetic variants that influence efficacy of Vytorin (EZ + simva) treatment to further identify subpopulations who would receive a greater benefit from Vytorin treatment using clinical data from IMPROVE-IT. The details of the endpoint, genotyping, genotype QC and imputation for this GWAS analyses are described below. After GWAS QC and SNP imputation, there were 9,407,967 variants and 6502 subjects available for analyses. The subjects were further filtered by excluding subjects who had cardiovascular event prior to month 1 since cardiovascular event affected LDL-C that may not be treatment related. A total of 5661 European subjects were included for GWAS analyses. For all genome-wide analyses, *p* < 5 × 10^−8^ was considered as the threshold to meet genome-wide significance. Statistical analyses were conducted with R (R Foundation for Statistical Computing, version 3.5.2, https://www.R-project.org/) and PLINK (version 1.07, http://pngu.mgh.harvard.edu/purcell/plink/)^13^. All statistical tests were two-sided.

### IMPROVE-IT trial analysis

#### Data source and study population

The clinical data in this PGx population for the Ezetimibe response GWAS analysis were collected from the IMPROVE-IT (IMProved Reduction of Outcomes: Vytroin Efficacy International Trial, clinical trial registry number: NCT00202878), which is a multi-center, double-blind, randomized phase 3b study to establish the efficacy and safety of Vytorin (ezetimibe + simvastatin tablet) in comparison to simvastatin monotherapy in 18,144 high-risk patients who were presenting with acute coronary syndrome (ACS) and age 50 and older^14^. Subjects were required to have their LDL-C levels between 50–125 mg/dl at the time of the qualifying event (QE) if they had not been taking any lipid-lowering therapies, or 50–100 mg/dl if they had been receiving lipid-lowering therapies. All subjects entering the study were randomized in 1:1 ratio to receive either ezetimibe 10 mg/simvastatin 40 mg combination or simvastatin 40 mg QD. Patients returned for follow up visits at 1 month, 4 months, and every 4 months after that. The trial was specified to end after all subjects had a follow up for a minimum of 2.5 years (median follow up was 6 years), and a primary endpoint event had been documented in at least 5250 subjects. The ethics committee at each participating center approved the protocol and amendments^14^. All trials were carried out in accordance with the Declaration of Helsinki, current guidelines on Good Clinical Practices and local ethical and legal requirements. All participants provided voluntary written informed consent before trial entry.

#### Phenotype and statistical analysis

For baseline LDL-C (LDL-C at QE), measurement was obtained within 24 h from the time of presentation to best reflect the subject’s lipid status at the time of the event. In the cases where such measurement could not be collected, LDL-C measurements that were collected within 6 months prior to the QE were used. For a direct comparison with the response to treatment phenotypes used in Oni-Orisan et al.’s paper^6^, we used the same formulas to define the two phenotypes. The first phenotype was defined as log-fold change of LDL-C from QE to 1 month (i.e., ln(LDL-C at 1 month/LDL-C at QE)) or $\ln {{{\mathrm{y}}}}_1 - \ln {{{\mathrm{y}}}}_0$, the difference between the natural log-transformed baseline LDL-C $\left( {{{{\mathrm{y}}}}_0} \right)$ and on-treatment LDL-C (${{{\mathrm{y}}}}_1$) values (log-fold-CFB). The second phenotype was defined as CFB ratio or $\frac{{{{{\mathrm{y}}}}_1 - {{{\mathrm{y}}}}_0}}{{{{{\mathrm{y}}}}_0}}$, which is the percentage of the difference between the baseline LDL-C (${{{\mathrm{y}}}}_0$) and on-treatment LDL-C (${{{\mathrm{y}}}}_1$) values over the baseline values. Both phenotypes were adjusted for the following prespecified covariates: age, gender, prior lipid-lowering therapy, early ACS trial treatment variable (unenrolled, enrolled receiving test treatment, or enrolled receiving control treatment) and high-risk ACS diagnosis. The top five genetic ancestry eigenvectors (principal components (PC)) estimated from the GWAS data were also included as covariates to adjust for population structure. To investigate the impact of adjusting for baseline LDL-C values, both phenotypes of LDL-C response were performed with and without baseline adjustment. This resulted in four statistical regression models (M1, M3, M2 and M4) for GWAS analyses, which are based on genotype test only (or 1df test) and the 2-step regression procedure (see “Statistical models for GWAS analysis” section for details). To further compare this 1df test with the 2df test approach (by adding the genotype by treatment interaction in the model) and the 2-step regression procedure with the 1-step regression procedure, we used the other four statistical regression models (M5, M6, M8 and M9) for GWAS analyses of the log-fold-CFB endpoint as well. More details are discussed in the “Statistical models for GWAS analysis” section.

#### Genotyping, quality control and imputation

Regarding generation and quality control (QC) of genetic data, 7971 patients who provided appropriate consents for the genetic studies and had DNA with sufficient quality were genotyped using a custom Axiom™ array (Thermo Fisher Scientific, CA, USA). Genotyping was performed by BioProcessing Solutions Alliance (Piscataway, NJ, USA), and the raw data was processed following the vendor recommended guidelines. Genetic QC steps were performed in 6765 self-reported European subjects. Variants with missing call rate greater than 3%, as well as non-autosomal variants were excluded. Following variant QC, individuals were excluded if they had low call rates (missingness greater than 5%), gender mismatches, heterozygosity greater than 3 standard deviations from the mean of the Caucasian population, or identical and first/second degree relatives using identity-by-descent (>0.1875). Principal component analysis (PCA) was performed in a subset of variants pruned for linkage disequilibrium using EIGENSTRAT smartpca^15^, together with 1000 Genomes phase 3 data^16^ as the reference. Individuals who deviated ±6 standard deviation from the means of 1000 Genomes European super population for PC 1–3 were also removed. A second PCA was then performed with European-only individuals to calculate the eigenvalues to be used as covariates in the genetic analyses to correct for within population stratification. In total, 6502 individuals of European descent, and 644,570 directly genotyped variants have been used for imputation. Imputation was performed using IMPUTE2^17^ and 1000 Genomes European panel as the reference. In total, 9,407,967 variants with minor allele frequency greater than 0.01, and imputation quality score (*r*^2^) greater than 0.3 were used for the subsequent genome-wide association studies (GWAS) analyses.

### Reporting summary

Further information on research design is available in the Nature Research Reporting Summary linked to this article.

## Supplementary information

Final supplemental document

Reporting Summary

## Data Availability

MSD’s data sharing policy, including restrictions, is available at http://engagezone.msd.com/ds_documentation.php. Requests for access to the GWAS summary statistics results from this IMPROVE-IT clinical study data can be submitted through the EngageZone site or via email to dataaccess@merck.com.

## Code availability

No new algorithms or methods were developed for this analysis. All analyses conducted used standard statistical methods as outlined in the Methods section and key tables and figures. Example R code for statistical analysis can be provided upon request.
