# Supplementary material for: A statistical perspective on baseline adjustment in pharmacogenomic genome-wide association studies of quantitative change
Source: NPJ Genom Med. 2022 Jun 9;7:33. doi: 10.1038/s41525-022-00303-2 (PMC9184591; doi:10.1038/s41525-022-00303-2)
Supplement: Supplementary file 1 — Final supplemental document [file 41525_2022_303_MOESM1_ESM.docx]

Supplemental materials

**A statistical perspective on baseline adjustment in pharmacogenomic genome-wide association studies of quantitative change**

Hong Zhang^1^, Aparna Chhibber^2†^, Peter M. Shaw^2^, Devan V. Mehrotra^3^ and Judong Shen^1^*

^1^Biostatistics and Research Decision Sciences, Merck & Co., Inc., Rahway, NJ 07065 USA

^2^Genetics and Biomarker Sciences, Merck & Co., Inc., West Point, PA 19446 USA

^3^Biostatistics and Research Decision Sciences, Merck & Co., Inc., North Wales, PA 19454

^†^Current affiliation: Bristol Myers Squibb, Lawrenceville, NJ, 08540 USA

*email: [judong.shen@merck.com](mailto:judong.shen@merck.com)


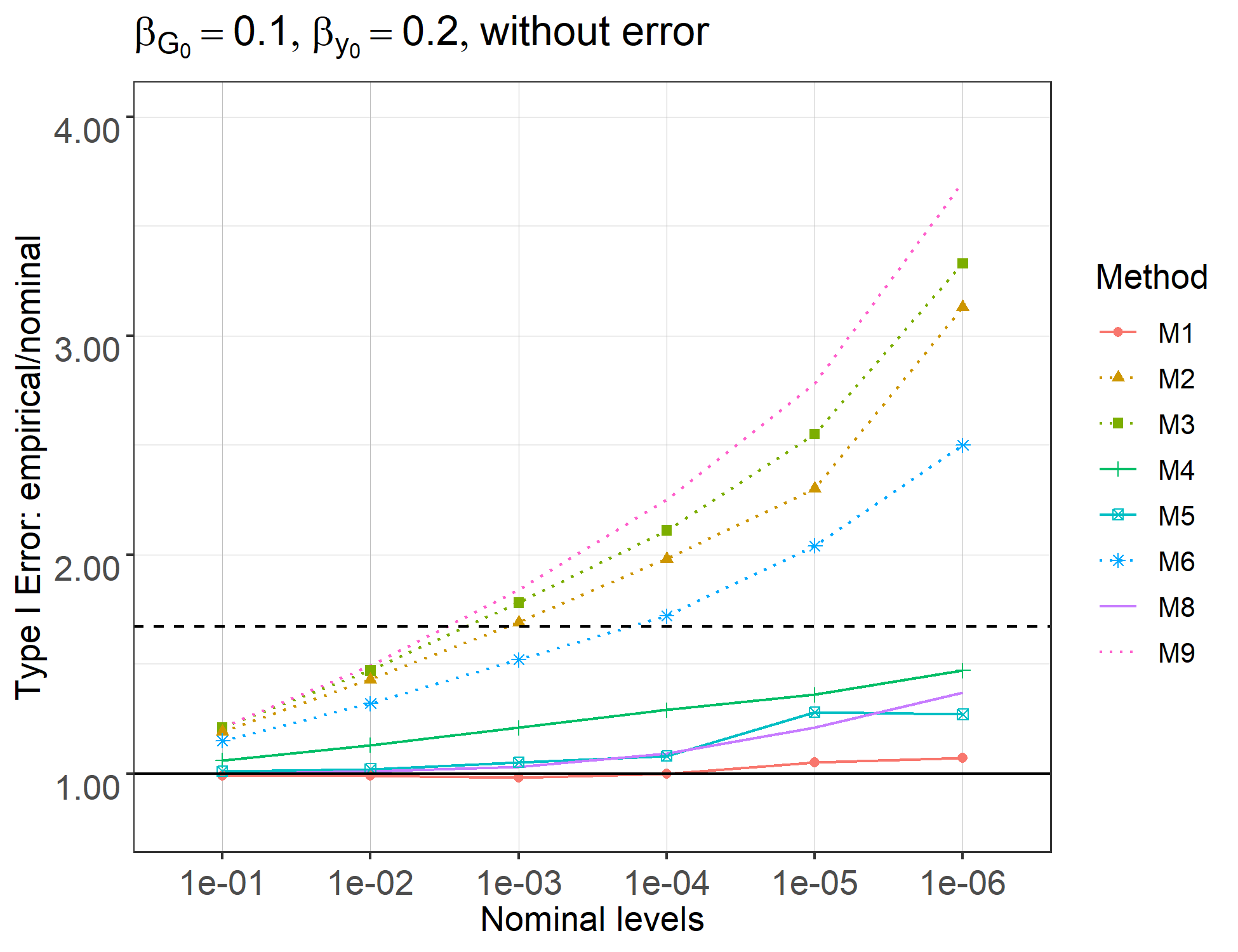

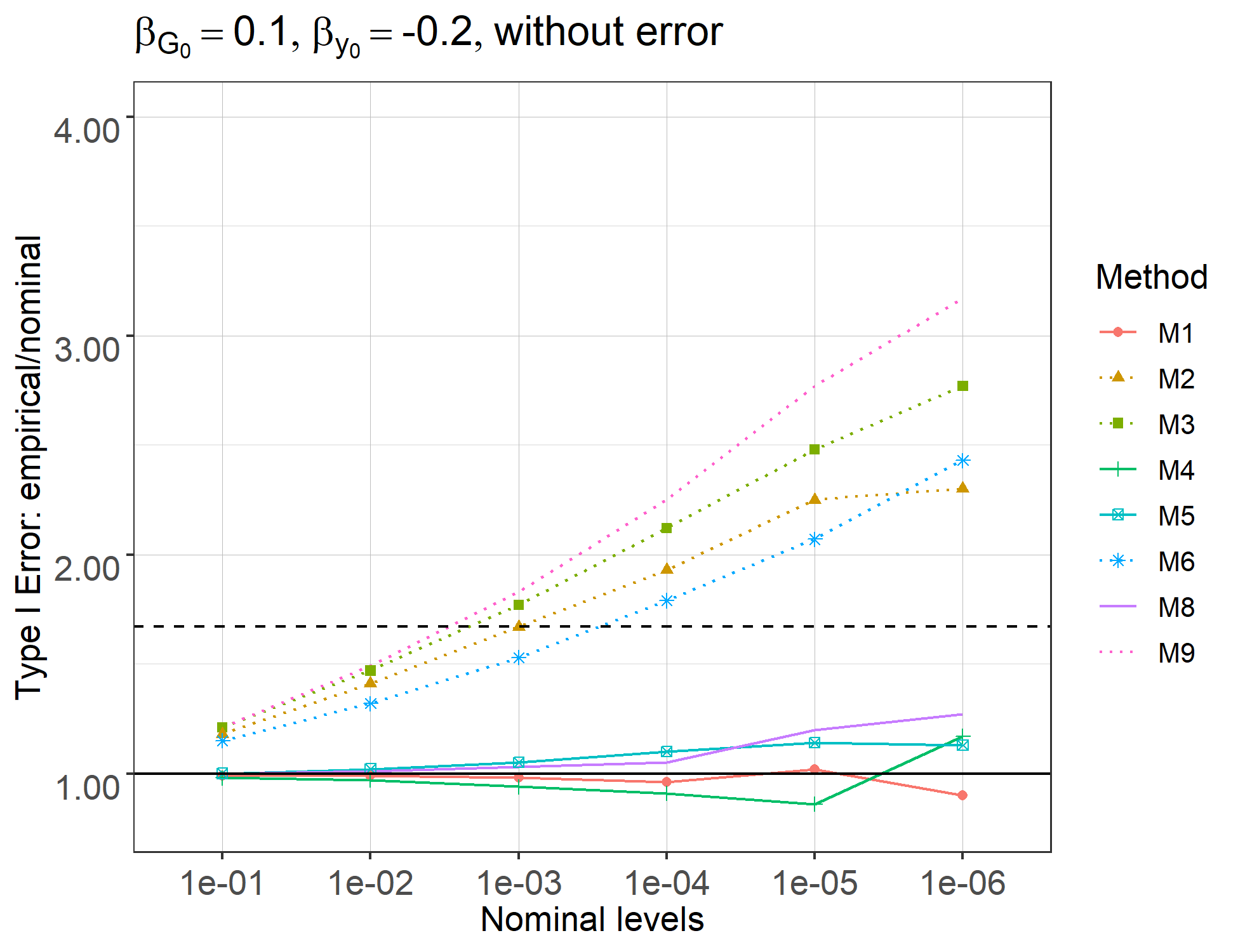

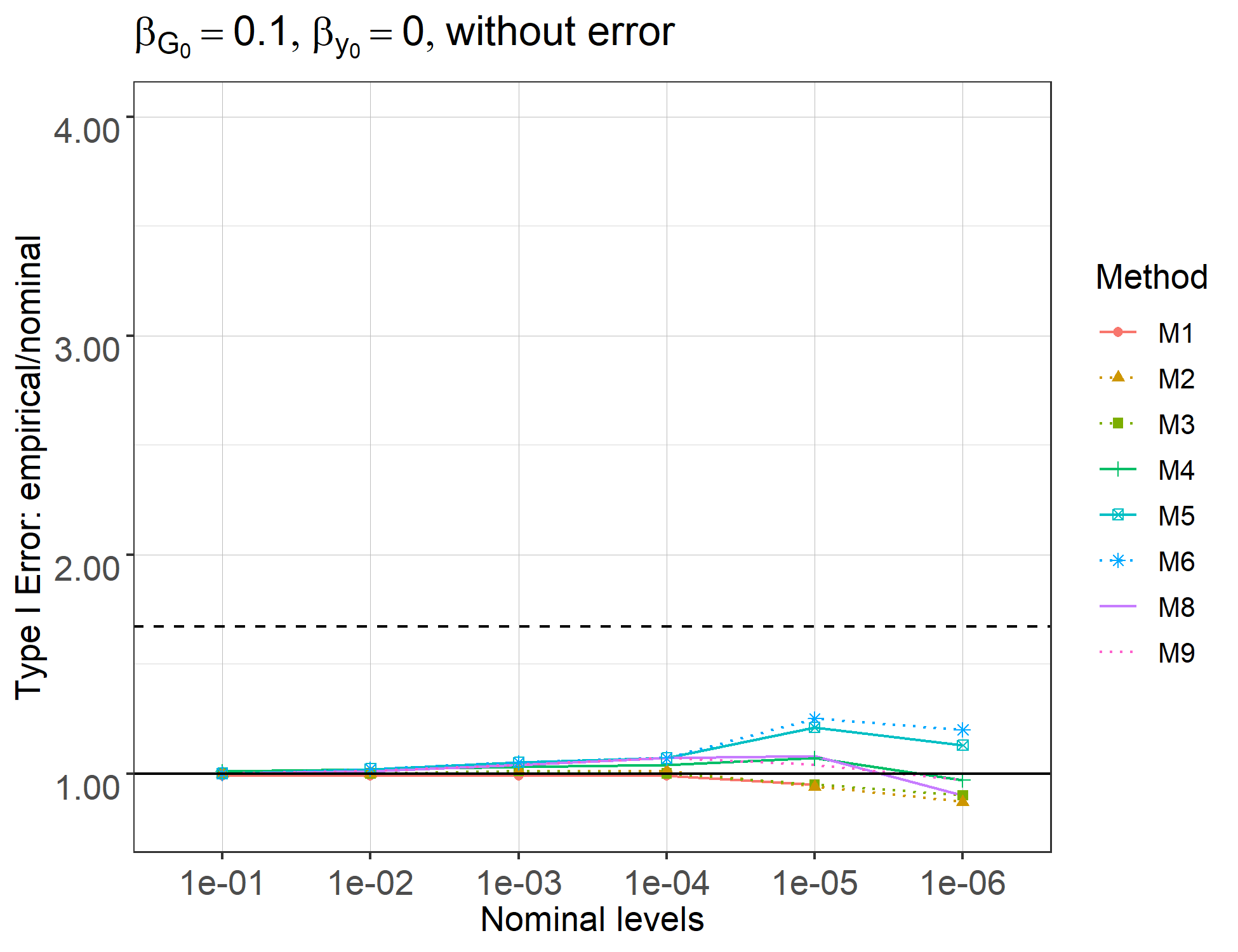

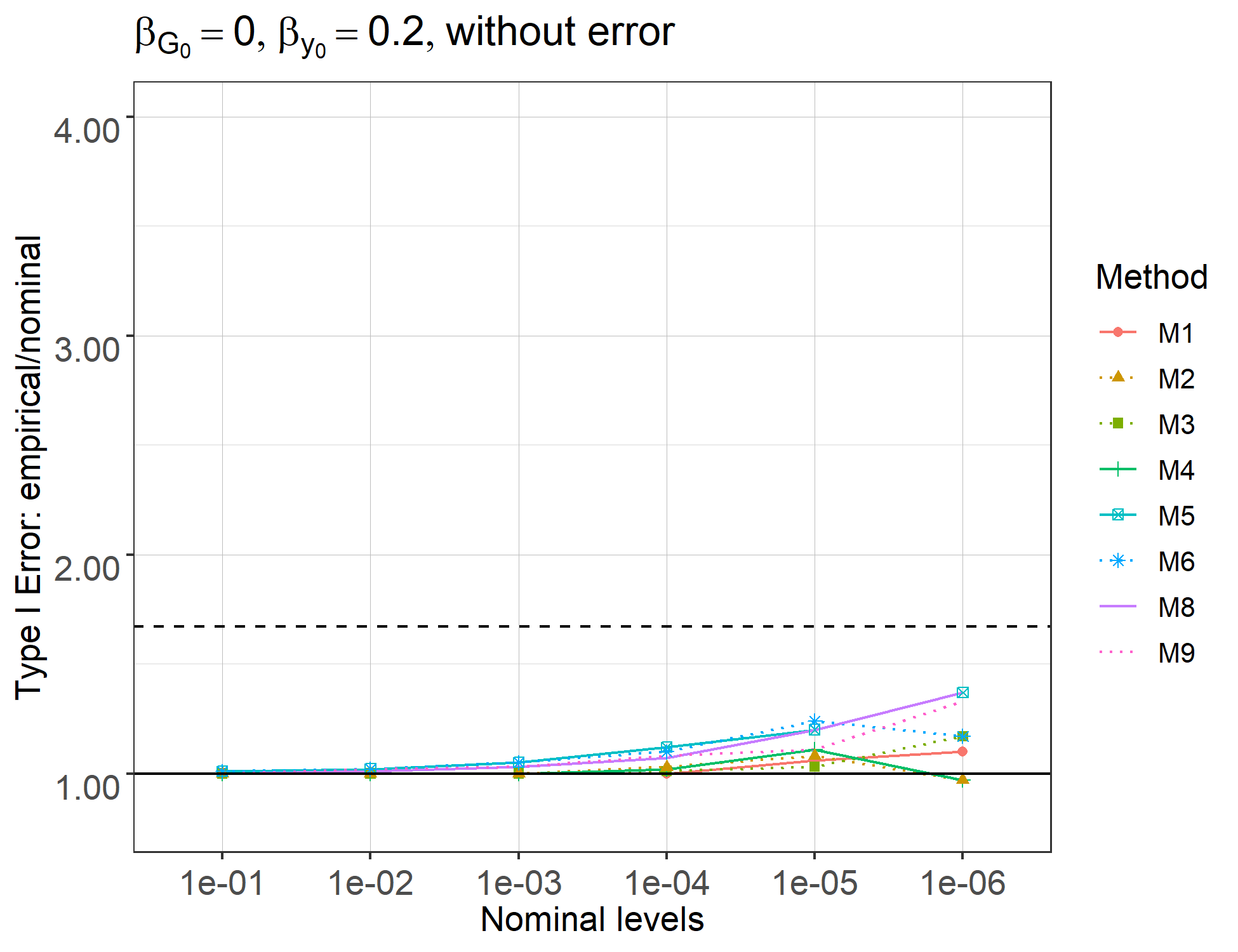

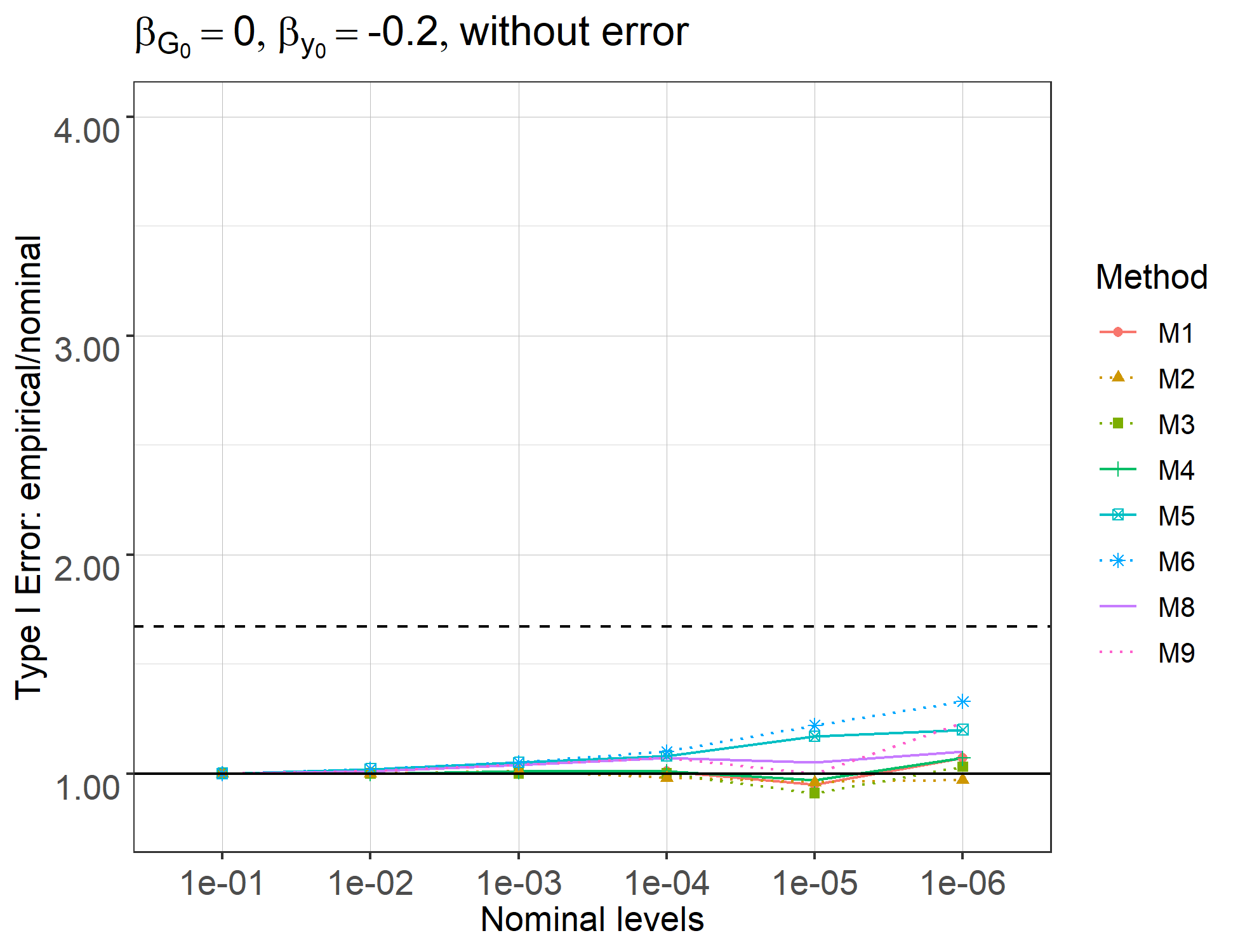

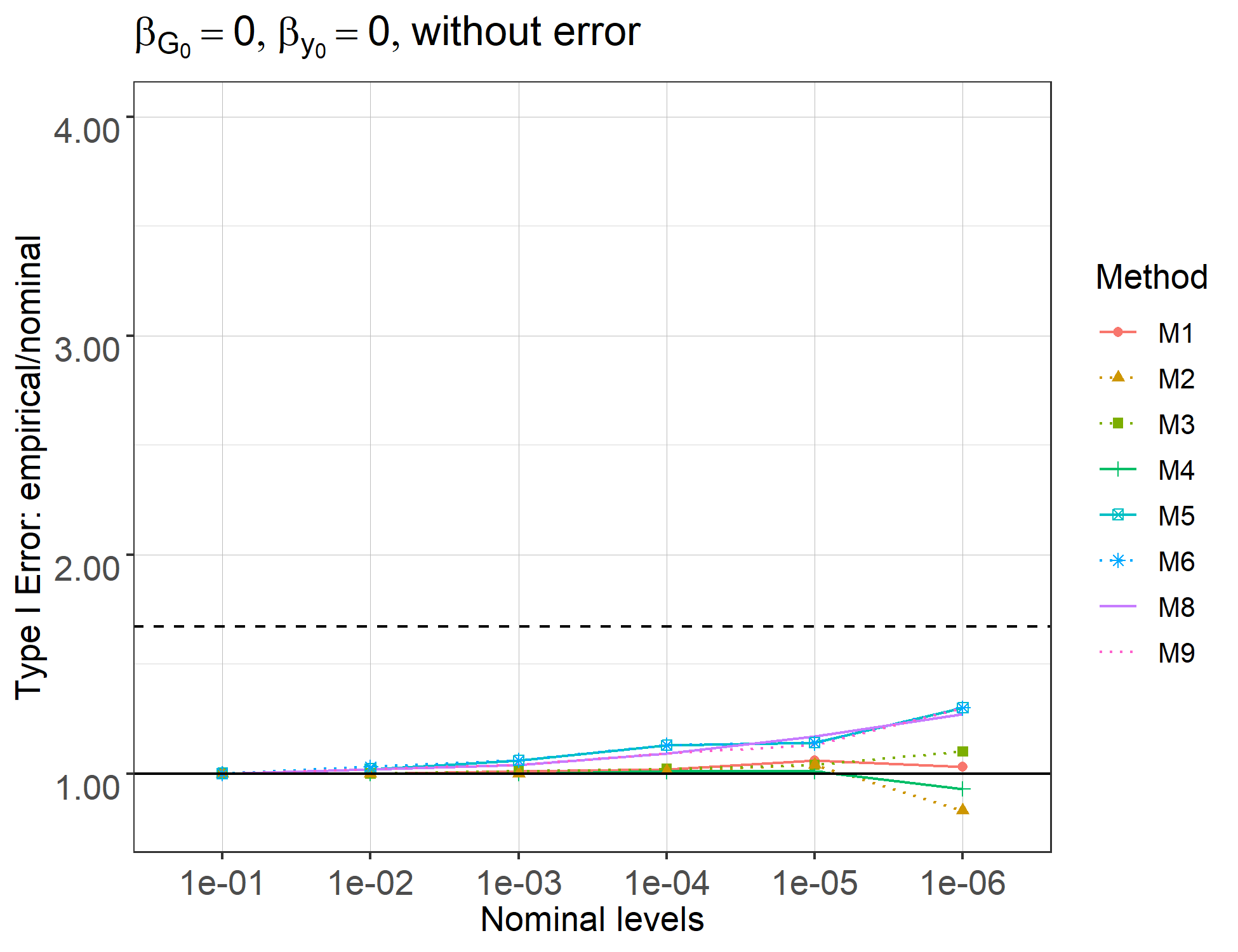

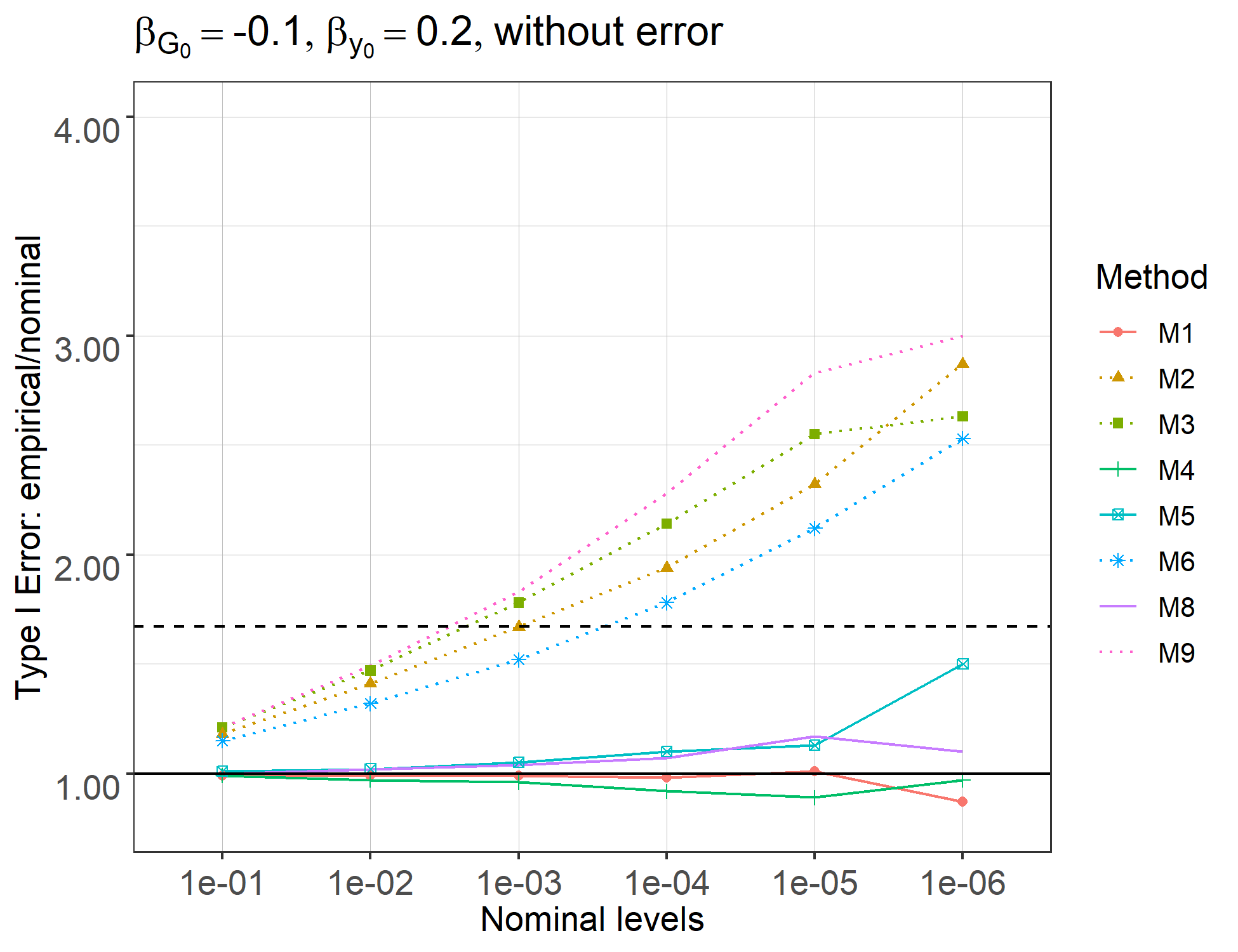

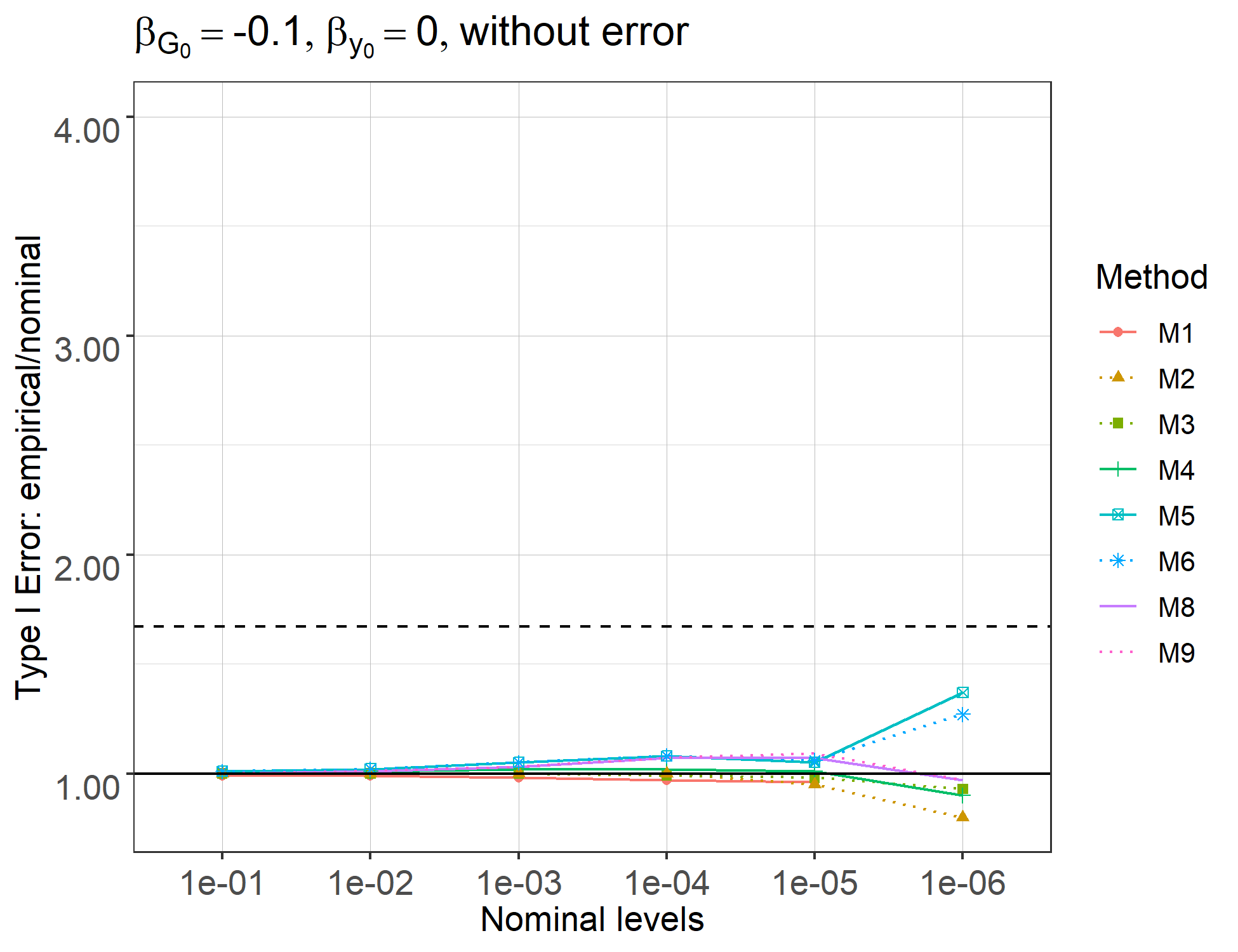

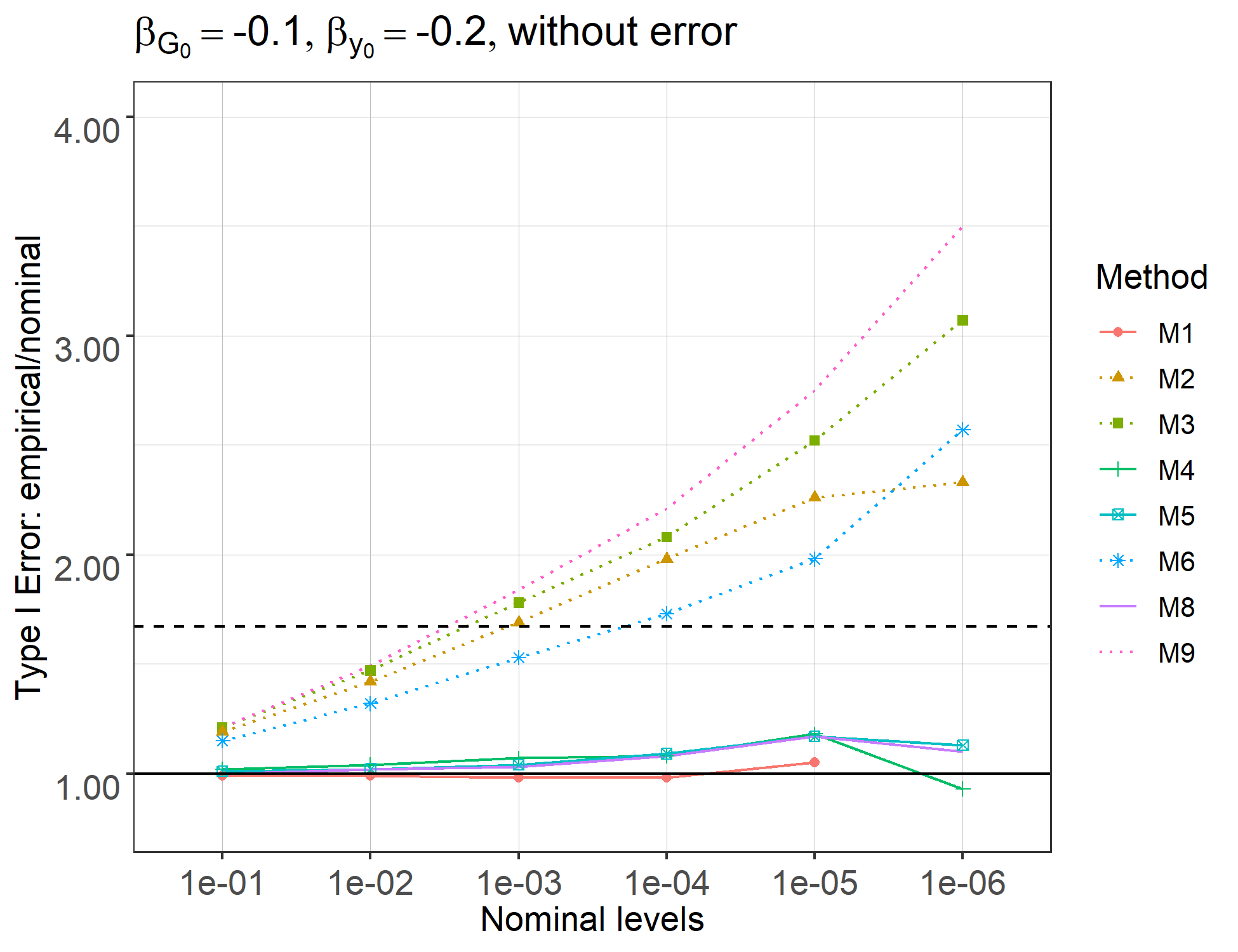


**Supplemental Figure 1**. Ratios between empirical type I error rates and the nominal $\alpha$ levels when measurement errors are absent. The four “corner” panels are the cases when the mediator effect $\beta_{G_{0}}*\beta_{y_{0}}\neq0$. Horizontal dash line: the ratio ($\alpha$ + 3*SE)/$\alpha$ = 1 + 3*SE/$\alpha\approx1.67$, where SE is the margin of error calculated as $\sqrt{\frac{\alpha*(1- \alpha)}{n}}$ , $\alpha={10}^{-6}$ is the nominal level and $n=2\times{10}^{7}$ is the number of simulations. M1-M9 are defined in the Methods section.


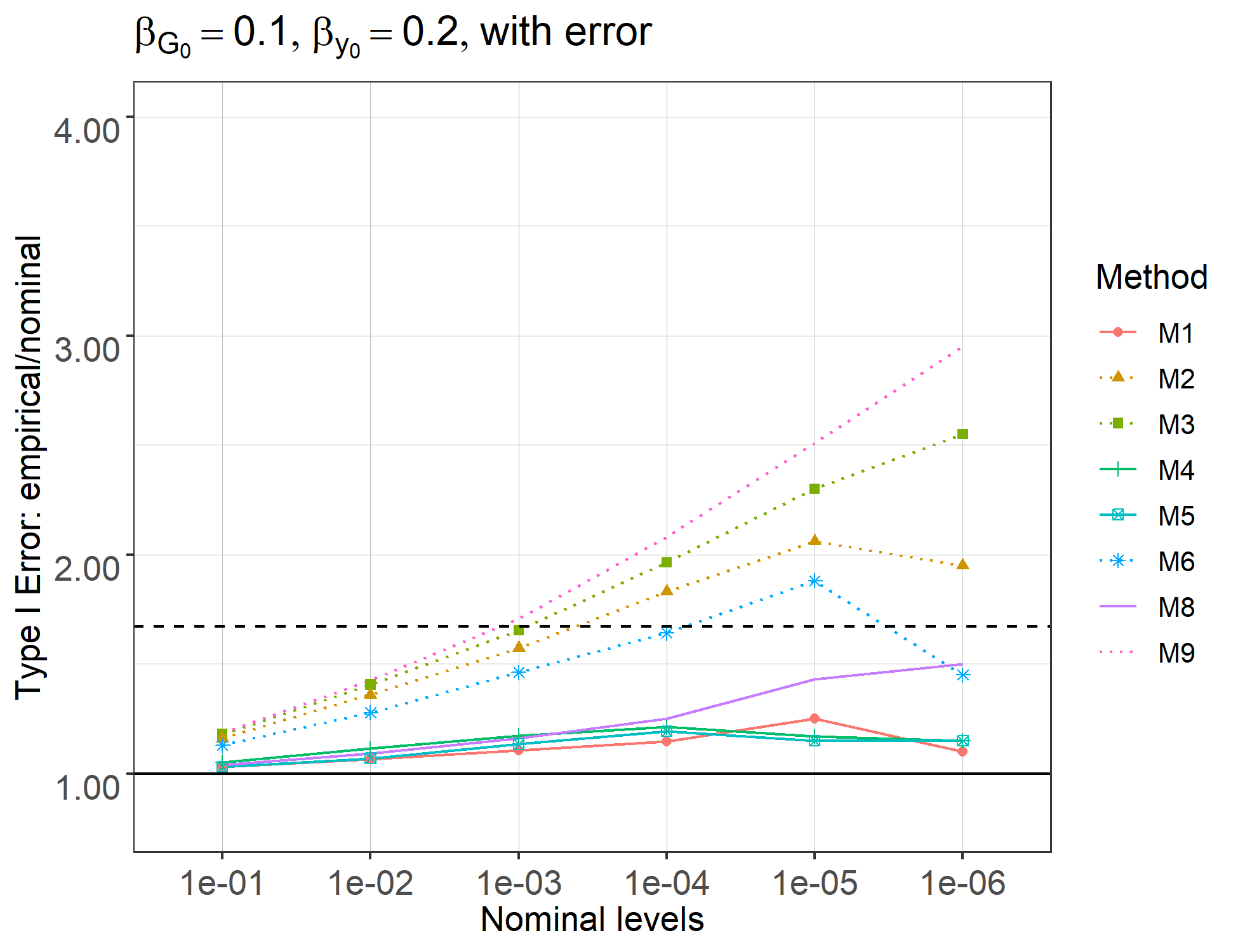

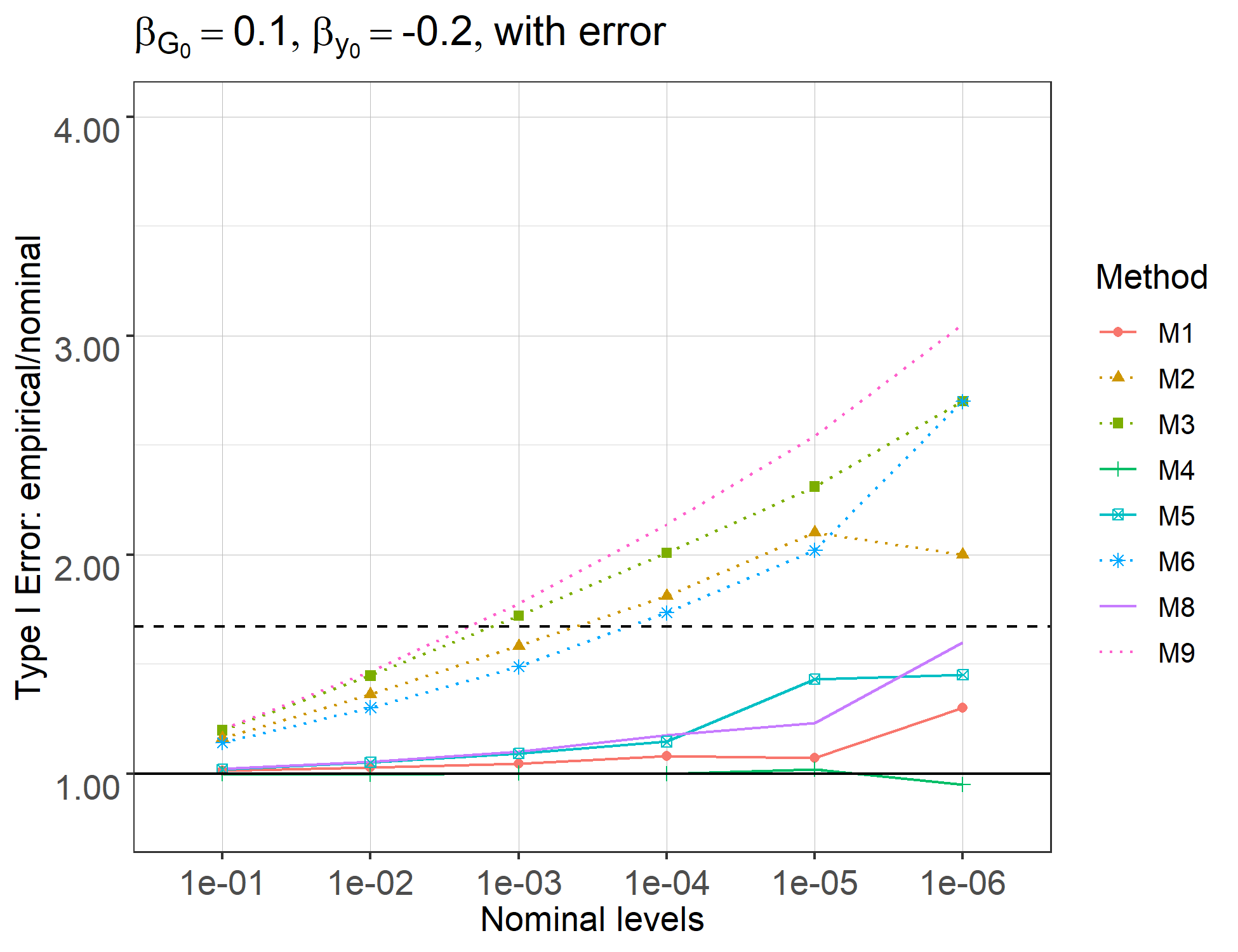

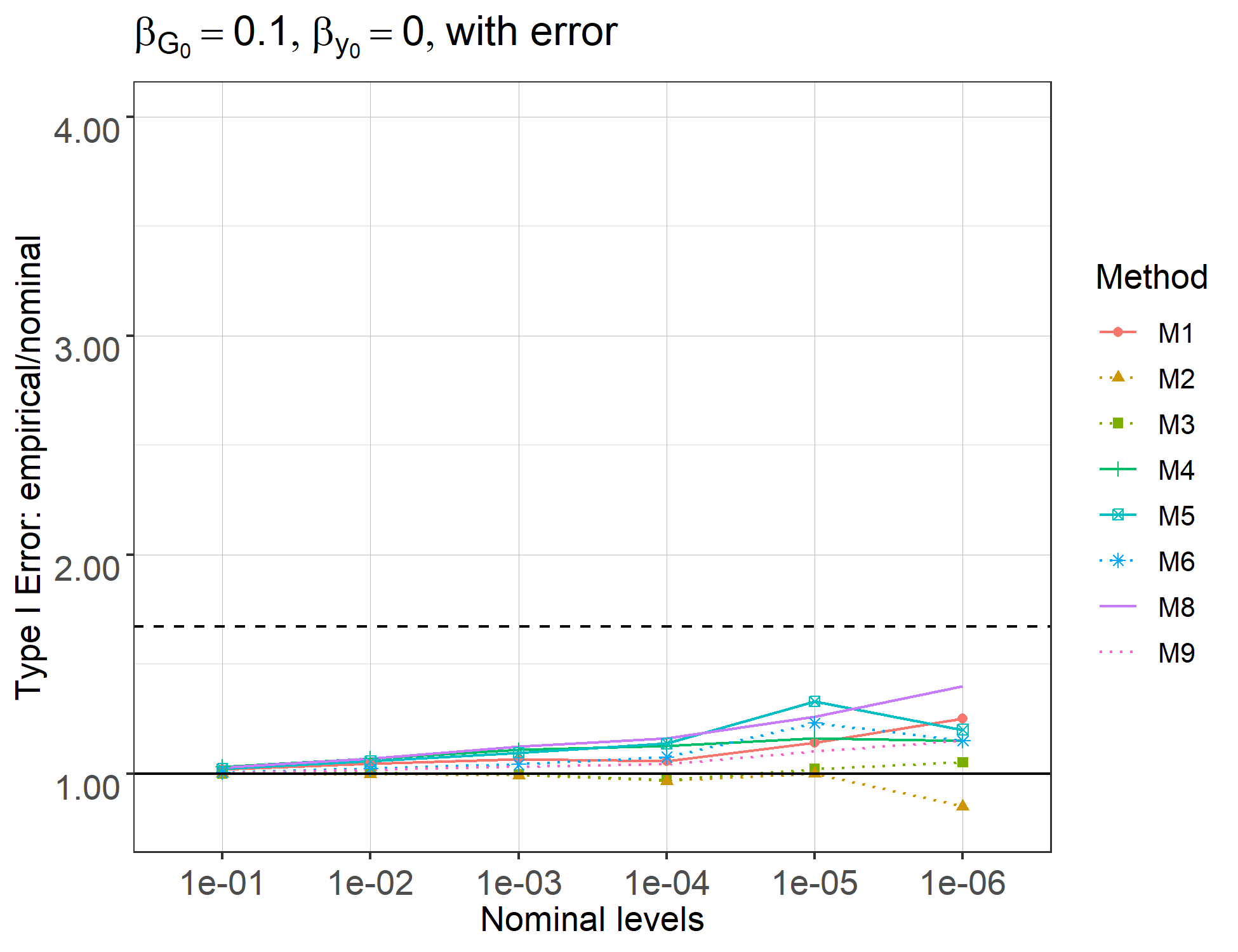

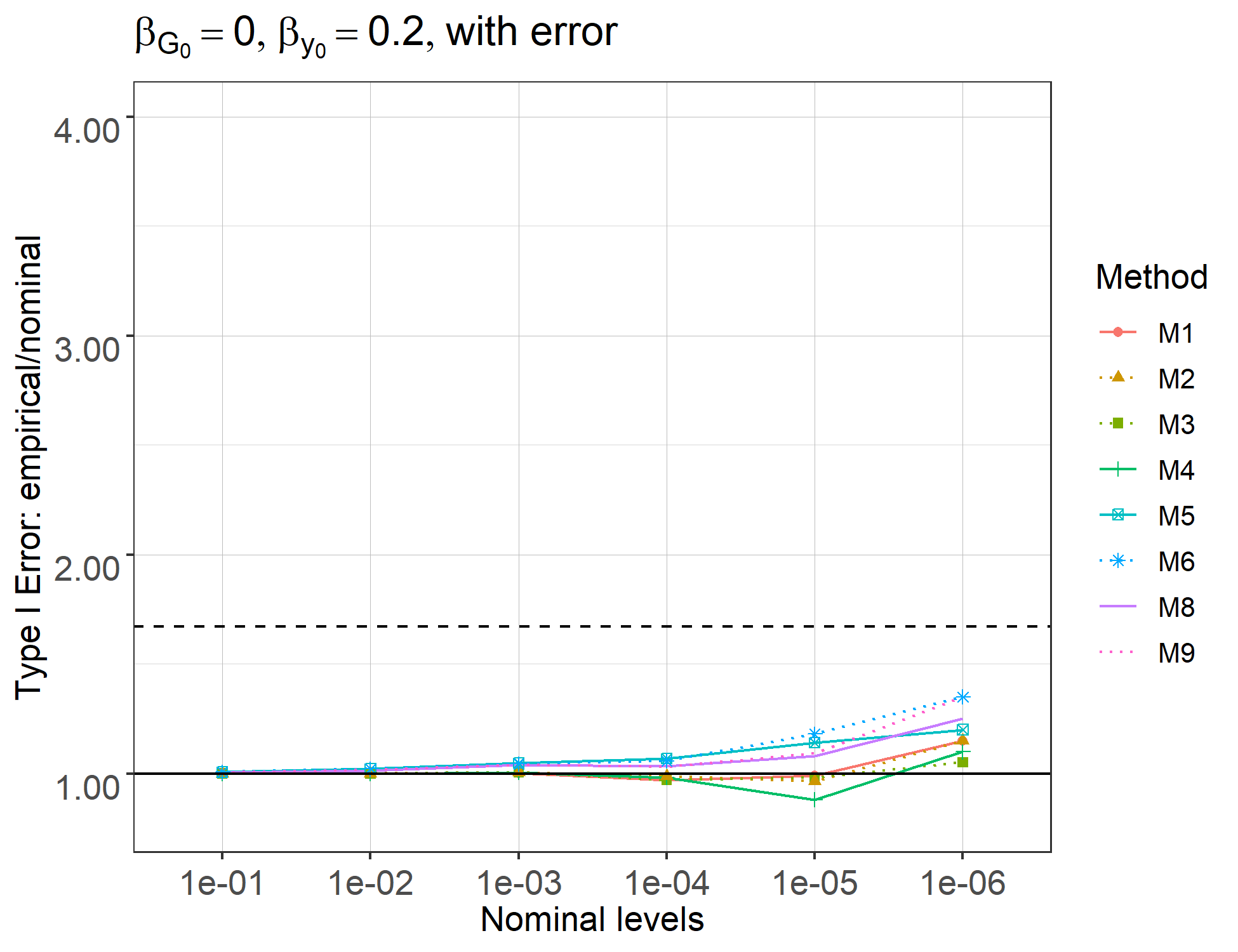

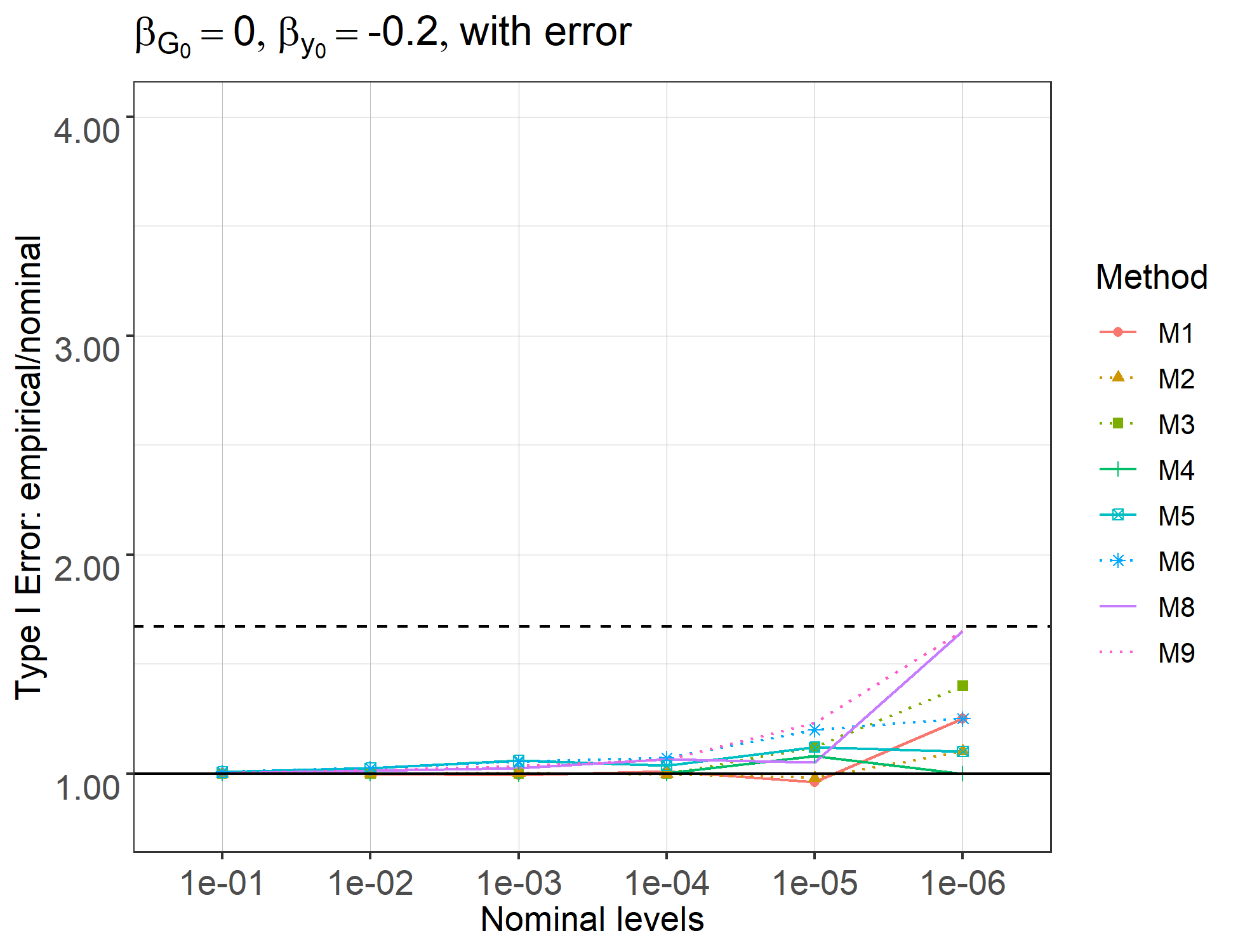

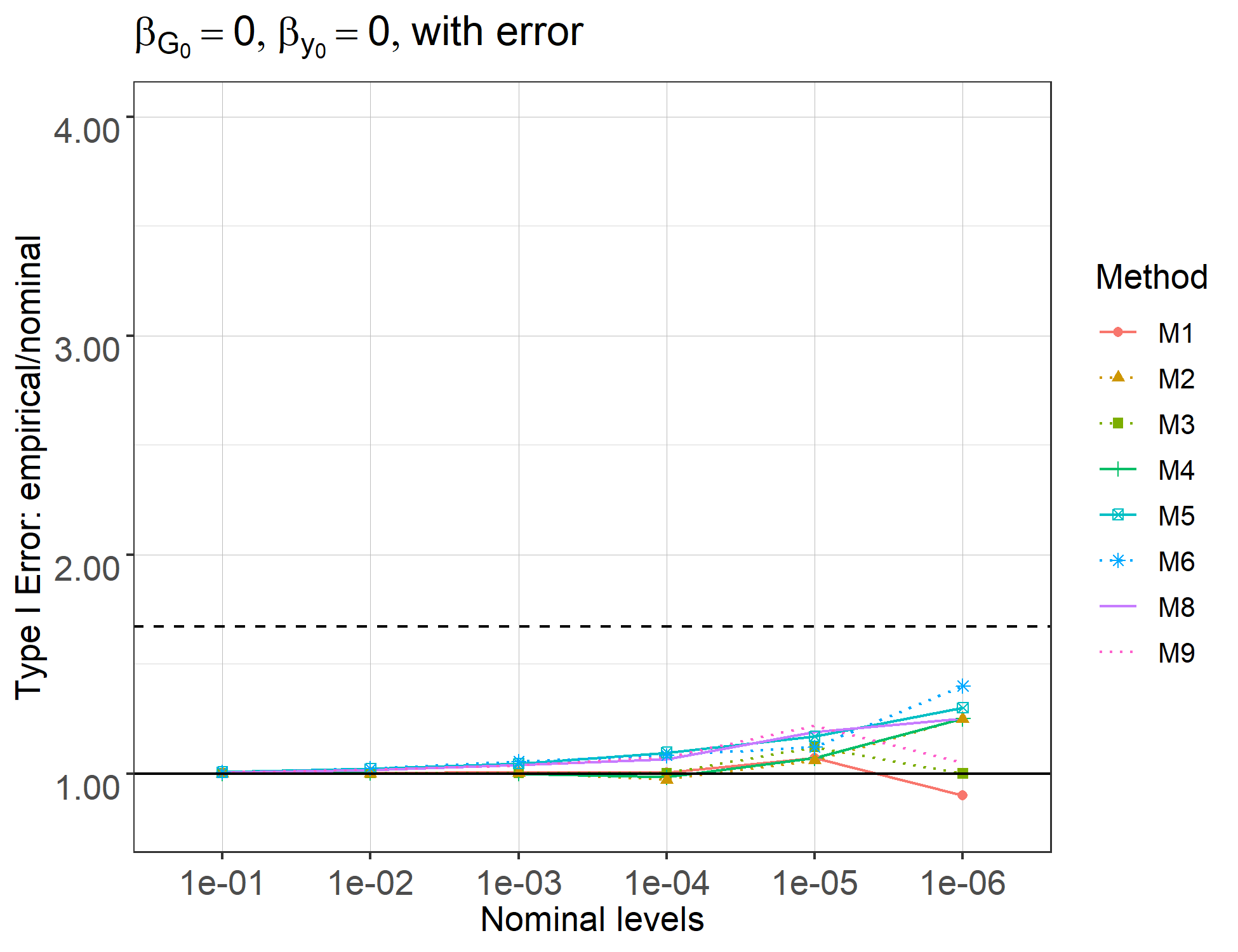

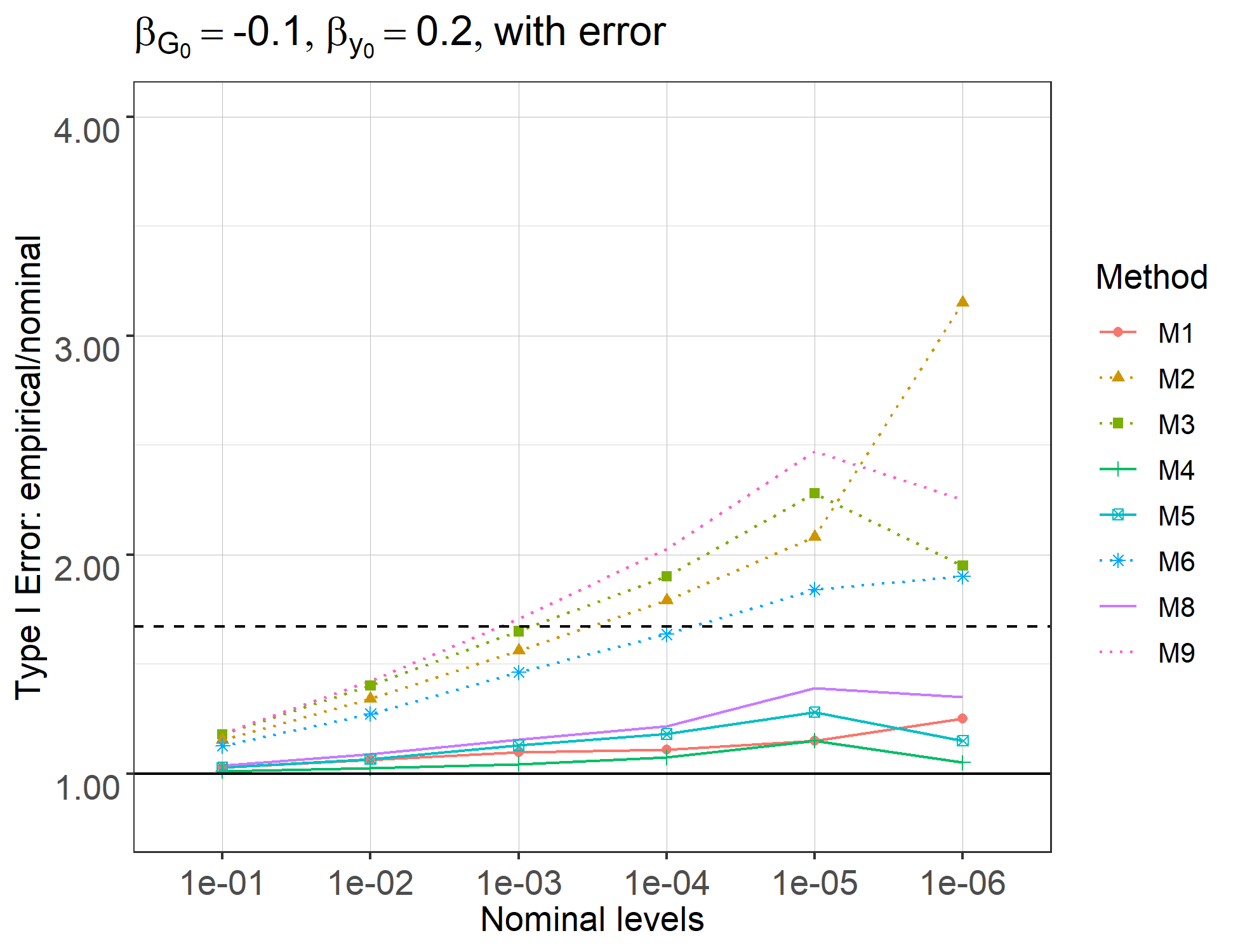

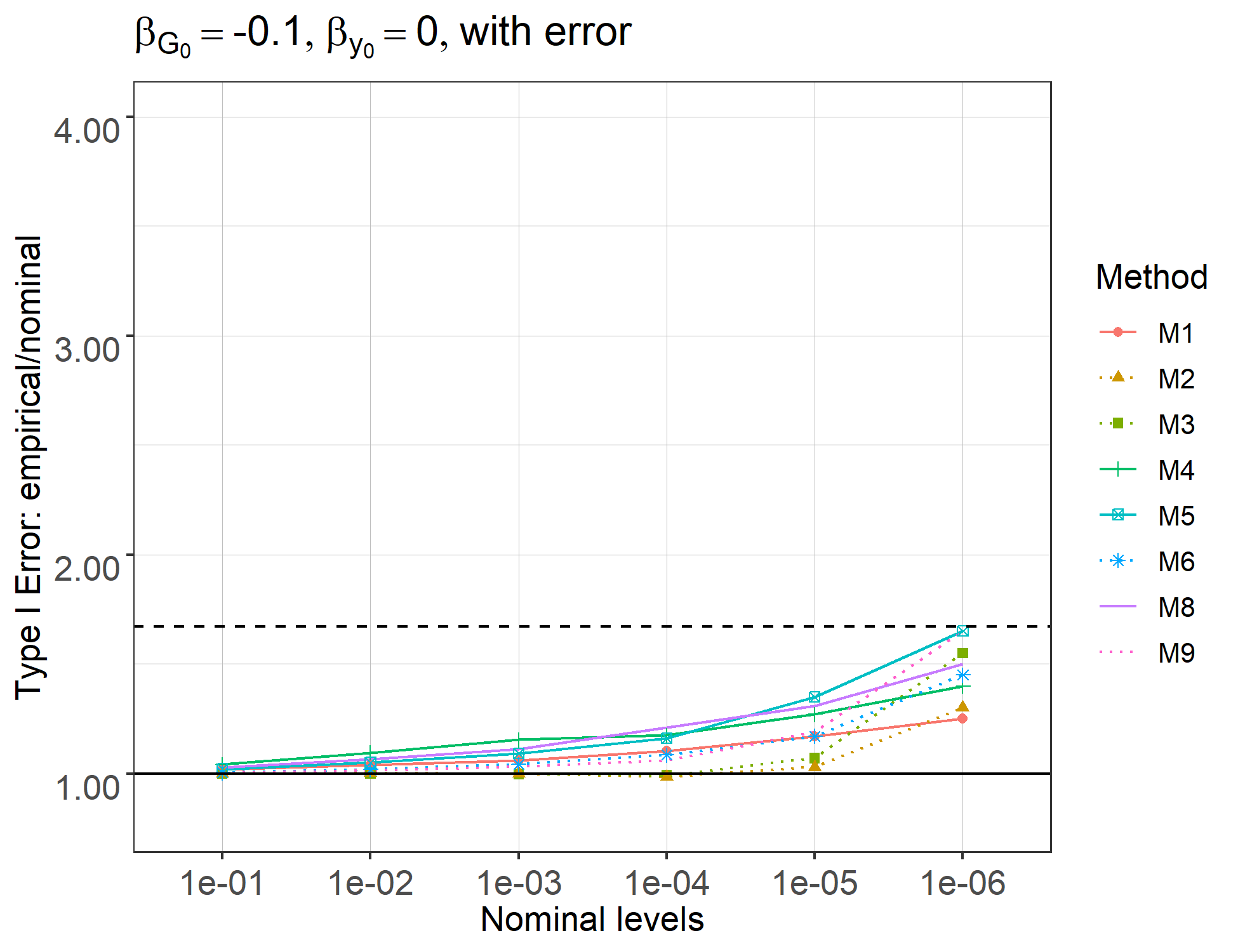

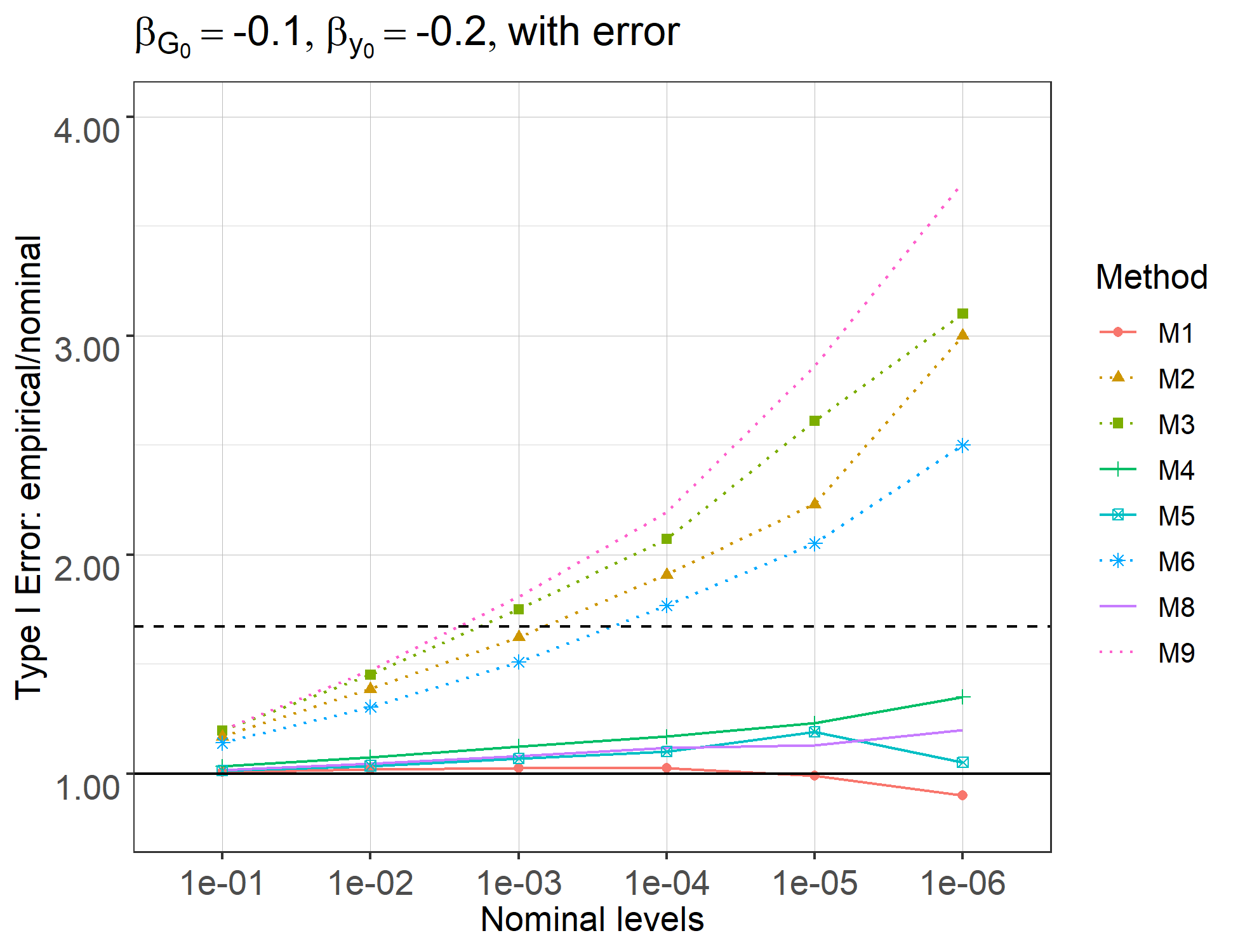


**Supplemental Figure 2**. Ratios between empirical type I error rates and the nominal $\alpha$ levels when measurement errors are present. The four “corner” panels are the cases when the mediator effect $\beta_{G_{0}}*\beta_{y_{0}}\neq0$. Horizontal dash line: the ratio ($\alpha$ + 3*SE)/$\alpha$ = 1 + 3*SE/$\alpha\approx1.67$, where SE is the margin of error calculated as $\sqrt{\frac{\alpha*(1- \alpha)}{n}}$, $\alpha={10}^{-6}$ is the nominal level and $n=2\times{10}^{7}$ is the number of simulations. M1-M9 are defined in the Methods section.


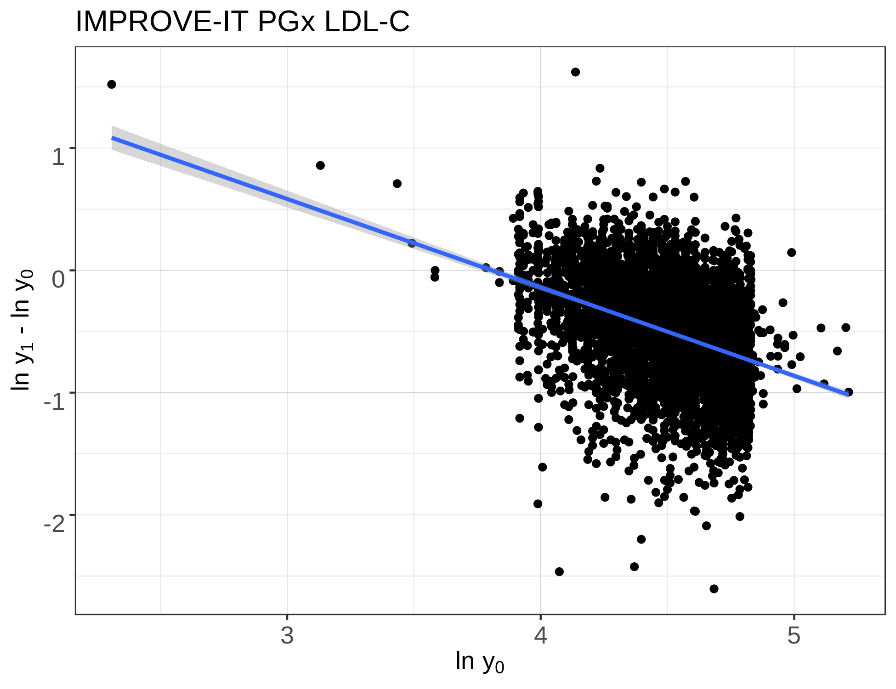


**Supplemental Figure 3**. The scatter plot of nature log transformed baseline LDL-C and nature log transformed change from baseline of LDL-C in the PGx population (N = 5,661) of the IMPROVE-IT clinical trial.


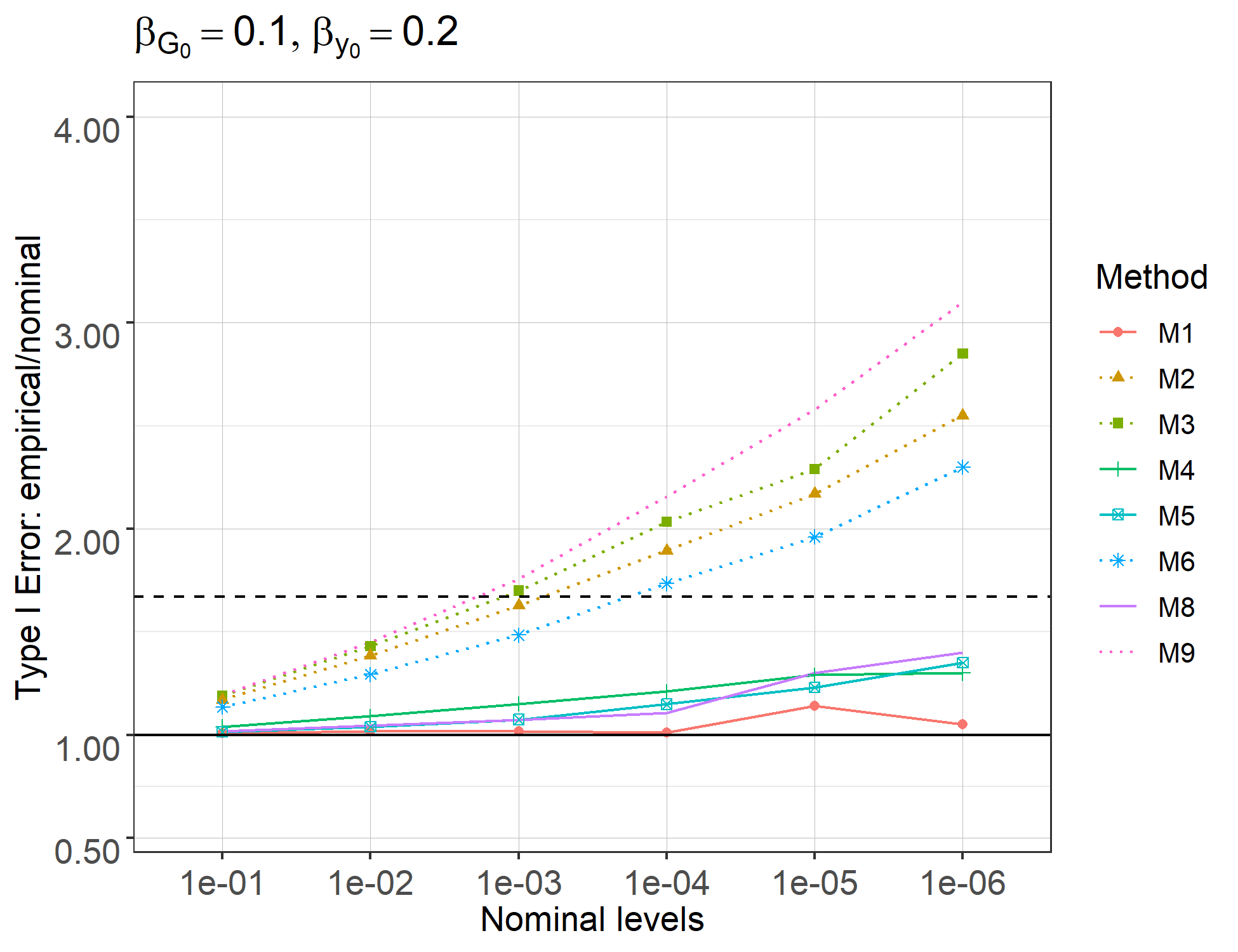

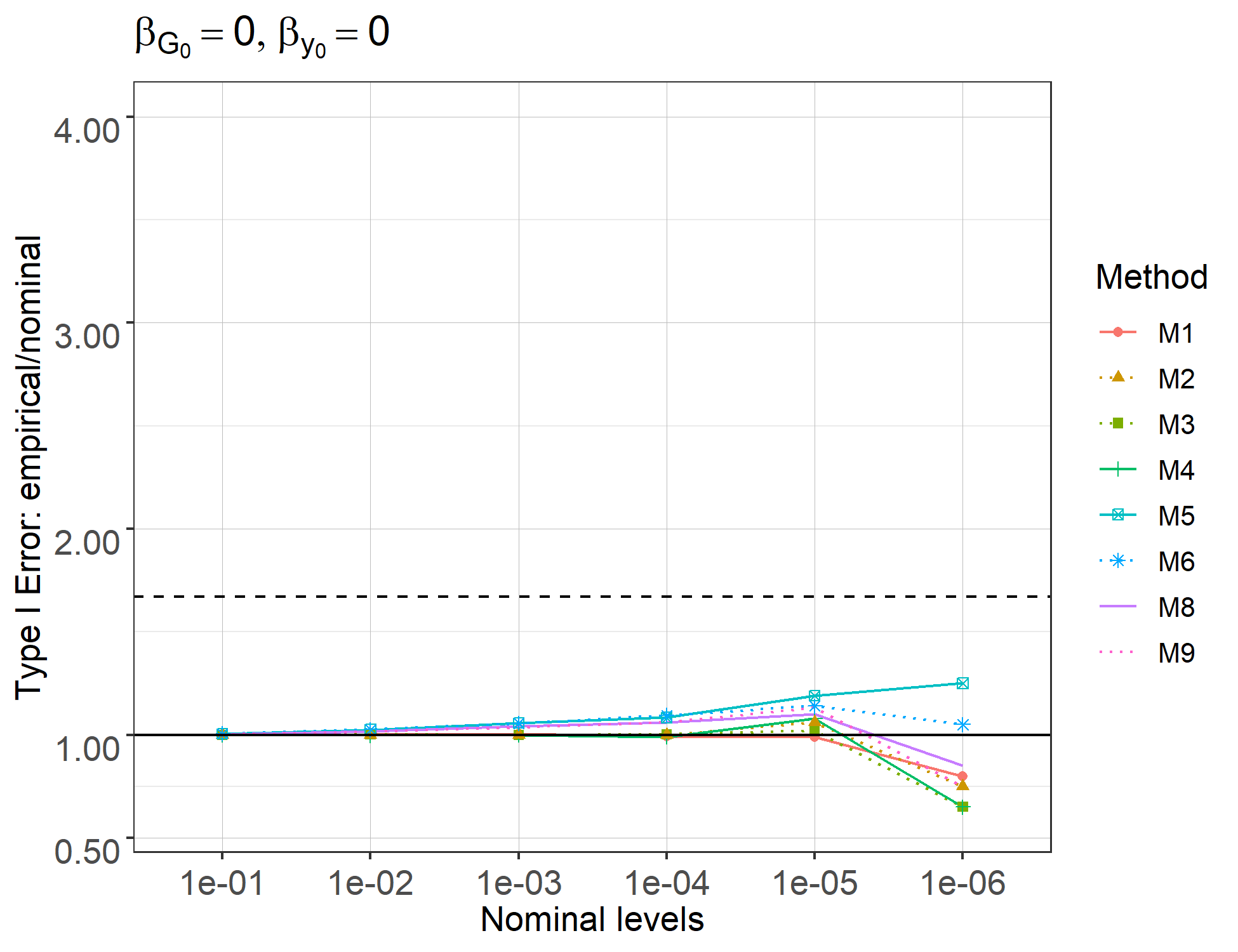

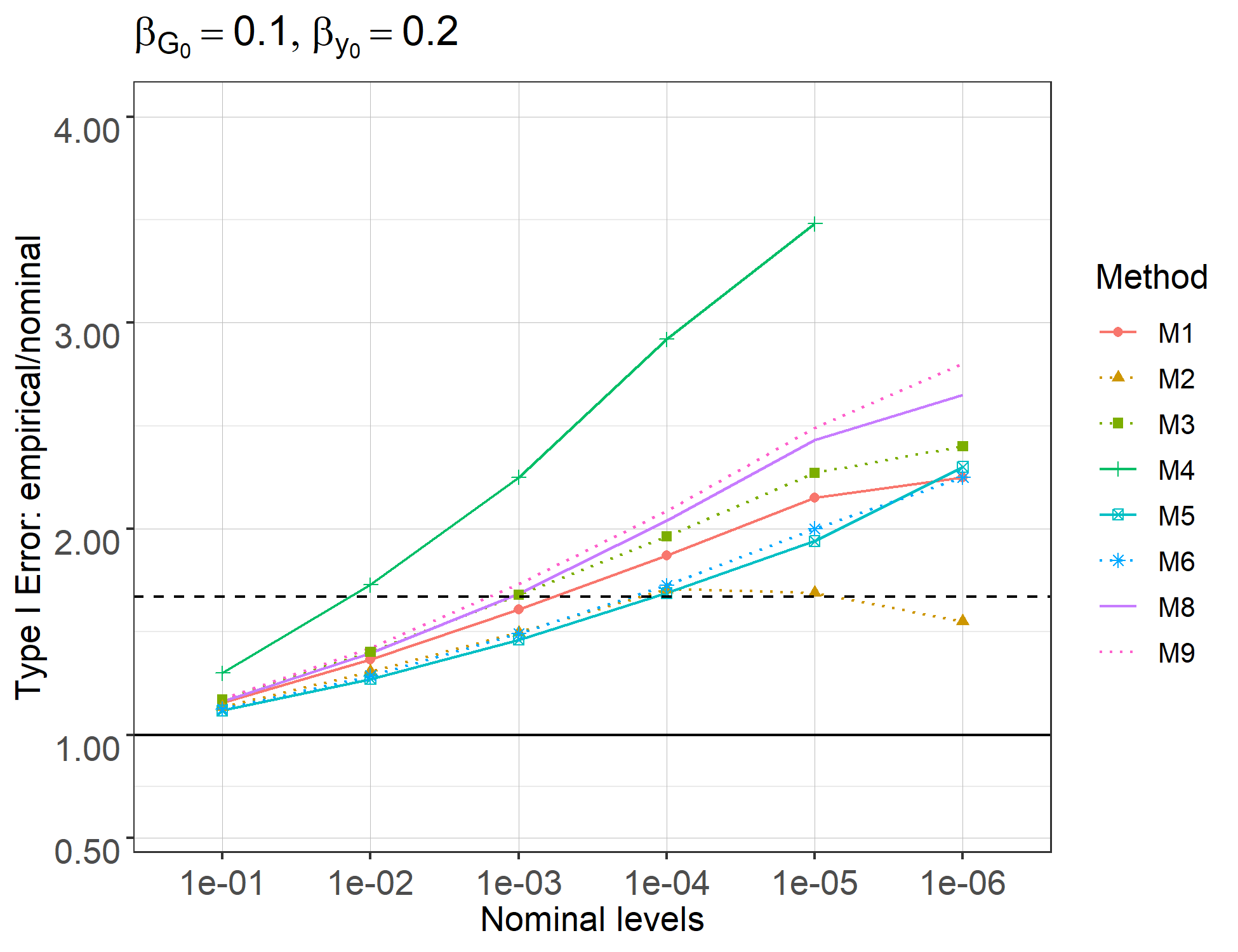

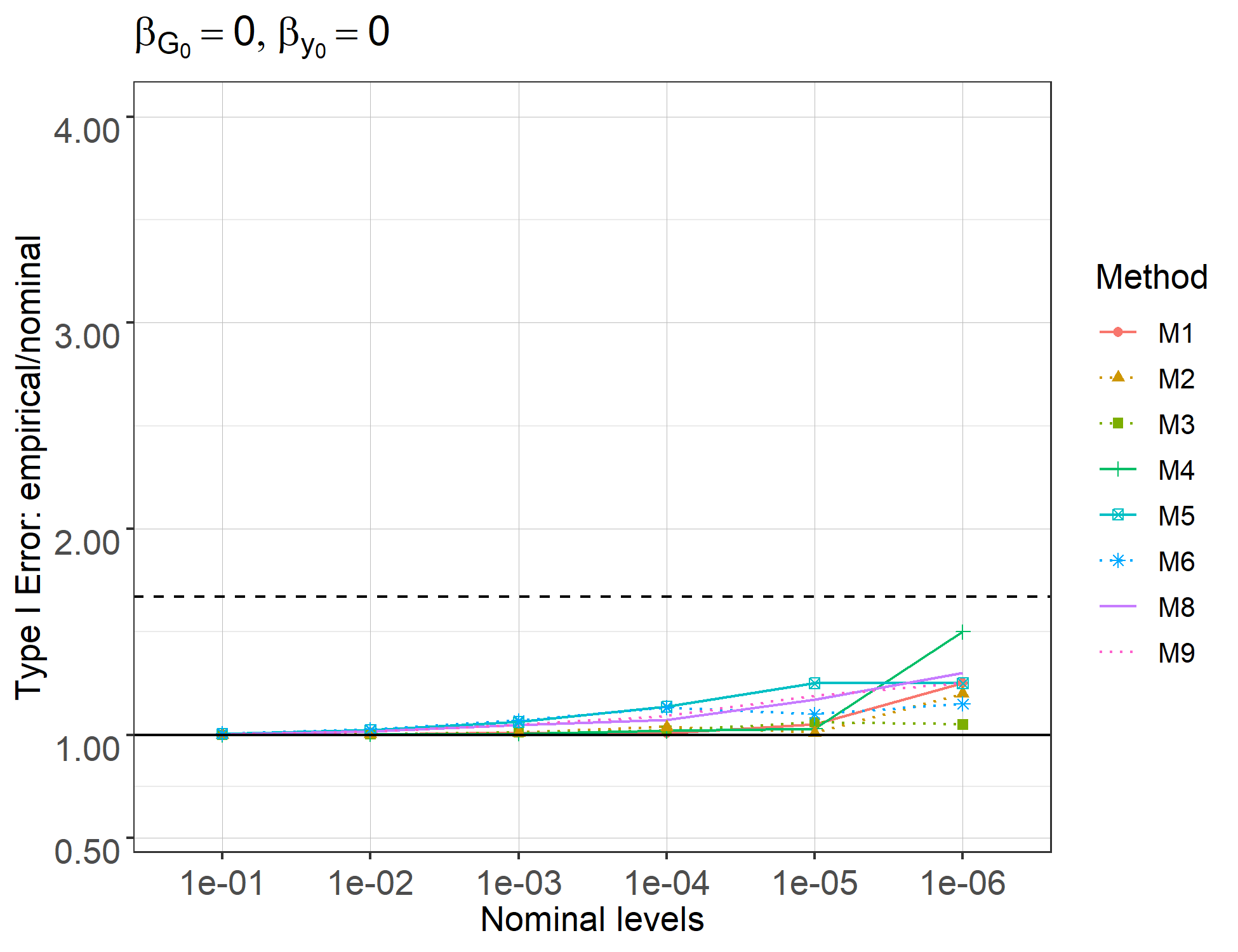


, with error config. ii

, with error config. ii

, with error config. iii

, with error config. iii

**Supplemental Figure 4**. Ratios between empirical type I error rates and the nominal $\alpha$ levels when measurement errors are present. Relative error rates $r_{i} \sim N\left( \mu_{i},\sigma=\frac{1}{4} \right), i=0, 1$. Configuration ii: $\mu_{i}\equiv0.25$. Configuration iii: $\mu_{0}=-0.25, \mu_{1}=0.25$. Inflation > 4 is not shown in the graph. Horizontal dash line: the ratio ($\alpha$ + 3*SE)/$\alpha$ = 1 + 3*SE/$\alpha\approx1.67$, where EE is the standard error calculated as $\sqrt{\frac{\alpha*(1- \alpha)}{n}}$, $\alpha={10}^{-6}$ is the nominal level and $n=2\times{10}^{7}$ is the number of simulations. M1-M9 are defined in the Methods section.


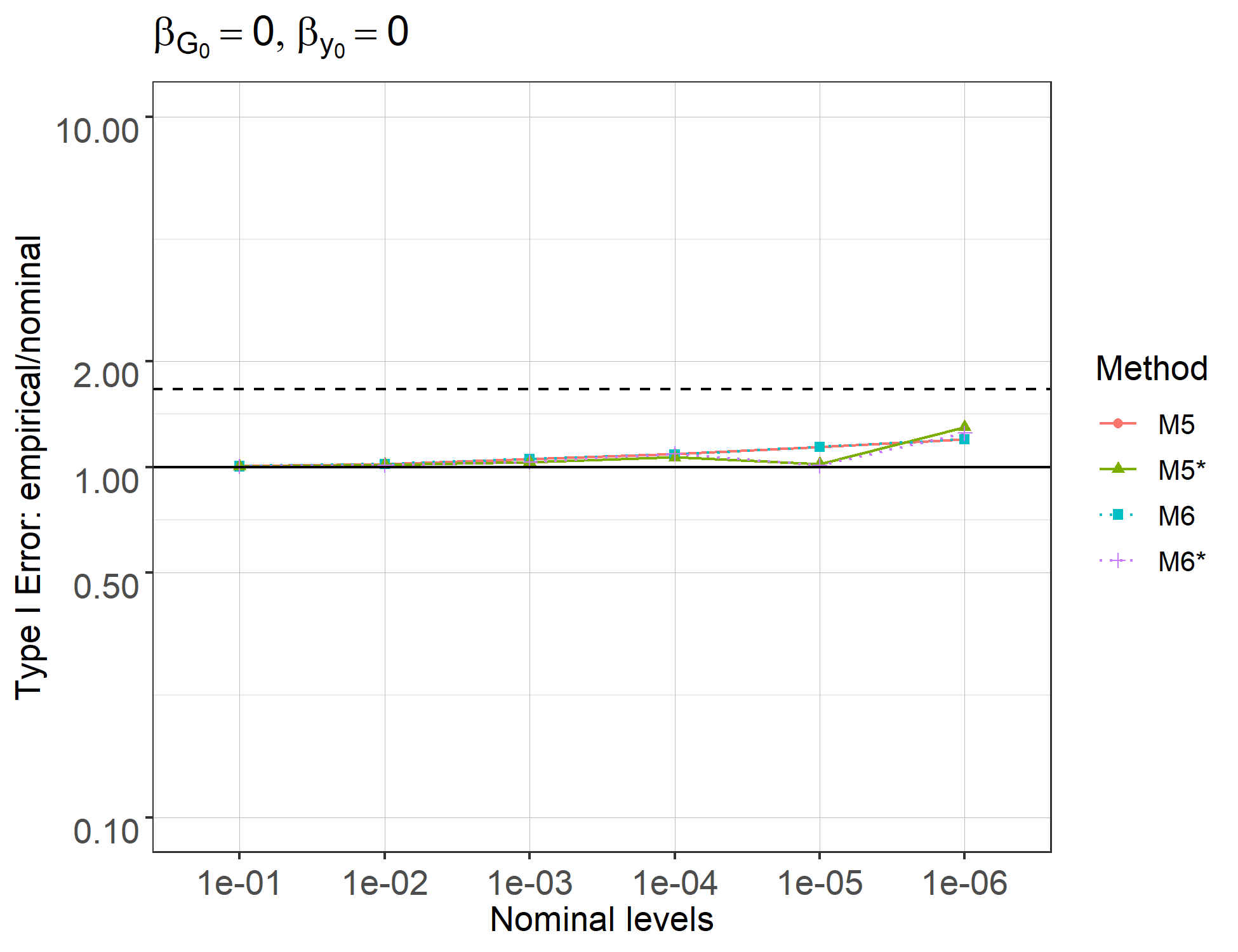

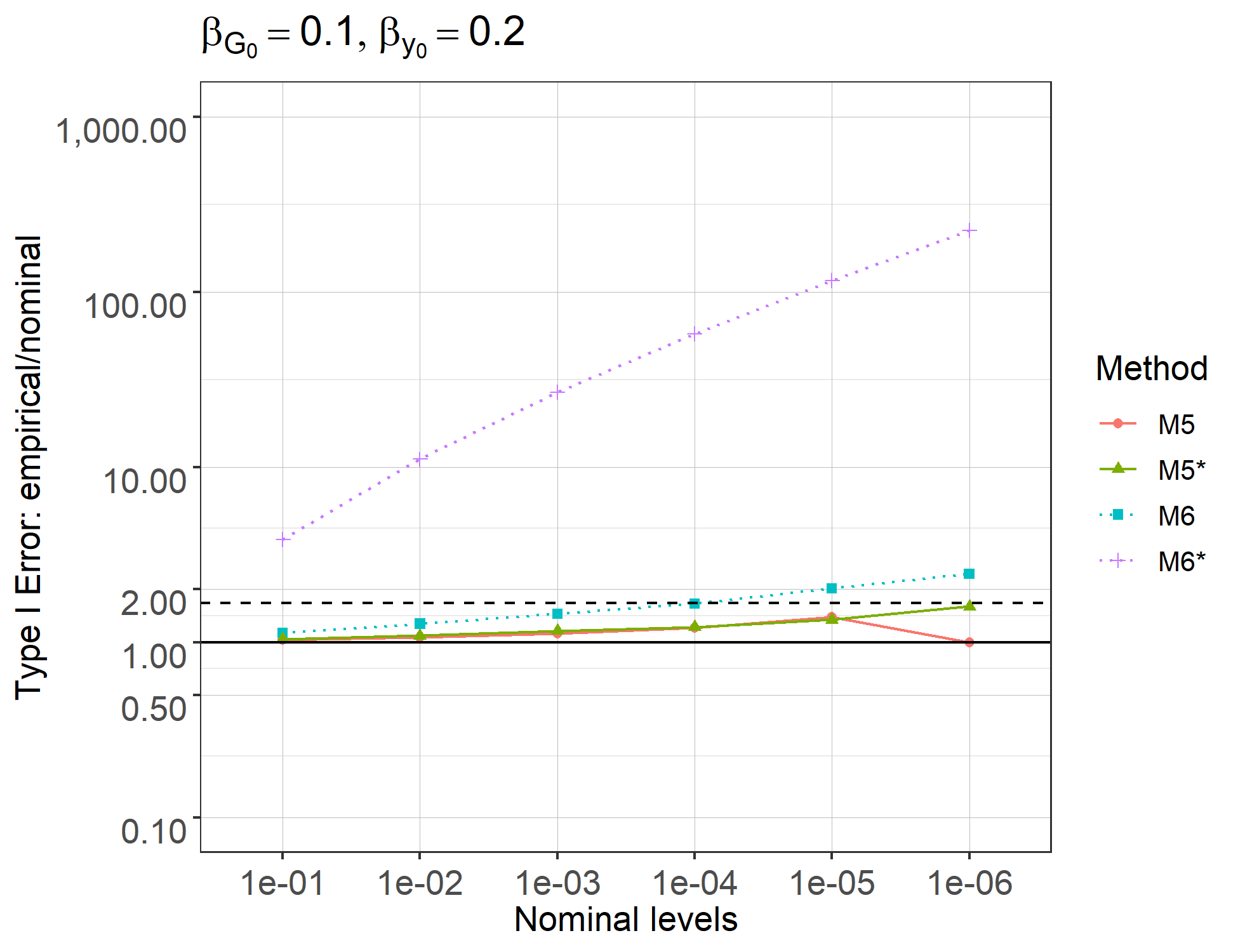


, with error config. i

, with error config. i

, with error config. ii

, with error config. ii

, with error config. iii

, with error config. iii


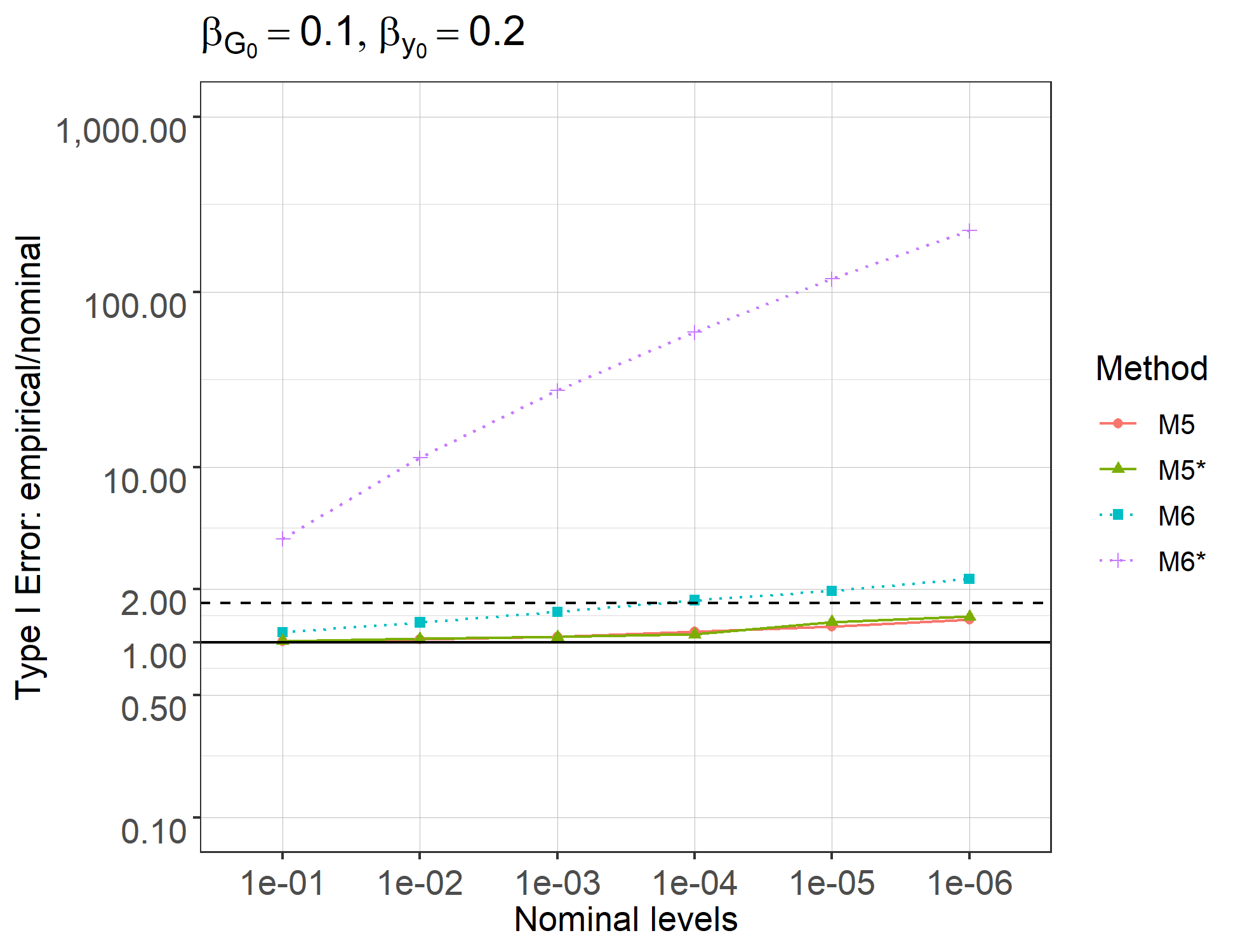

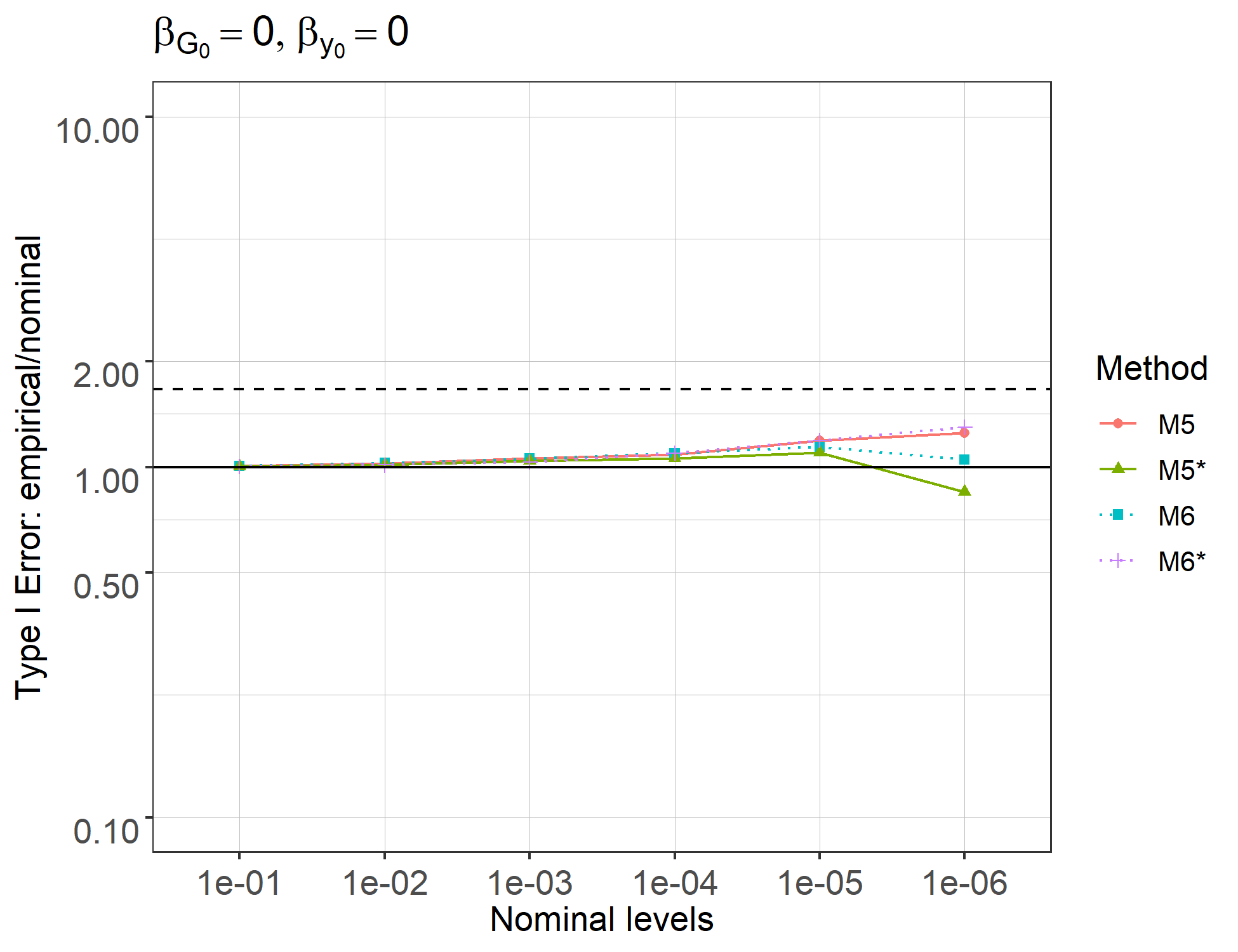

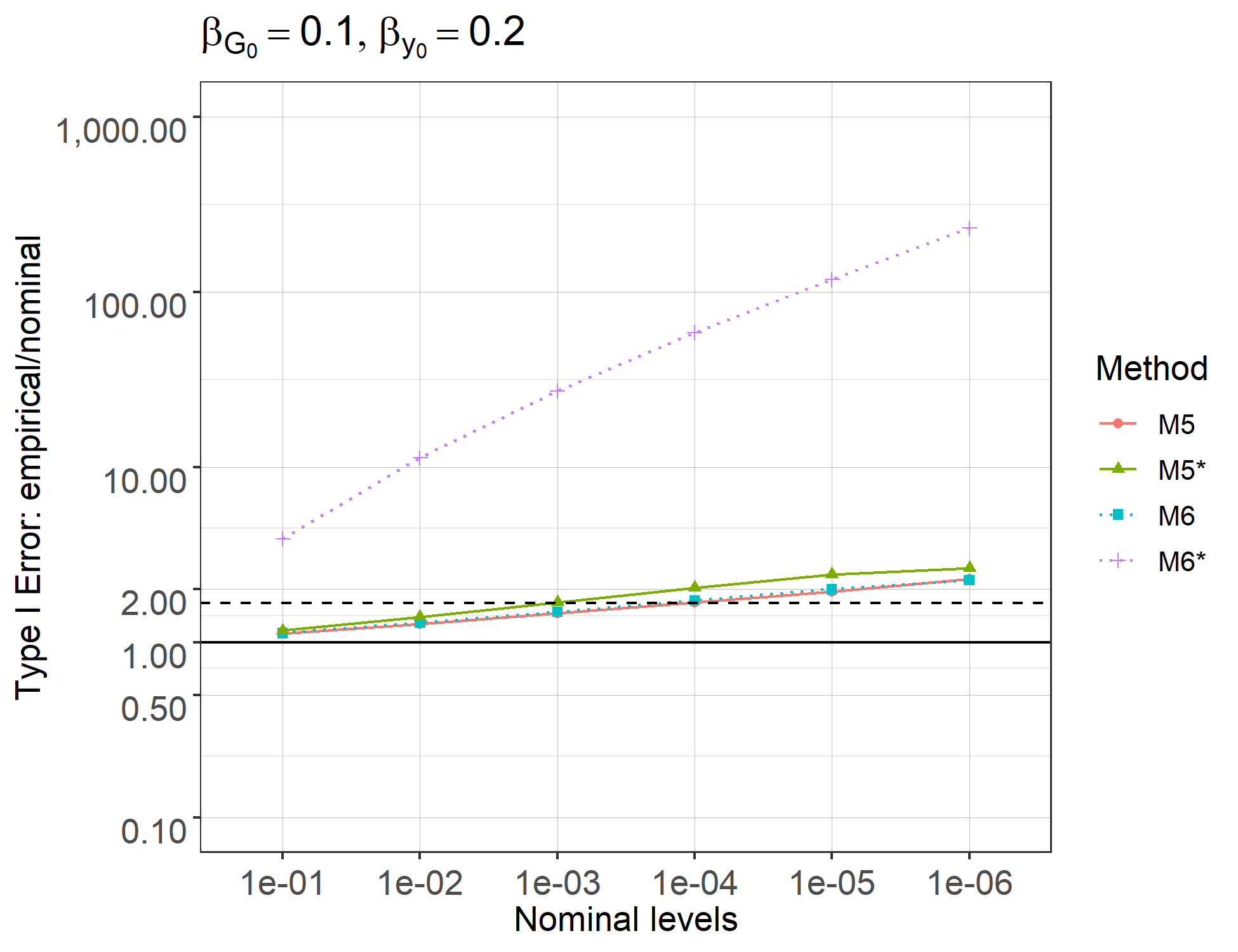

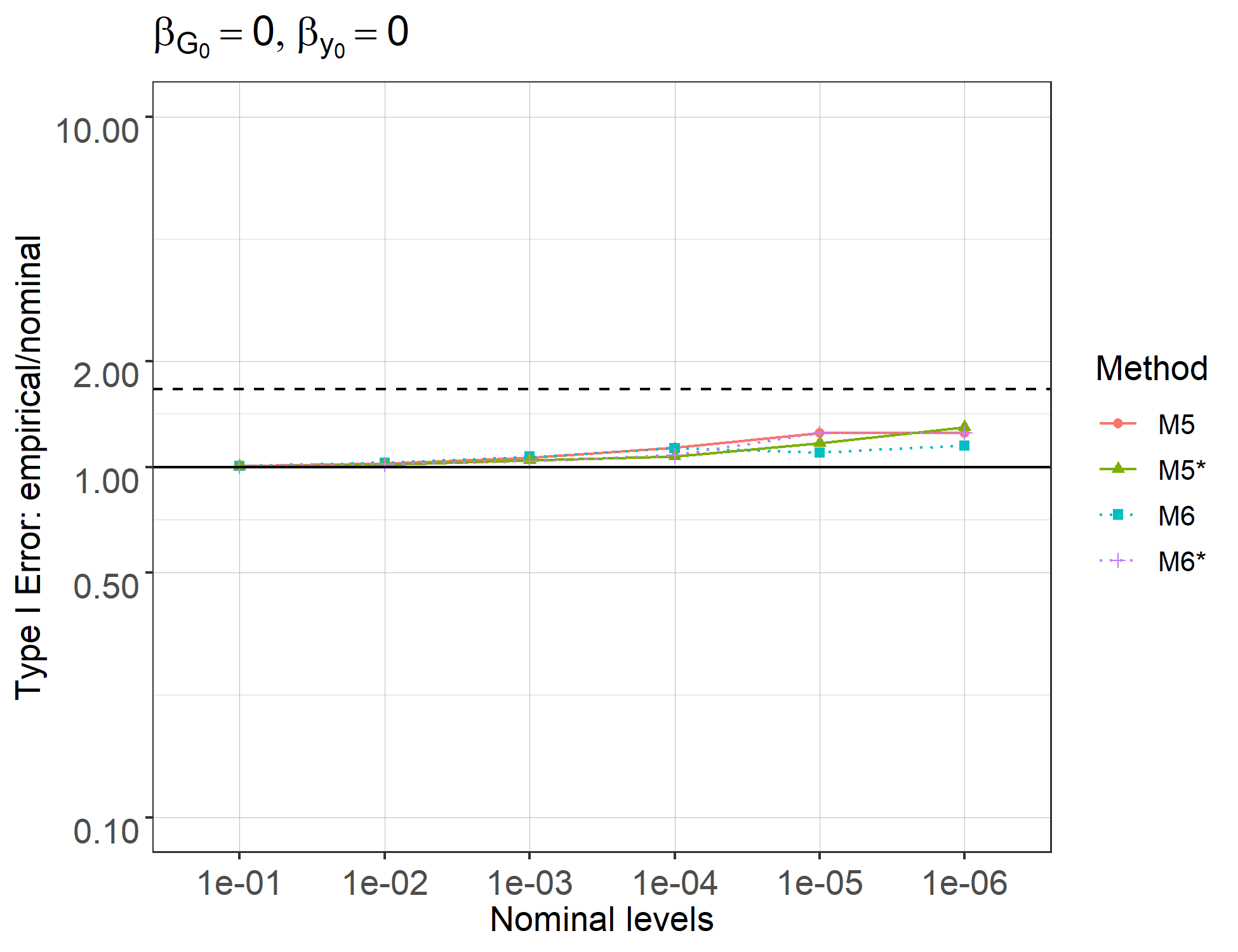


**Supplemental Figure 5**. Ratios between empirical type I error rates and the nominal $\alpha$ levels for M5* and M6* when measurement errors are present. Relative error rates $r_{i} \sim N\left( \mu_{i},\sigma=\frac{1}{4} \right), i=0, 1$. Configuration i: $\mu_{i}\equiv0$. Configuration ii: $\mu_{i}\equiv0.25$. Configuration iii: $\mu_{0}=-0.25, \mu_{1}=0.25$. Horizontal dash line: the ratio ($\alpha$ + 3*SE)/$\alpha$ = 1 + 3*SE/$\alpha\approx1.67$, where SE is the standard error calculated as $\sqrt{\frac{\alpha*(1- \alpha)}{n}}$, $\alpha={10}^{-6}$ is the nominal level and $n=2\times{10}^{7}$ is the number of simulations. M5*and M6* are defined in the Methods section.


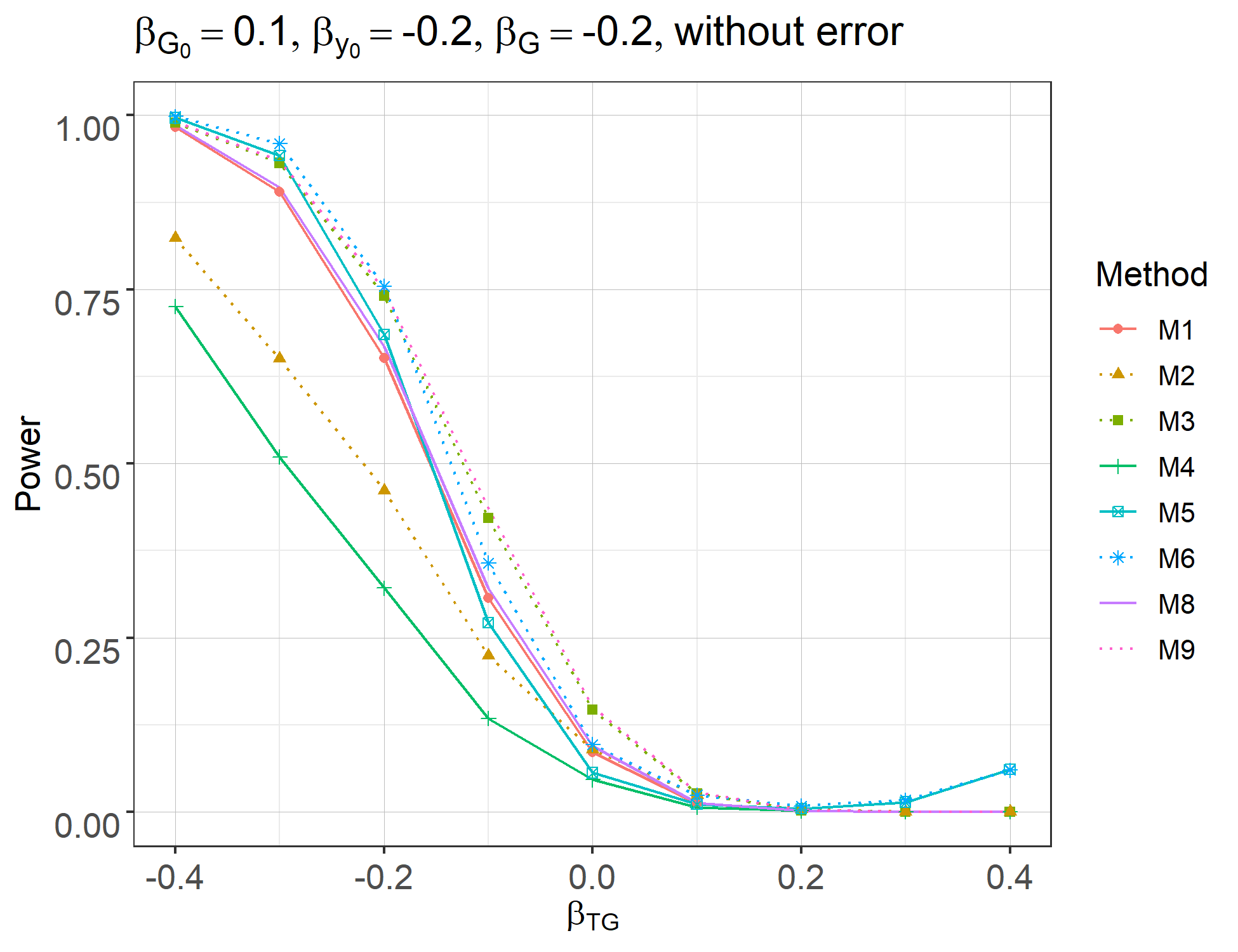

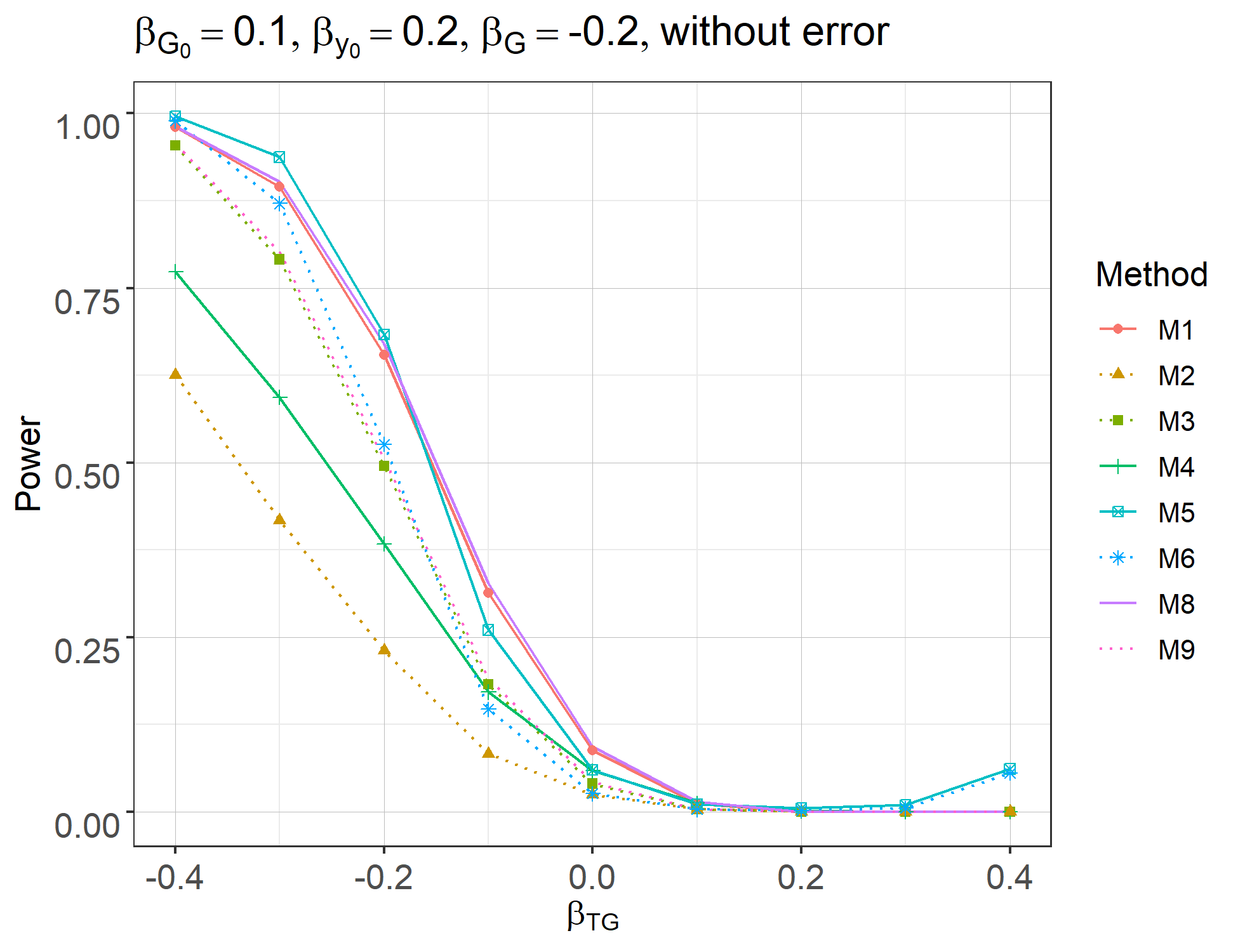

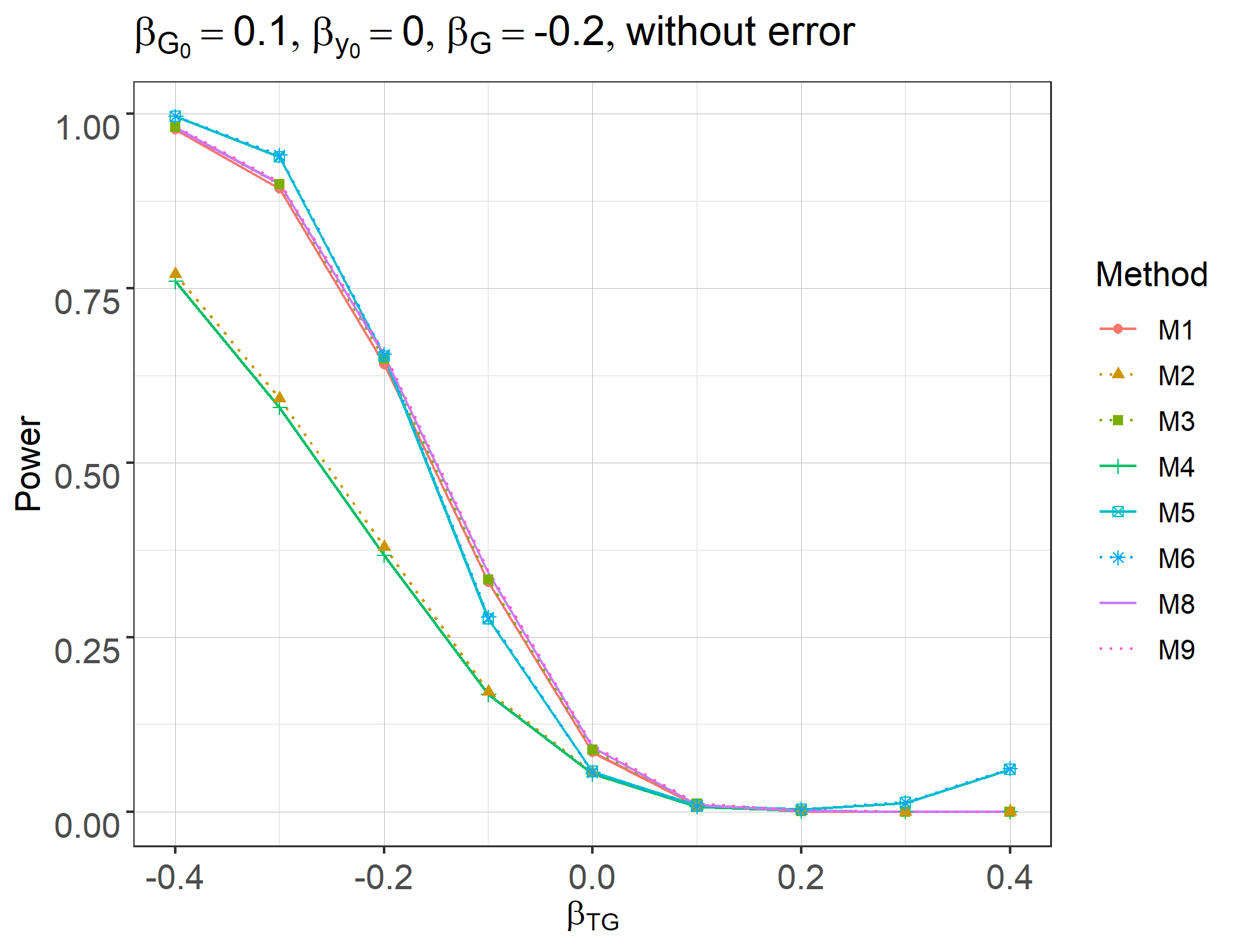

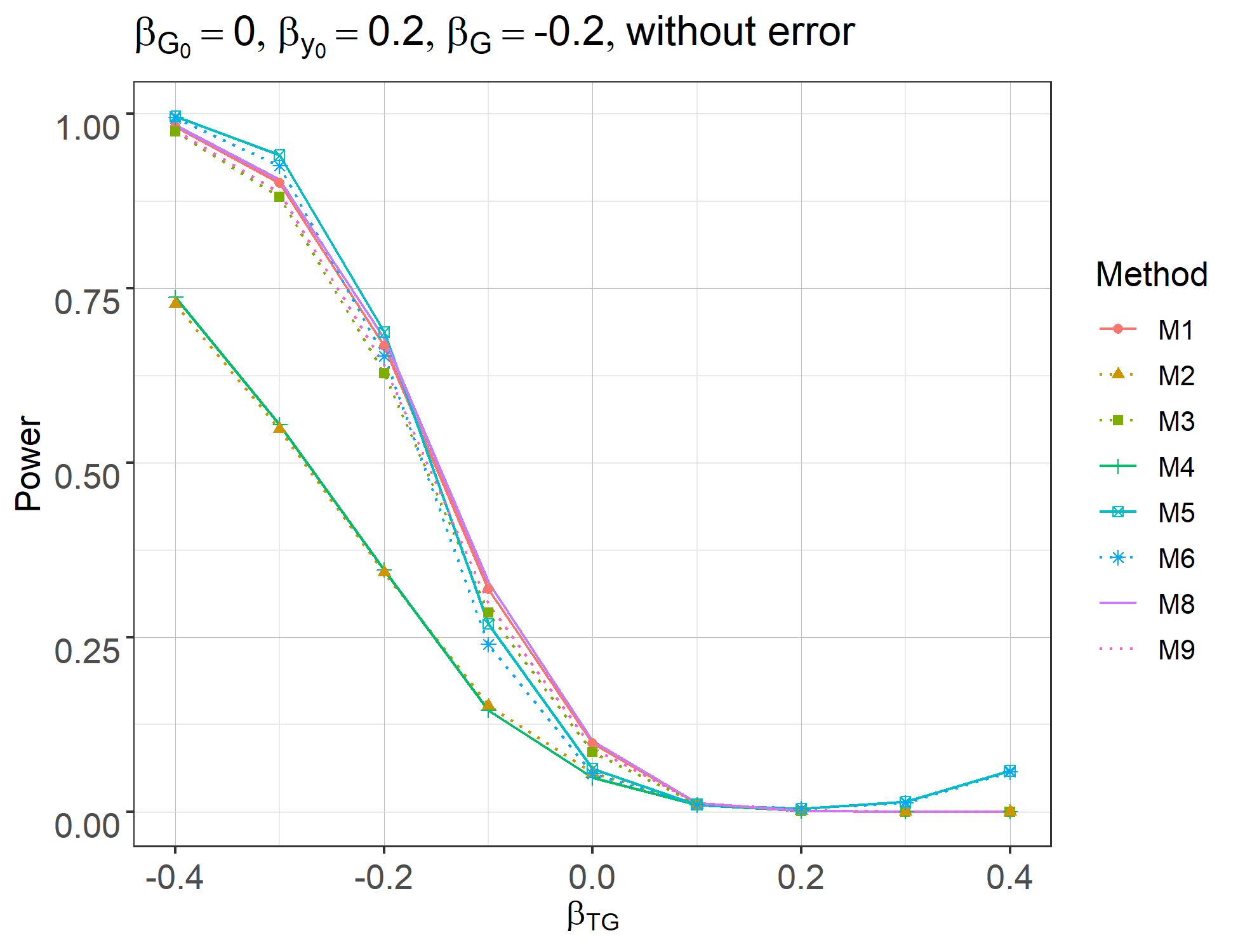

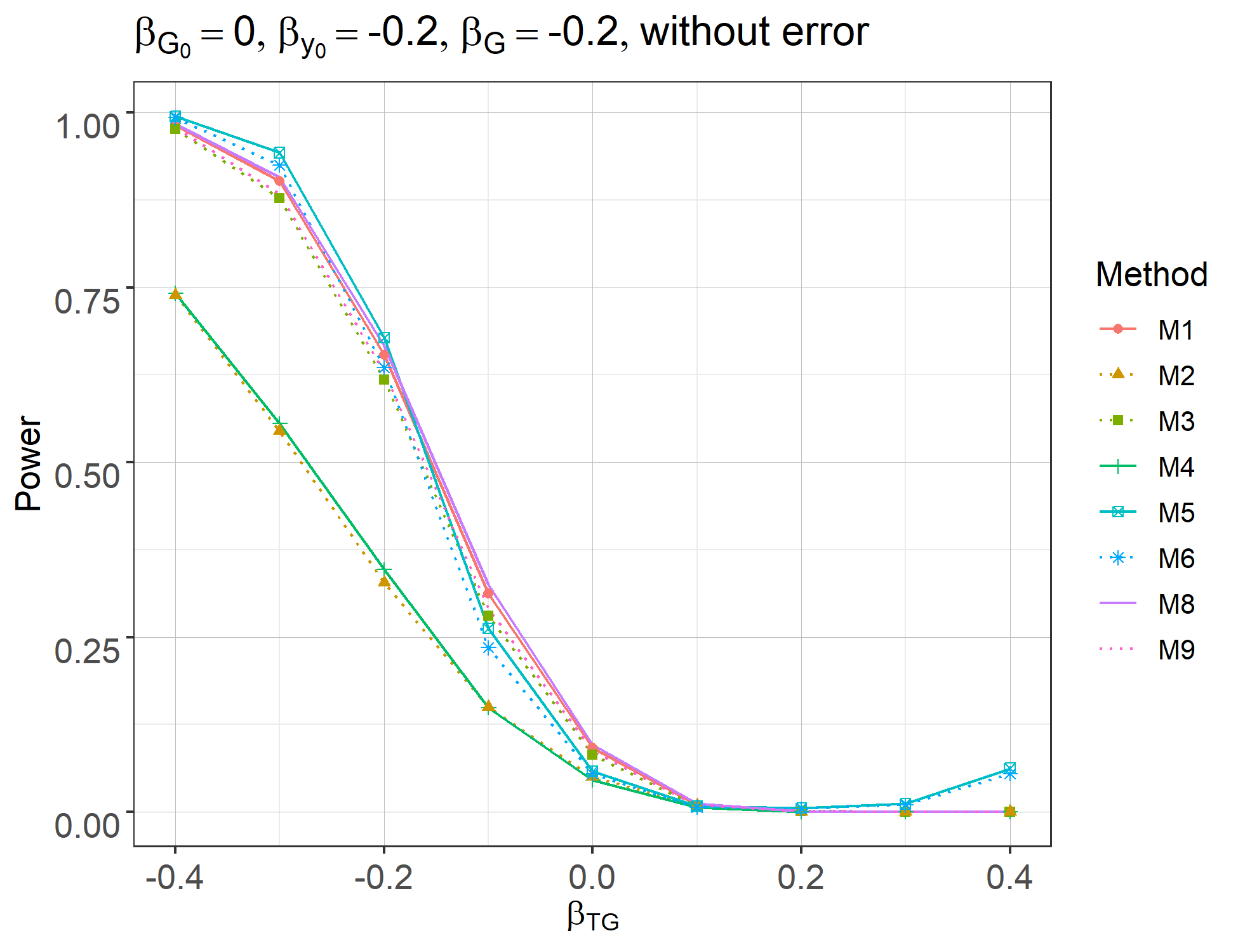

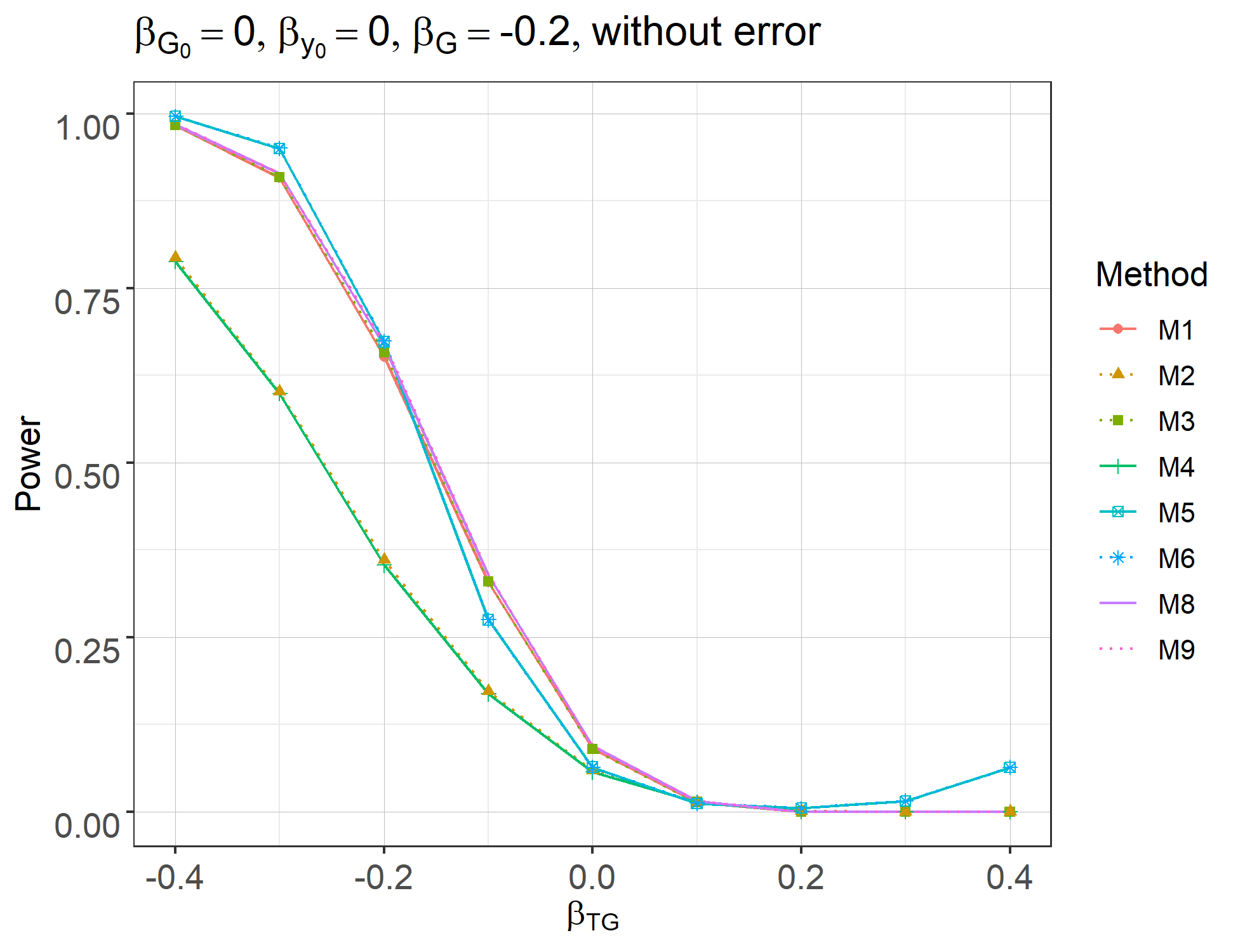

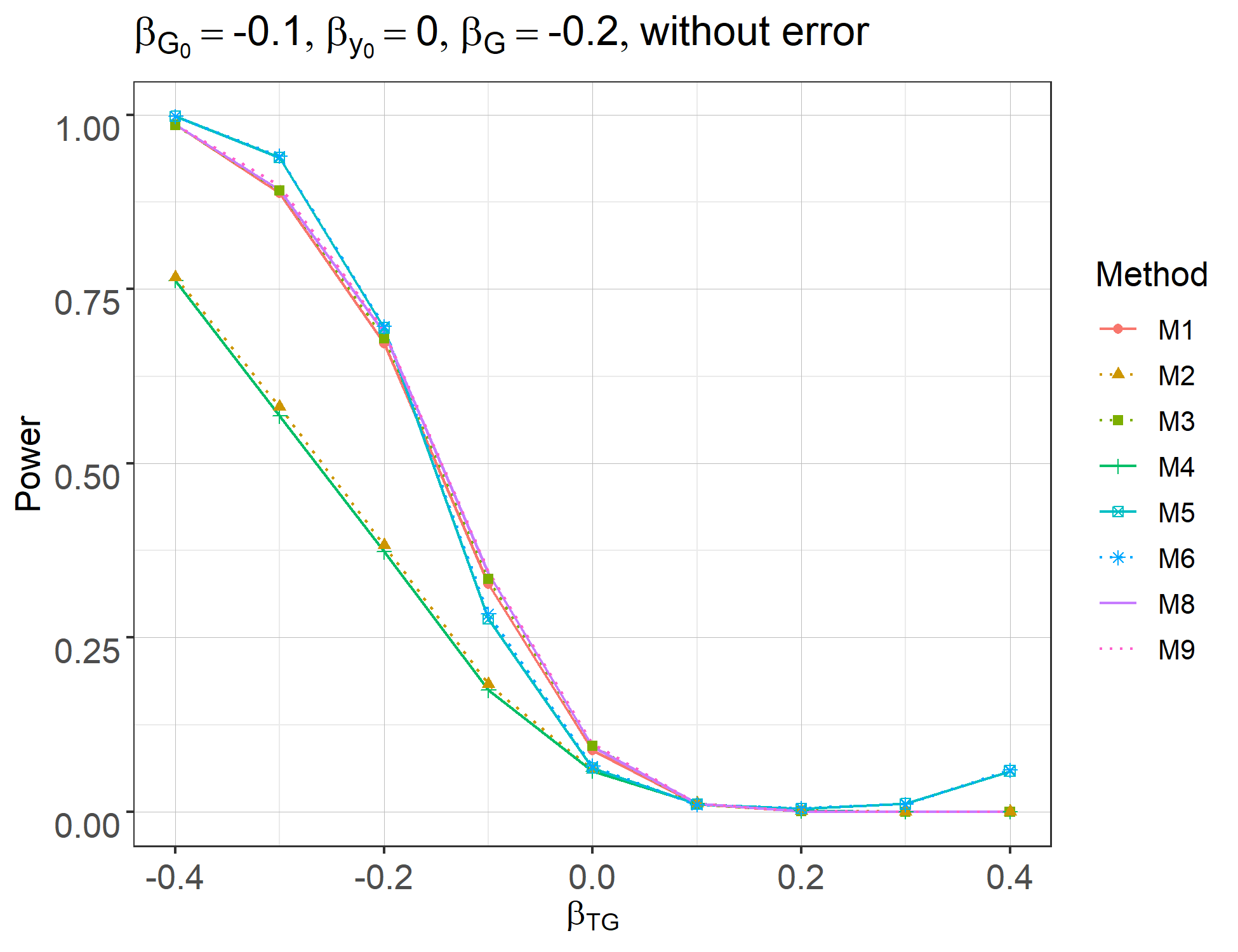

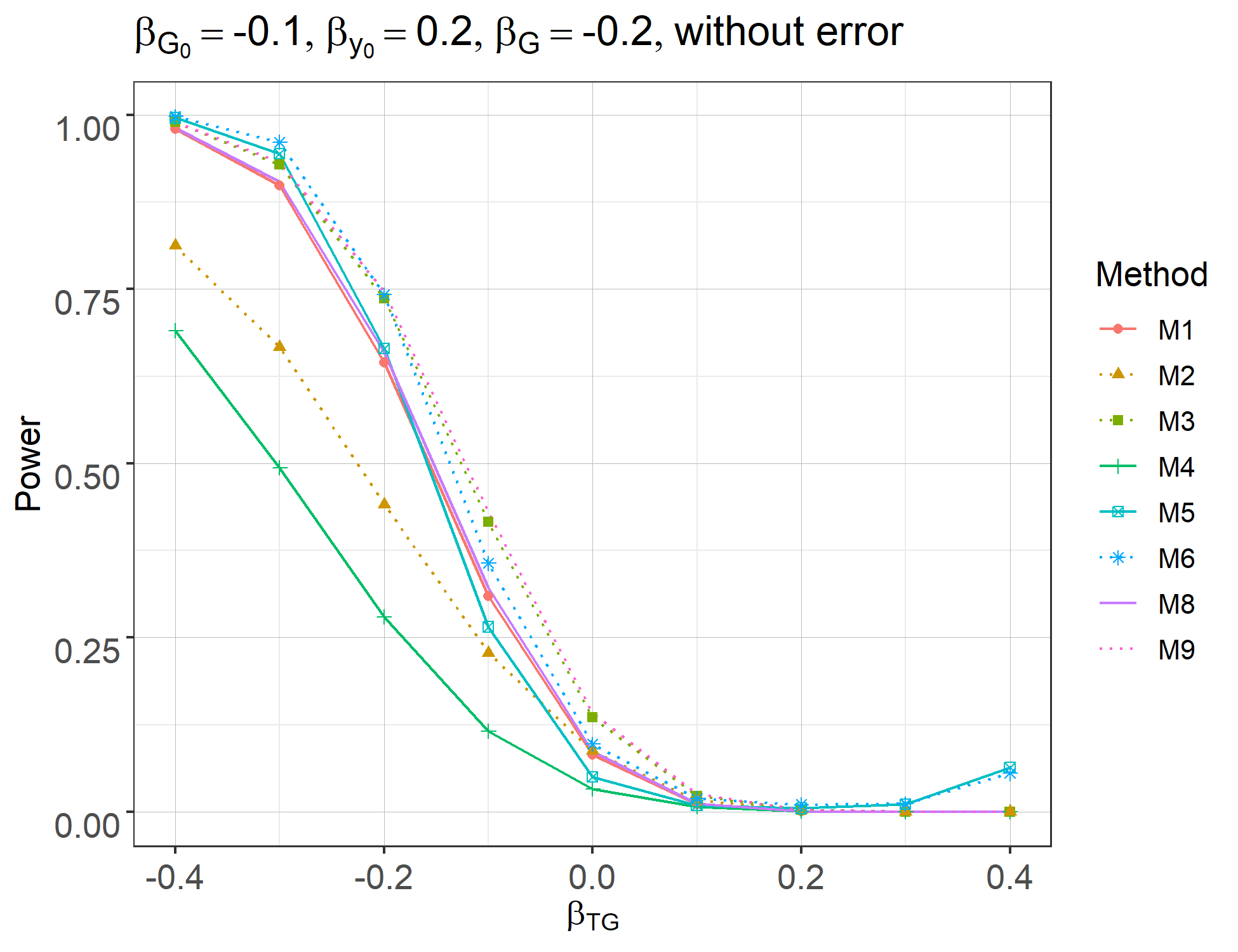

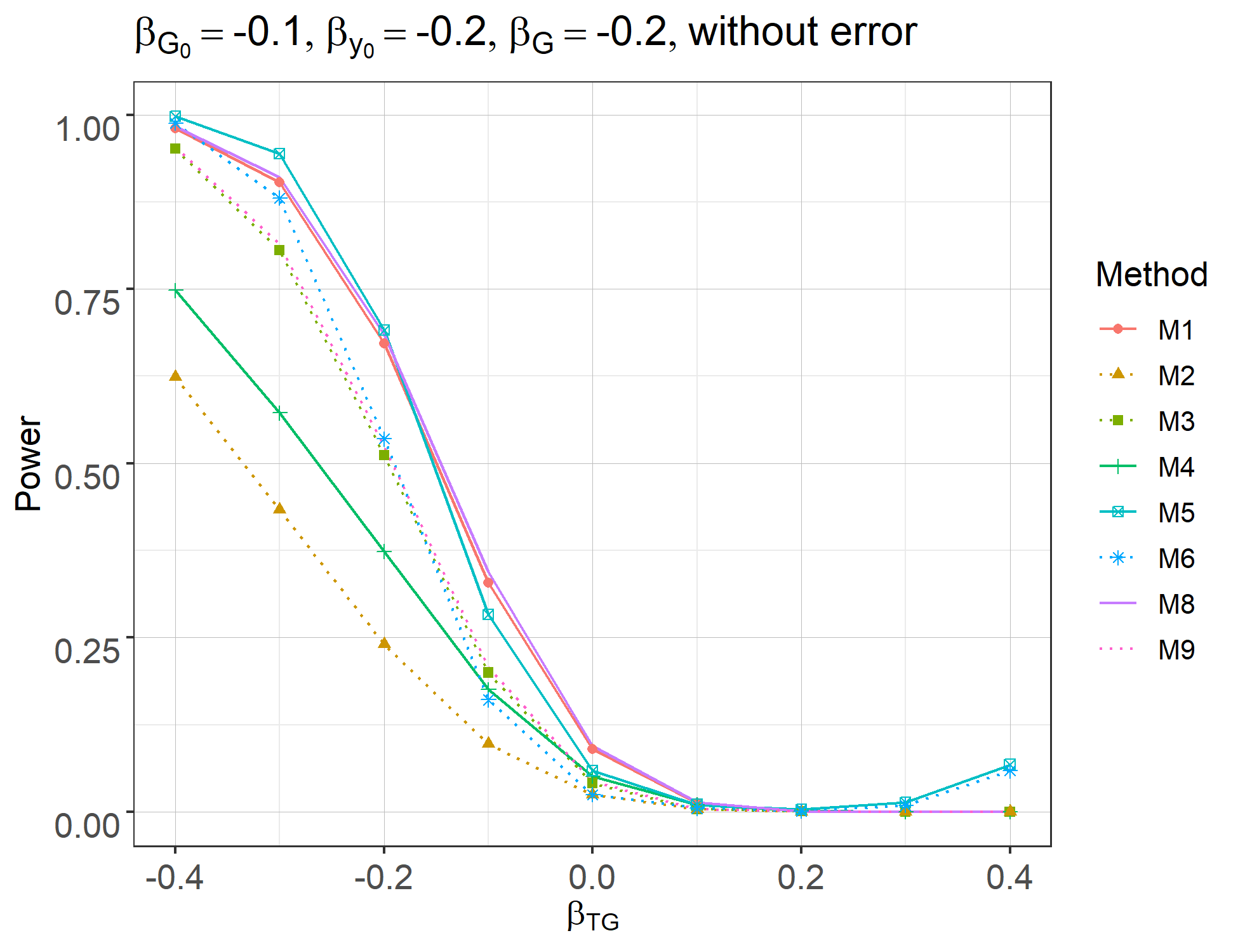


**Supplemental Figure 6**. Power comparison between baseline-adjusted and unadjusted models with genotype effect $\beta_{G}=-0.2$. Left, middle, right column: $\beta_{y_{0}}=0.2, 0, -0.2$, respectively. First, second, third row: $\beta_{G_{0}}=0.1, 0, -0.1$, respectively. $\alpha={10}^{-6}$, which is consistent with the type I error simulation. M1-M9 are defined in the Methods section.


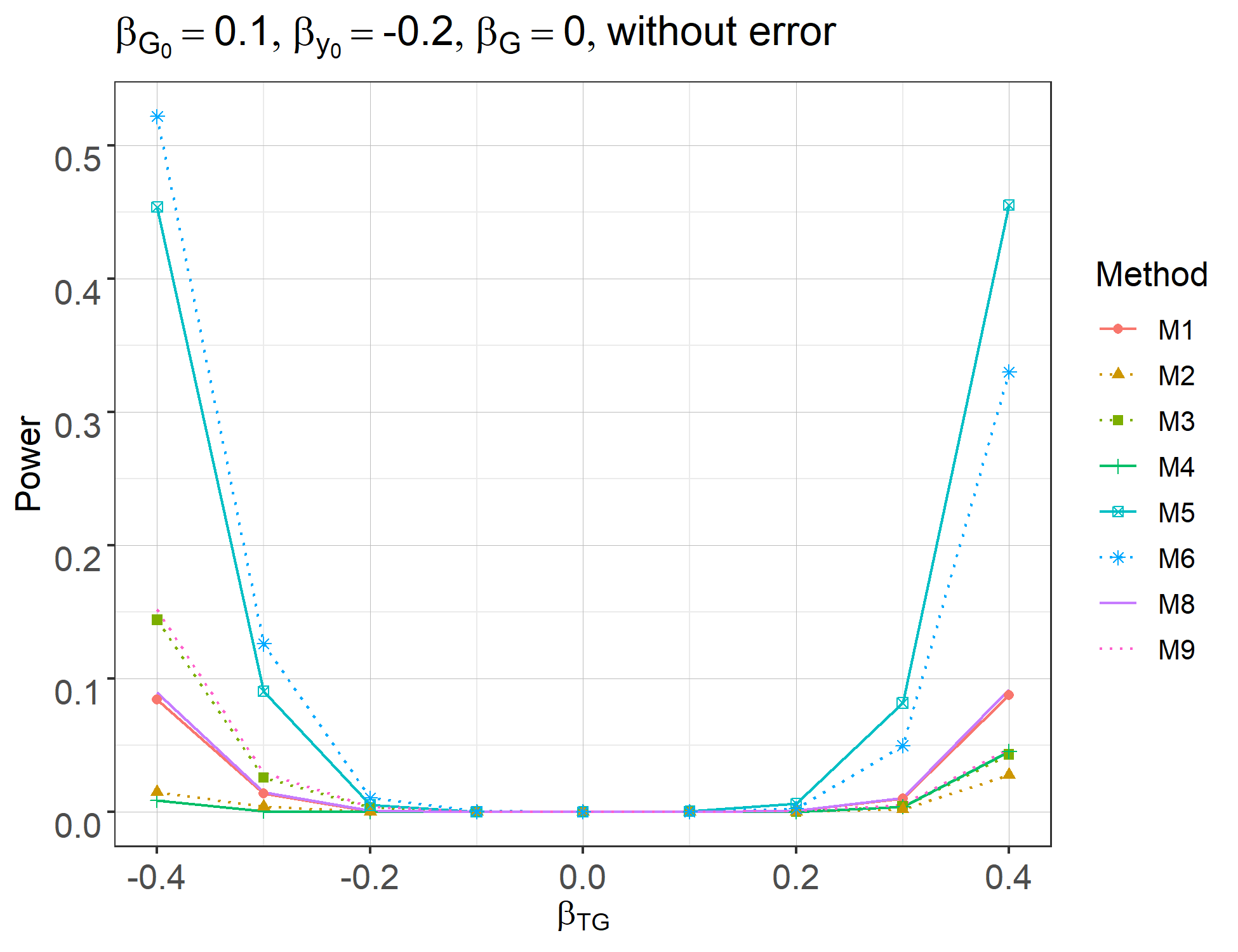

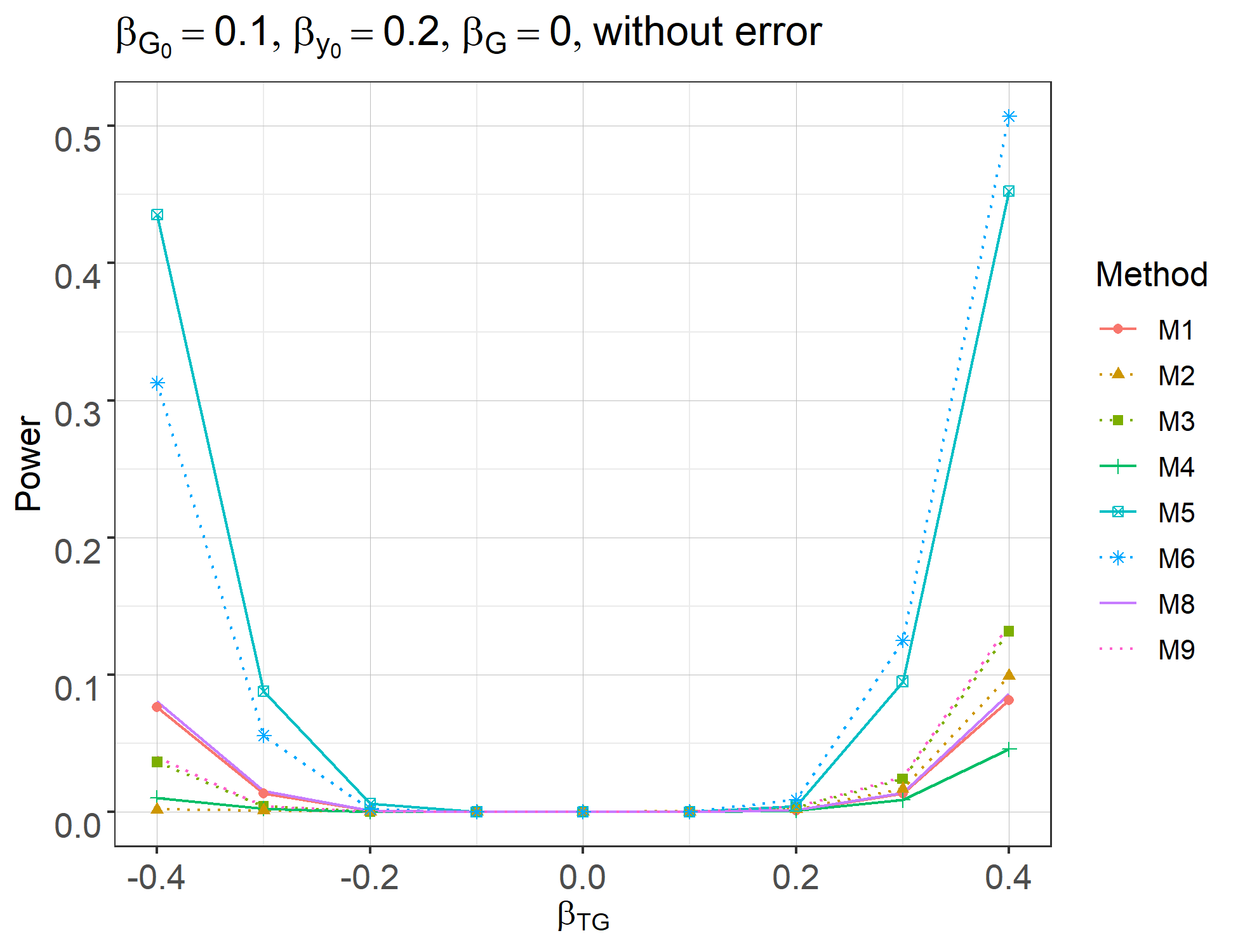

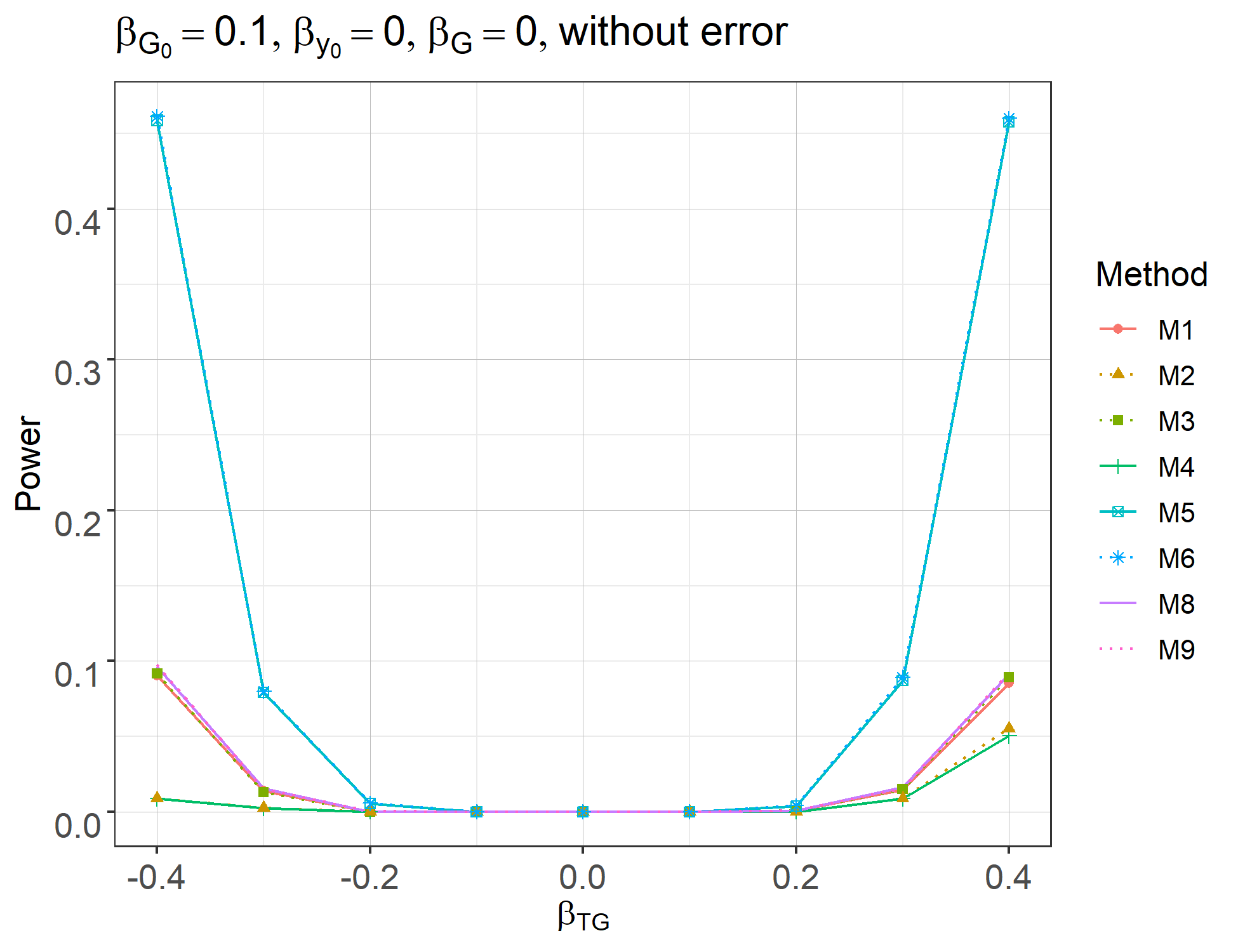

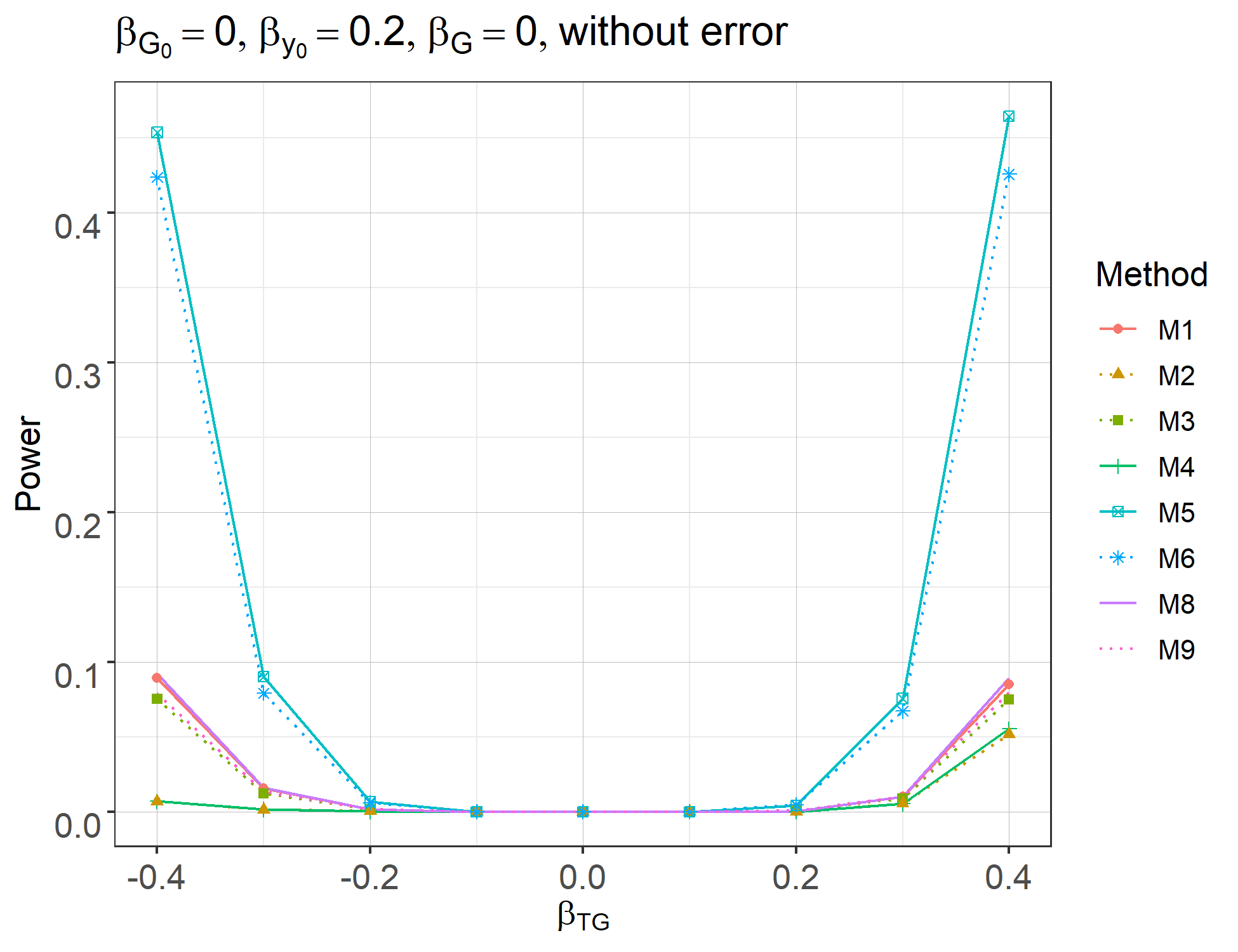

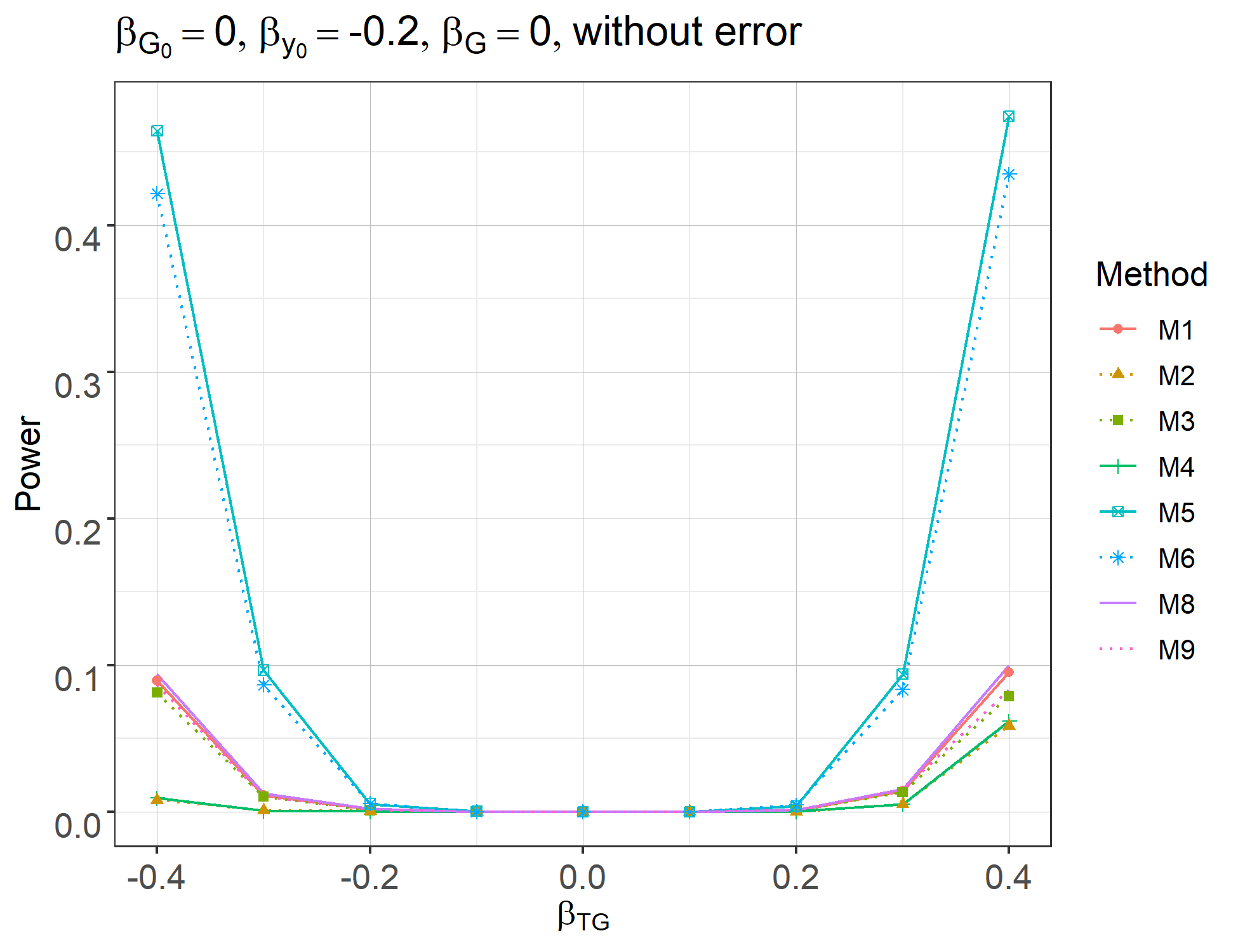

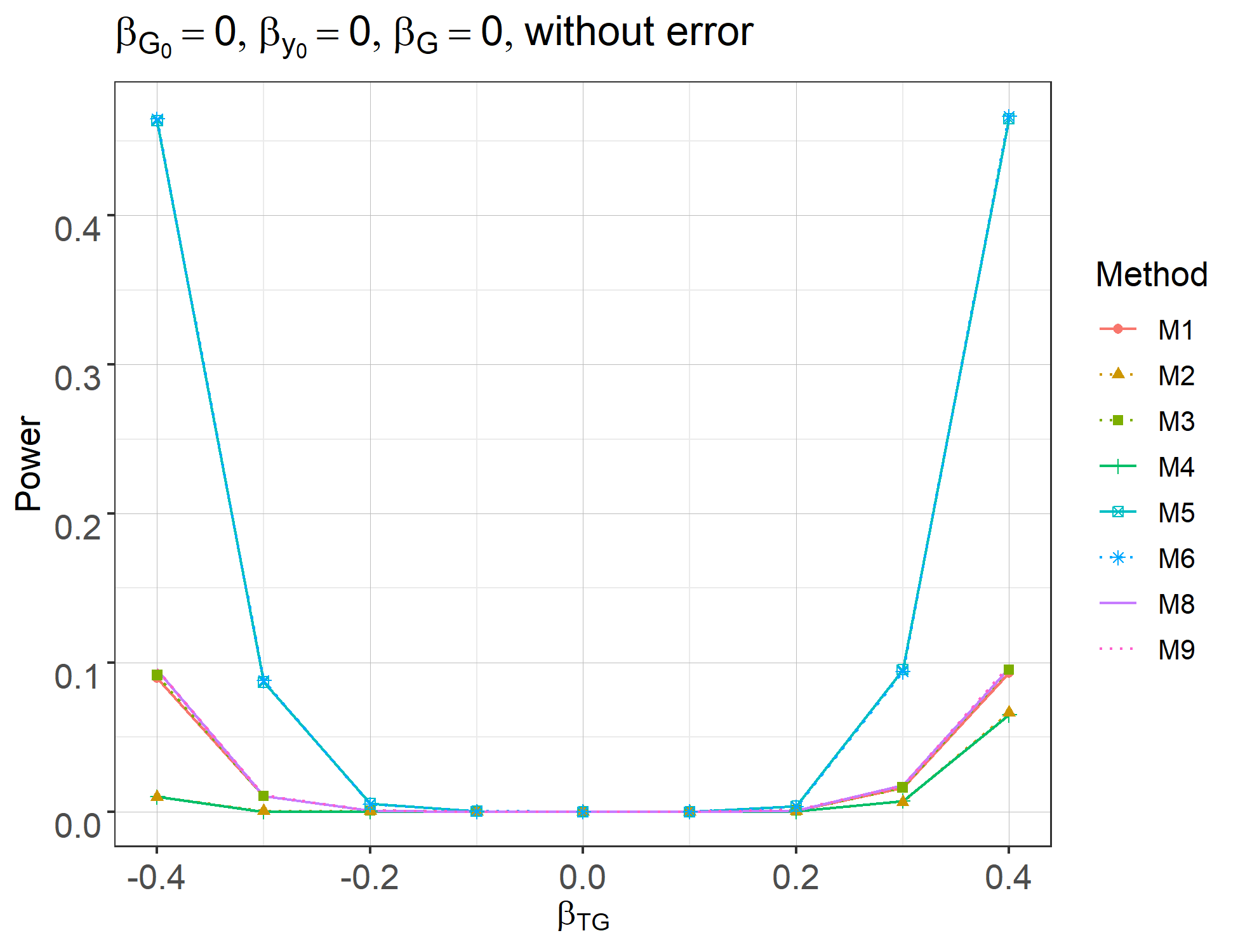

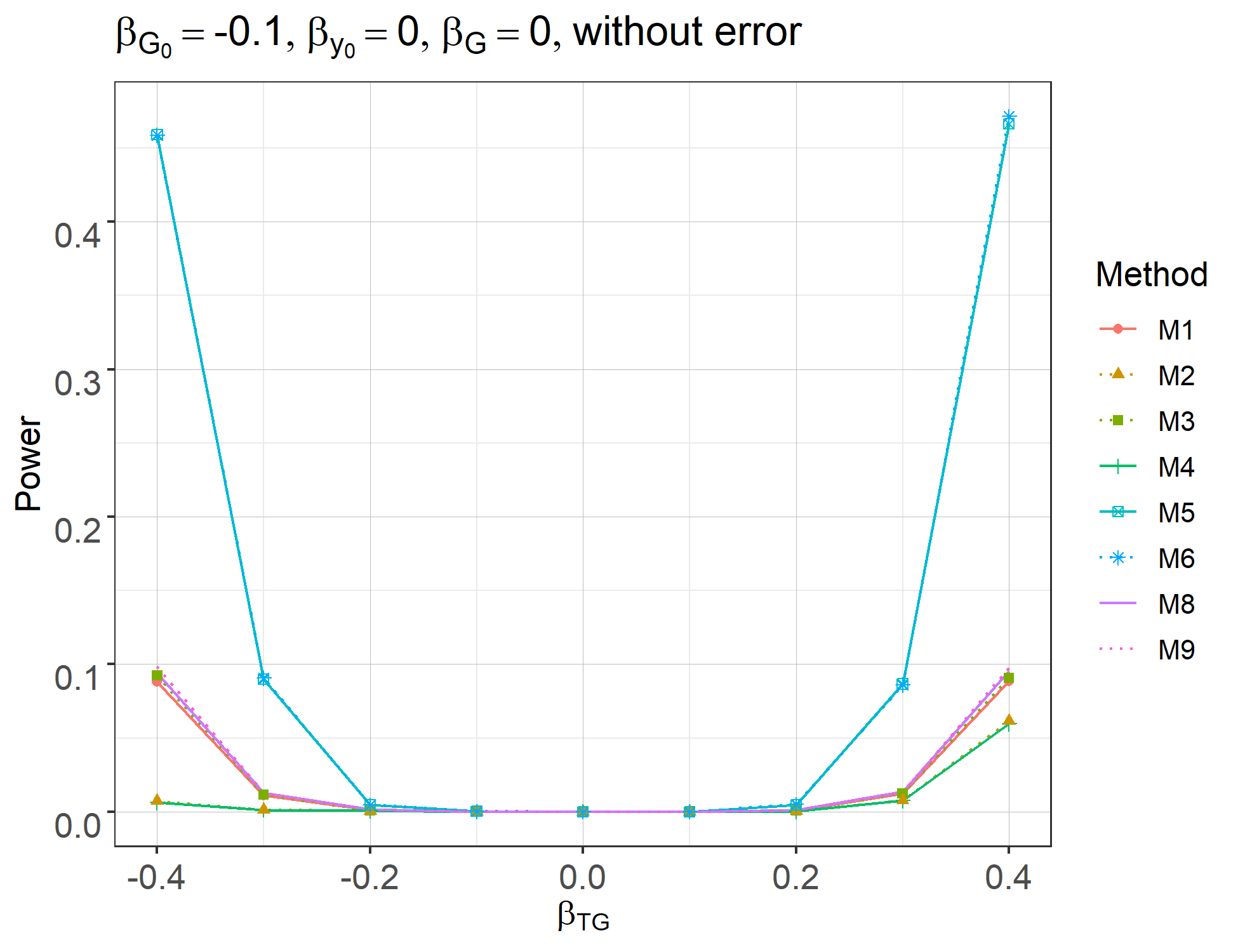

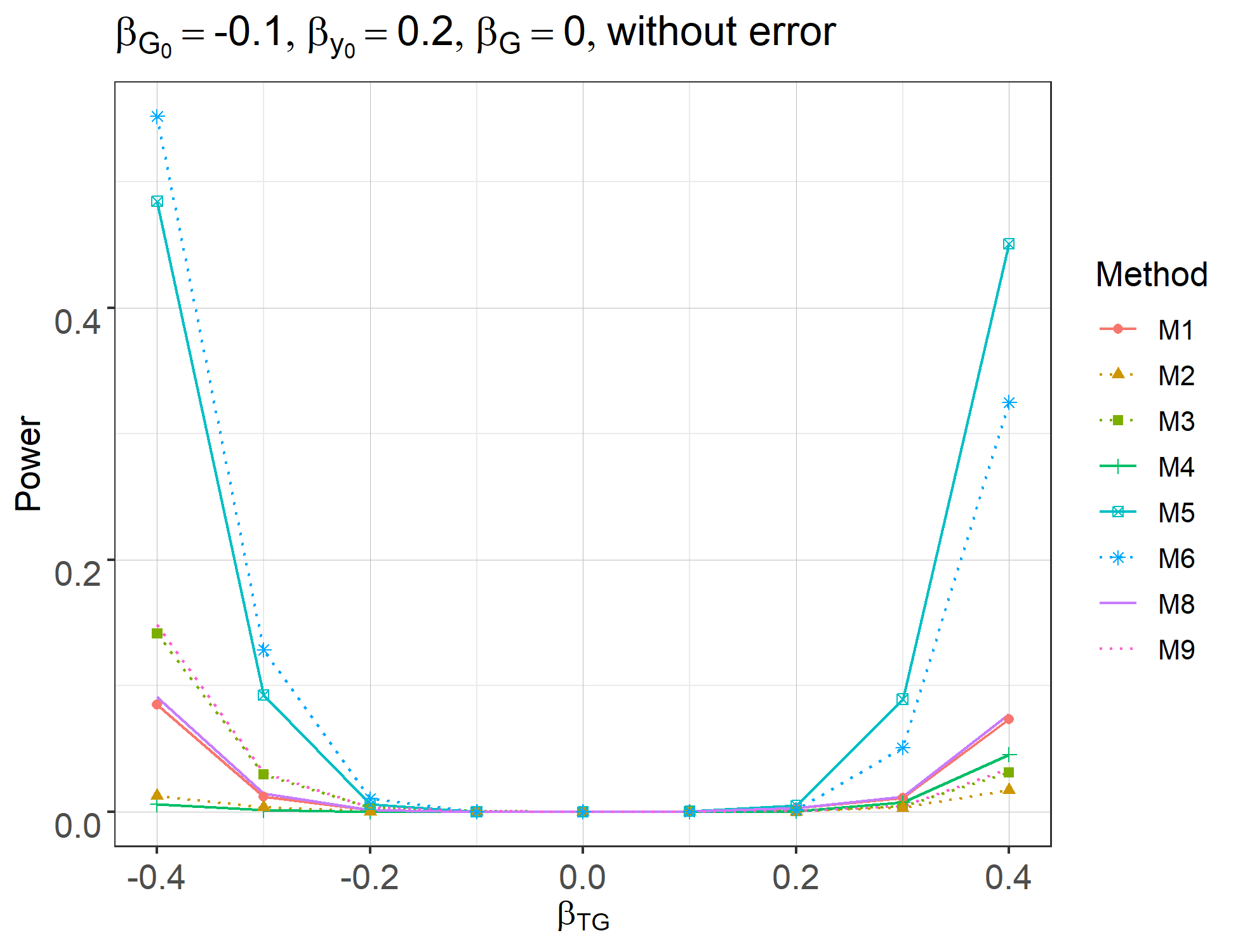

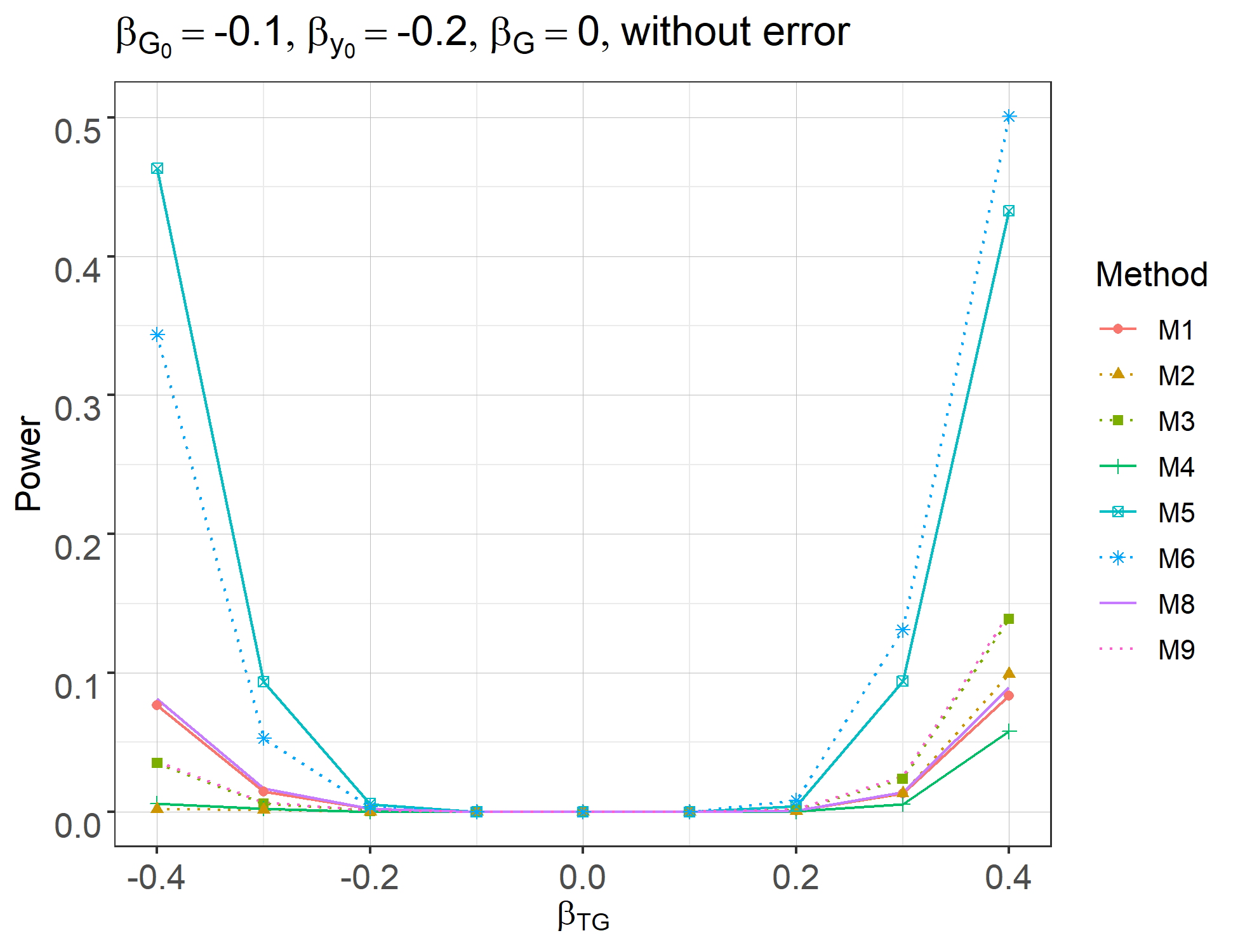


**Supplemental Figure 7**. Power comparison between baseline-adjusted and unadjusted models with genotype effect $\beta_{G}=0$. Left, middle, right column: $\beta_{y_{0}}=0.2, 0, -0.2$, respectively. First, second, third row: $\beta_{G_{0}}=0.1, 0, -0.1$, respectively. $\alpha={10}^{-6}$, which is consistent with the type I error simulation. M1-M9 are defined in the Methods section.


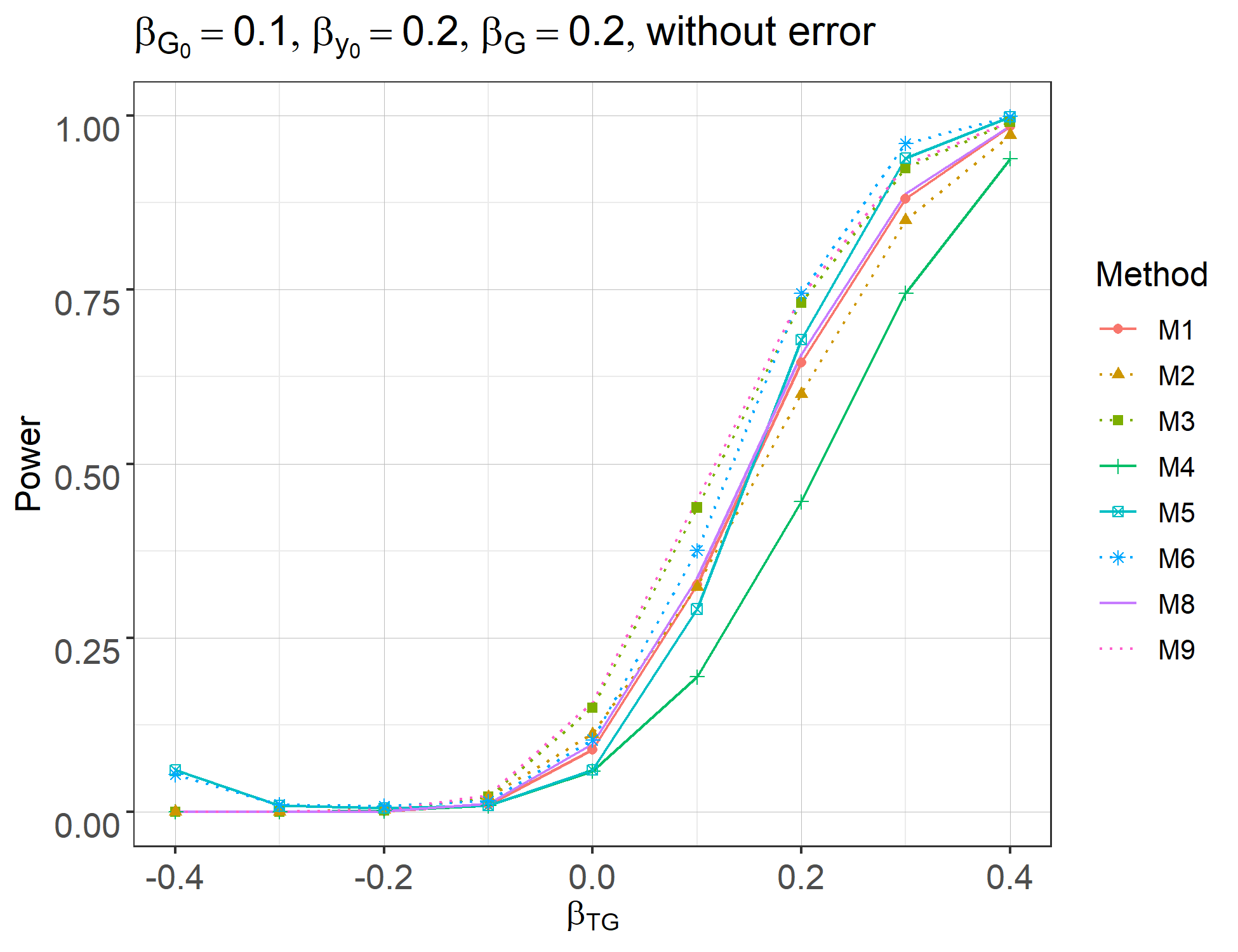

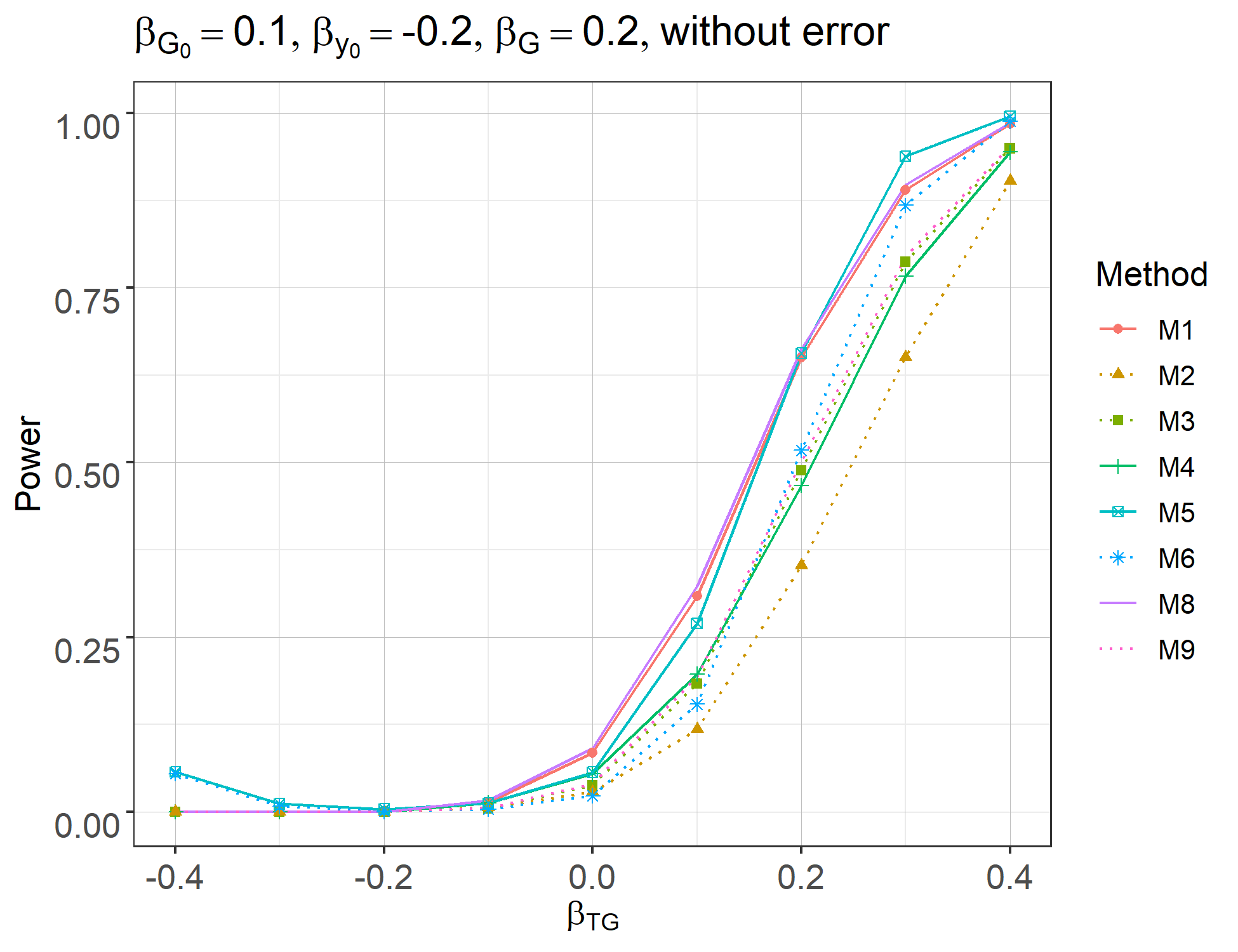

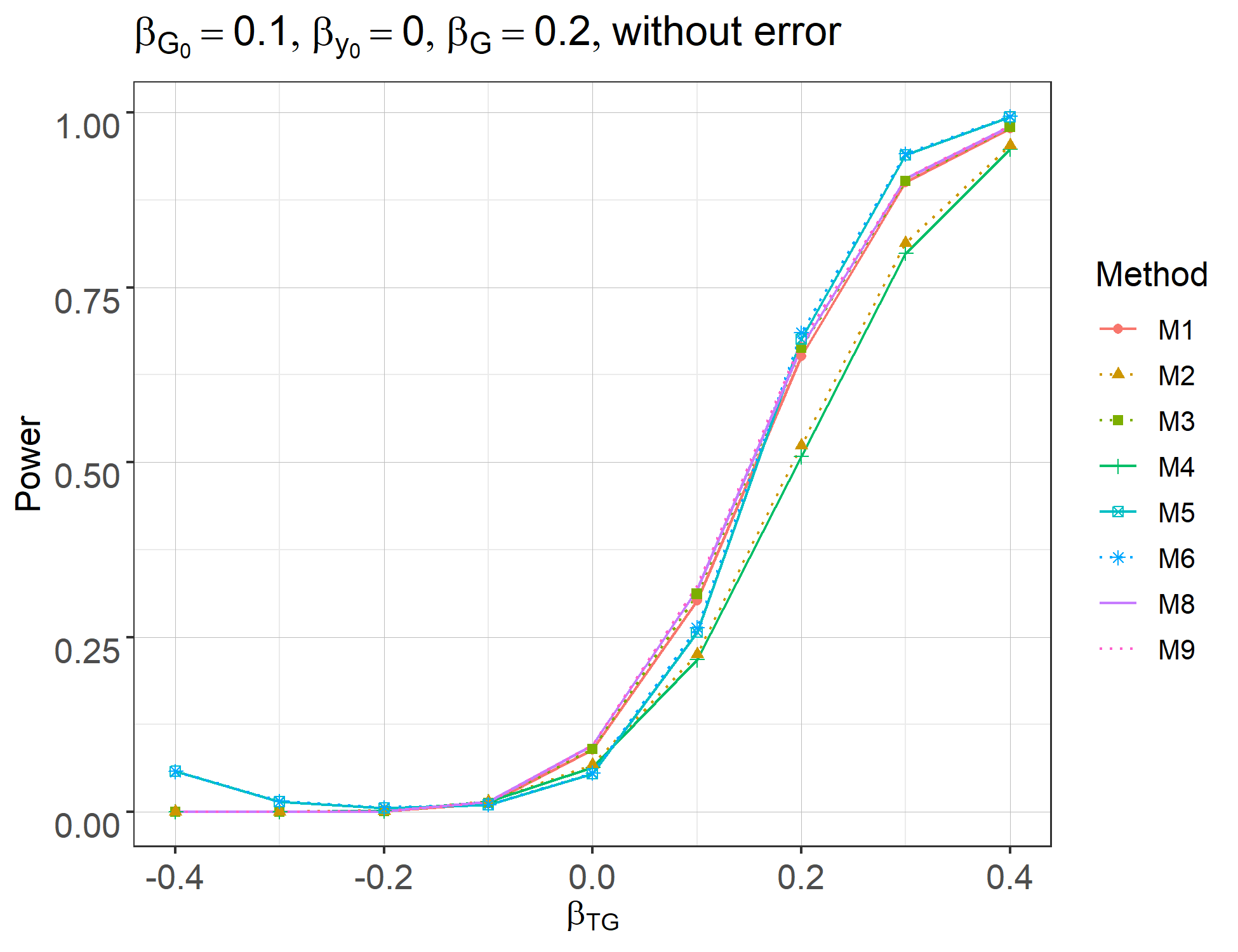

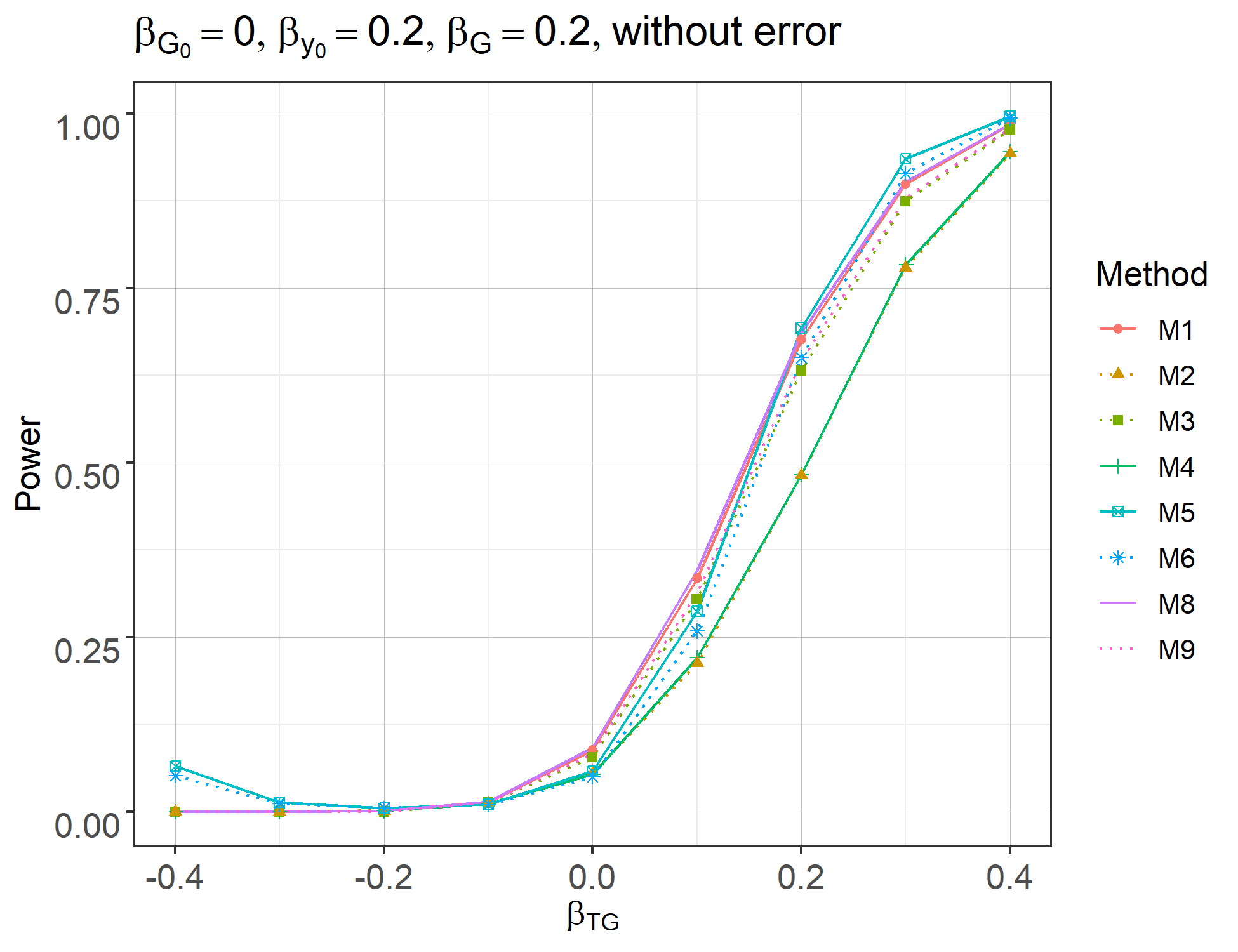

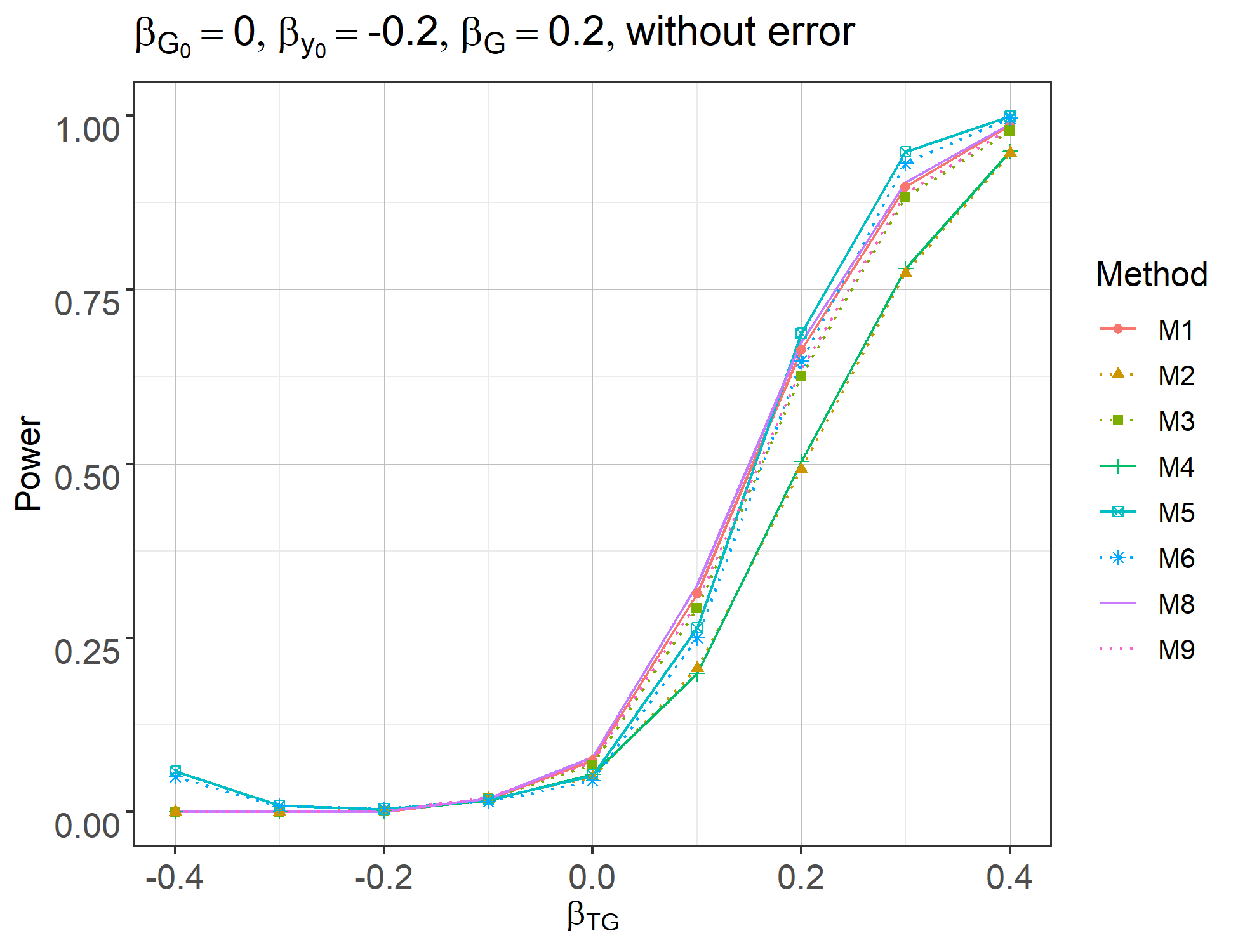

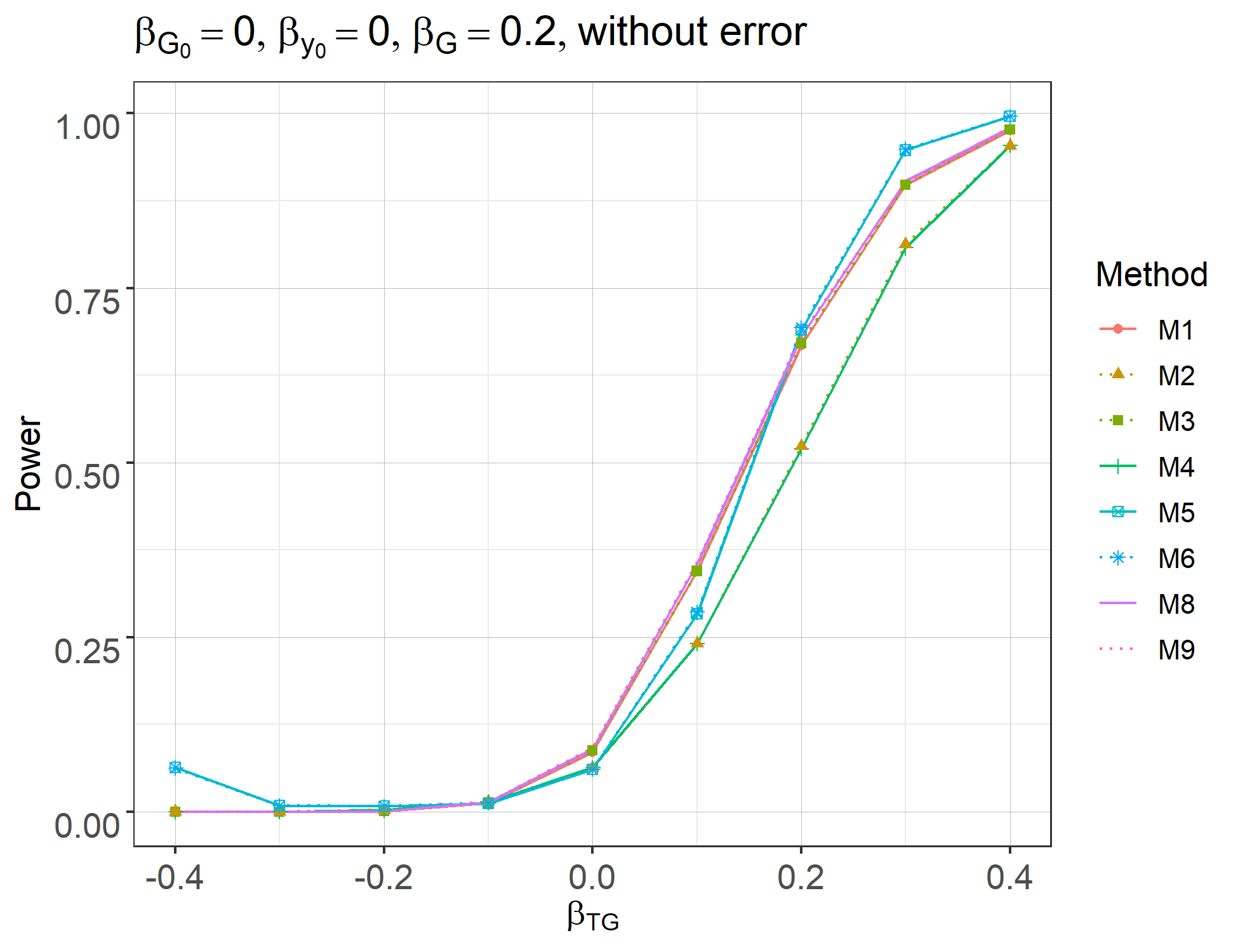

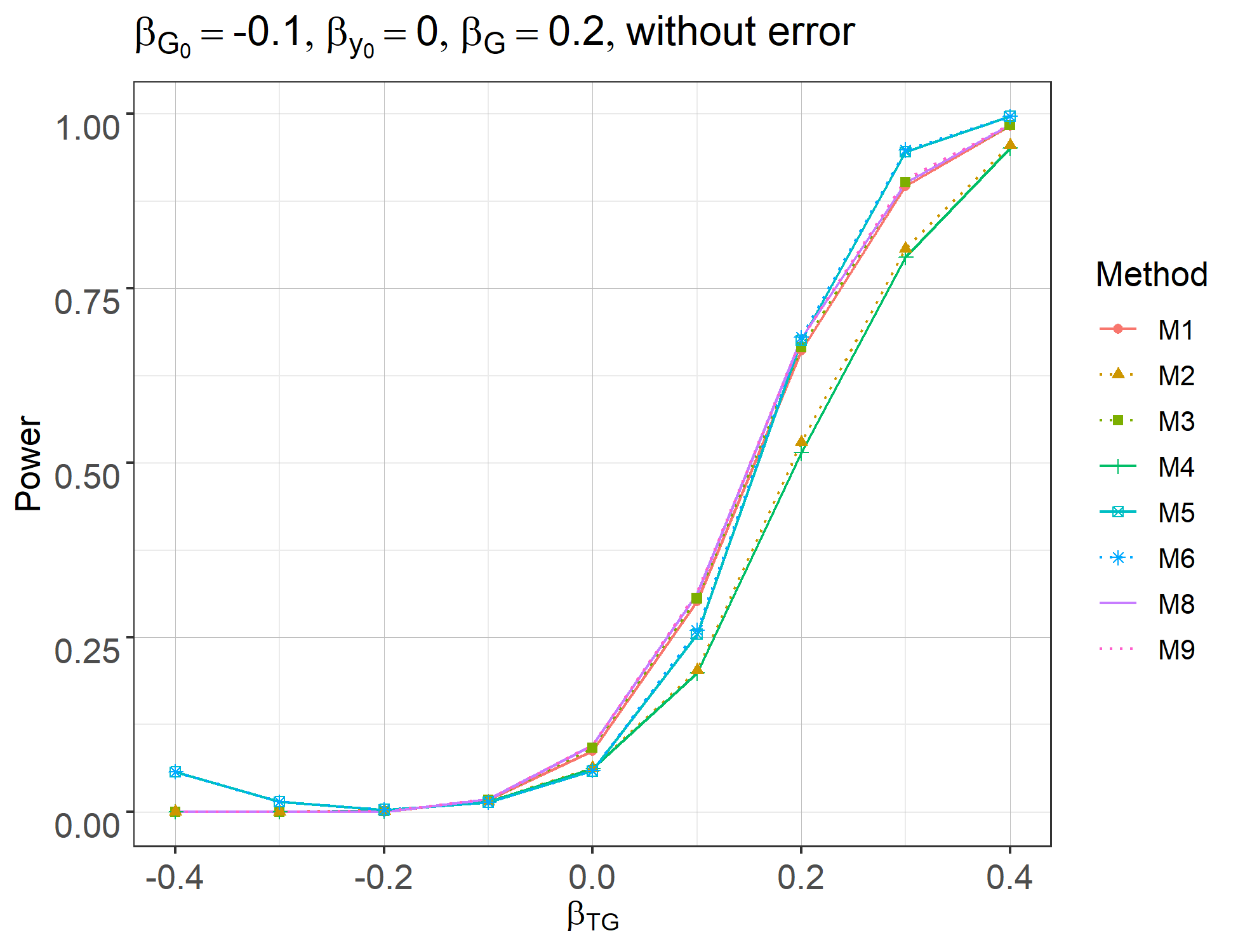

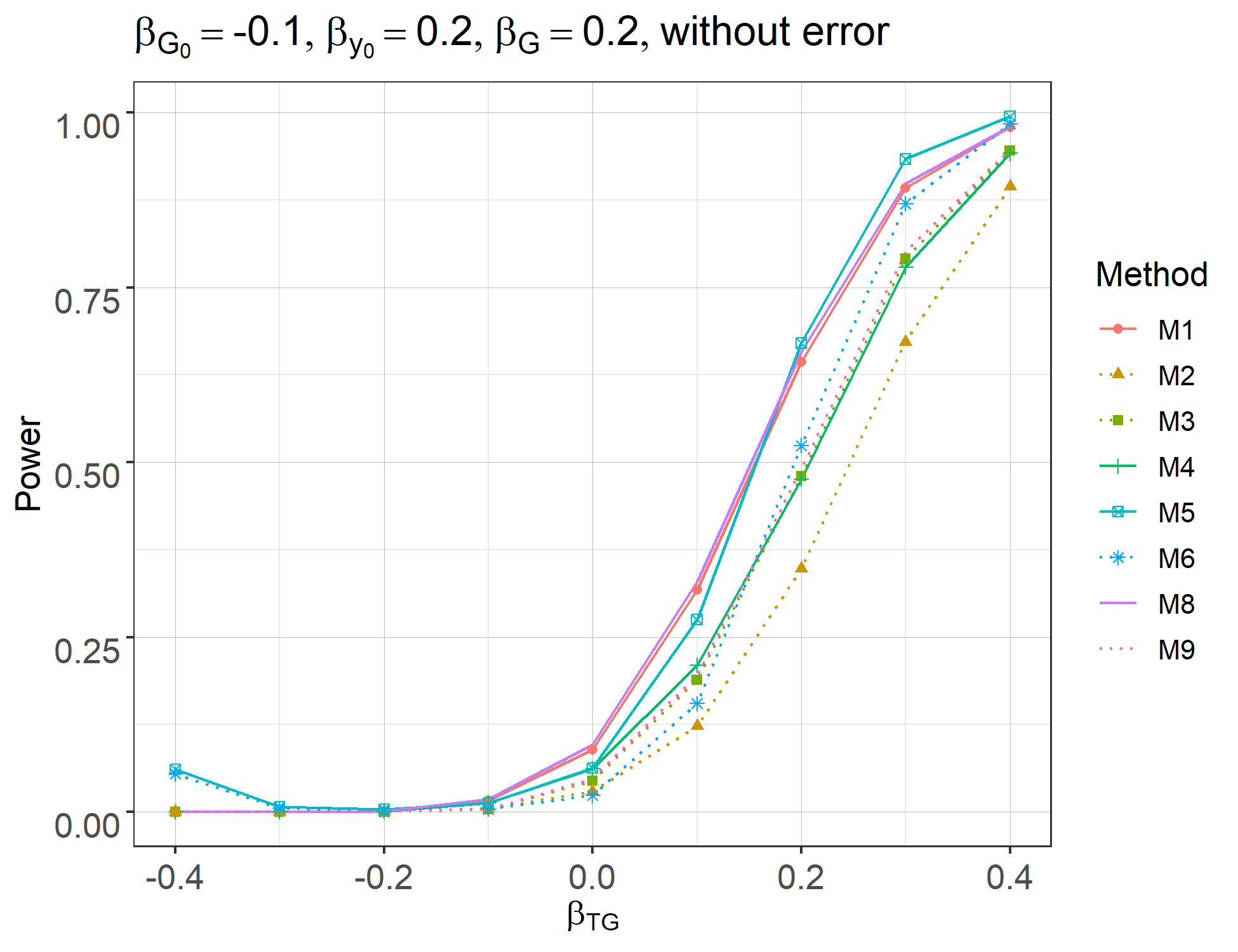

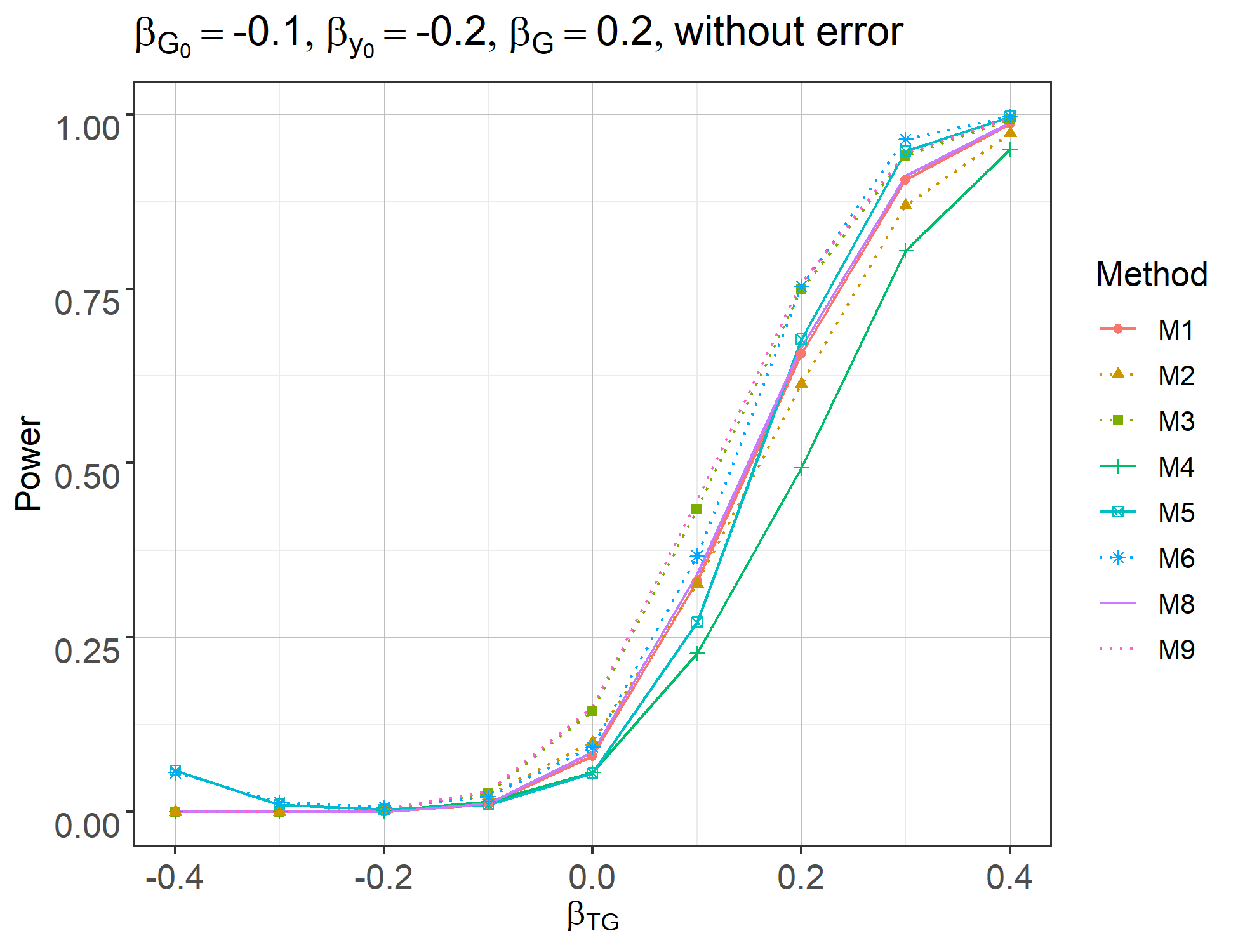


**Supplemental Figure 8**. Power comparison between baseline-adjusted and unadjusted models with genotype effect $\beta_{G}=0.2$. Left, middle, right column: $\beta_{y_{0}}=0.2, 0, -0.2$, respectively. First, second, third row: $\beta_{G_{0}}=0.1, 0, -0.1$, respectively. $\alpha={10}^{-6}$, which is consistent with the type I error simulation. M1-M9 are defined in the Methods section.


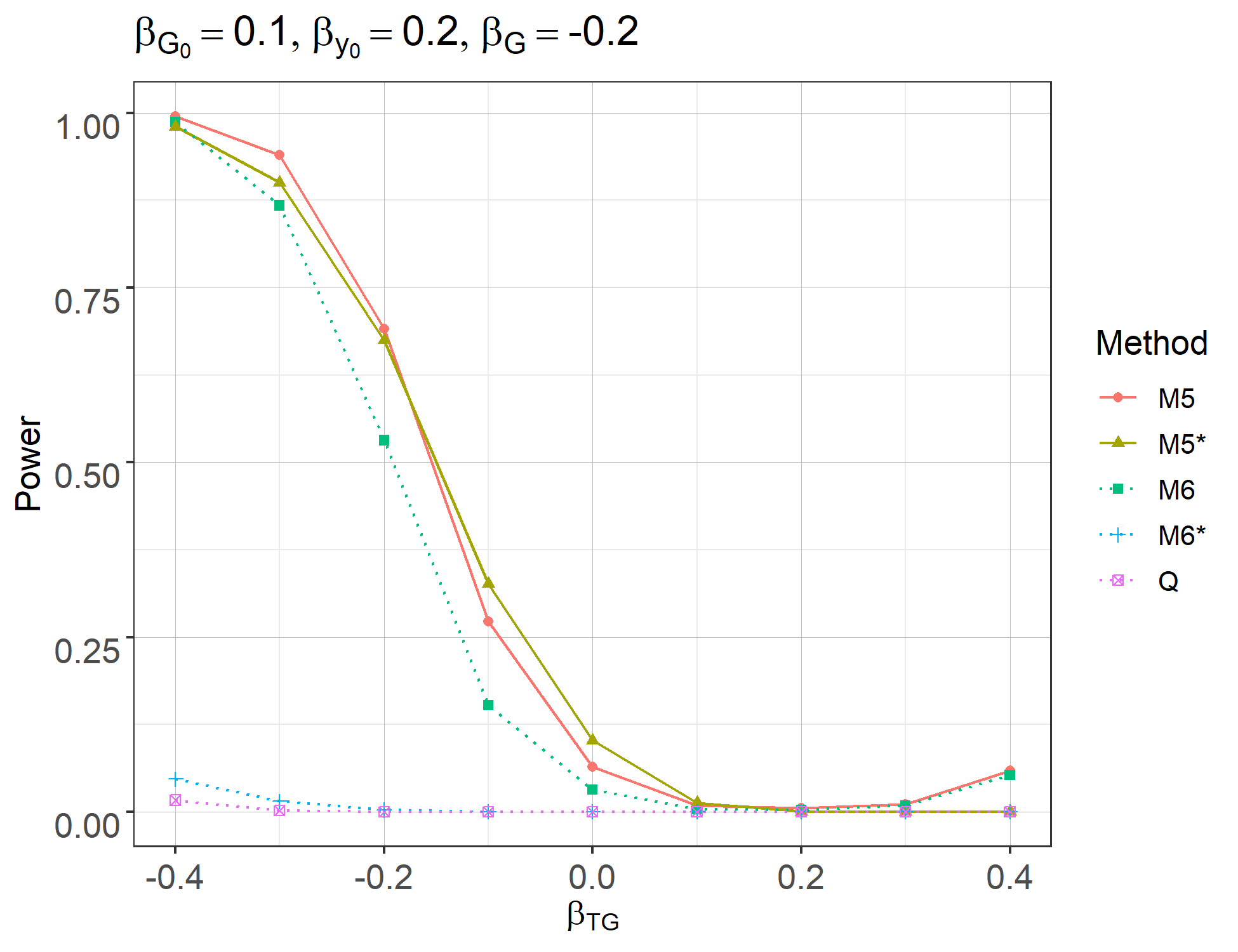

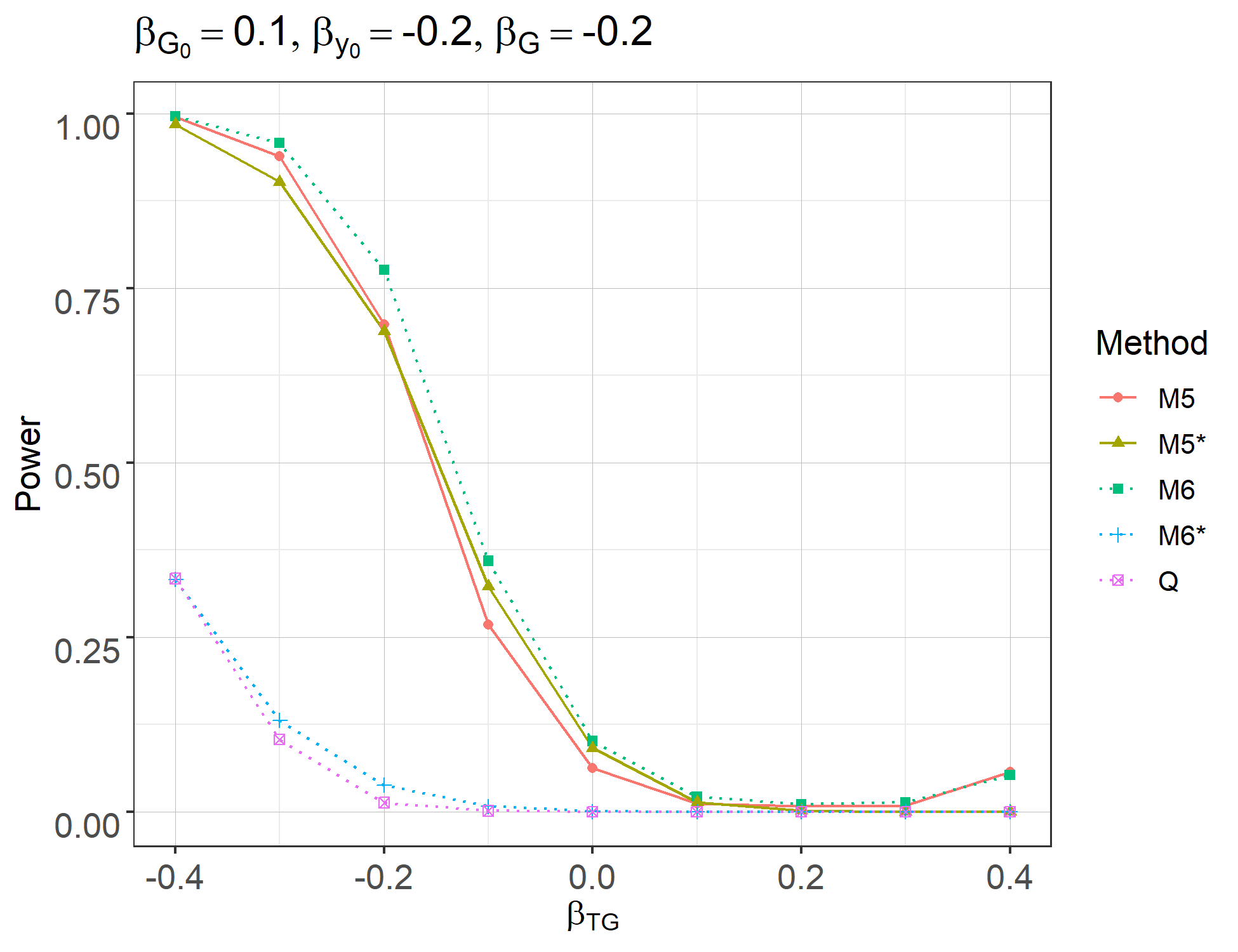

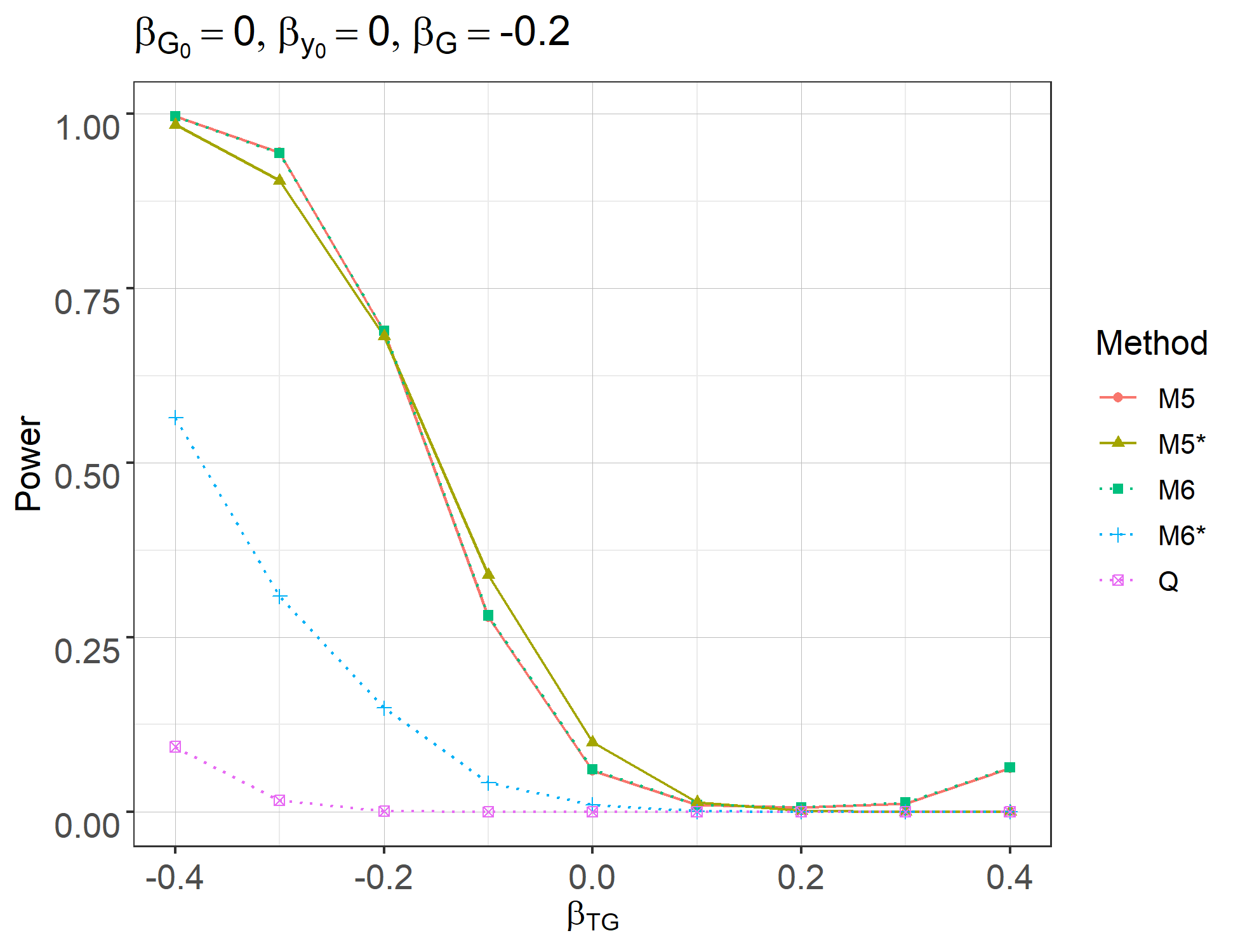

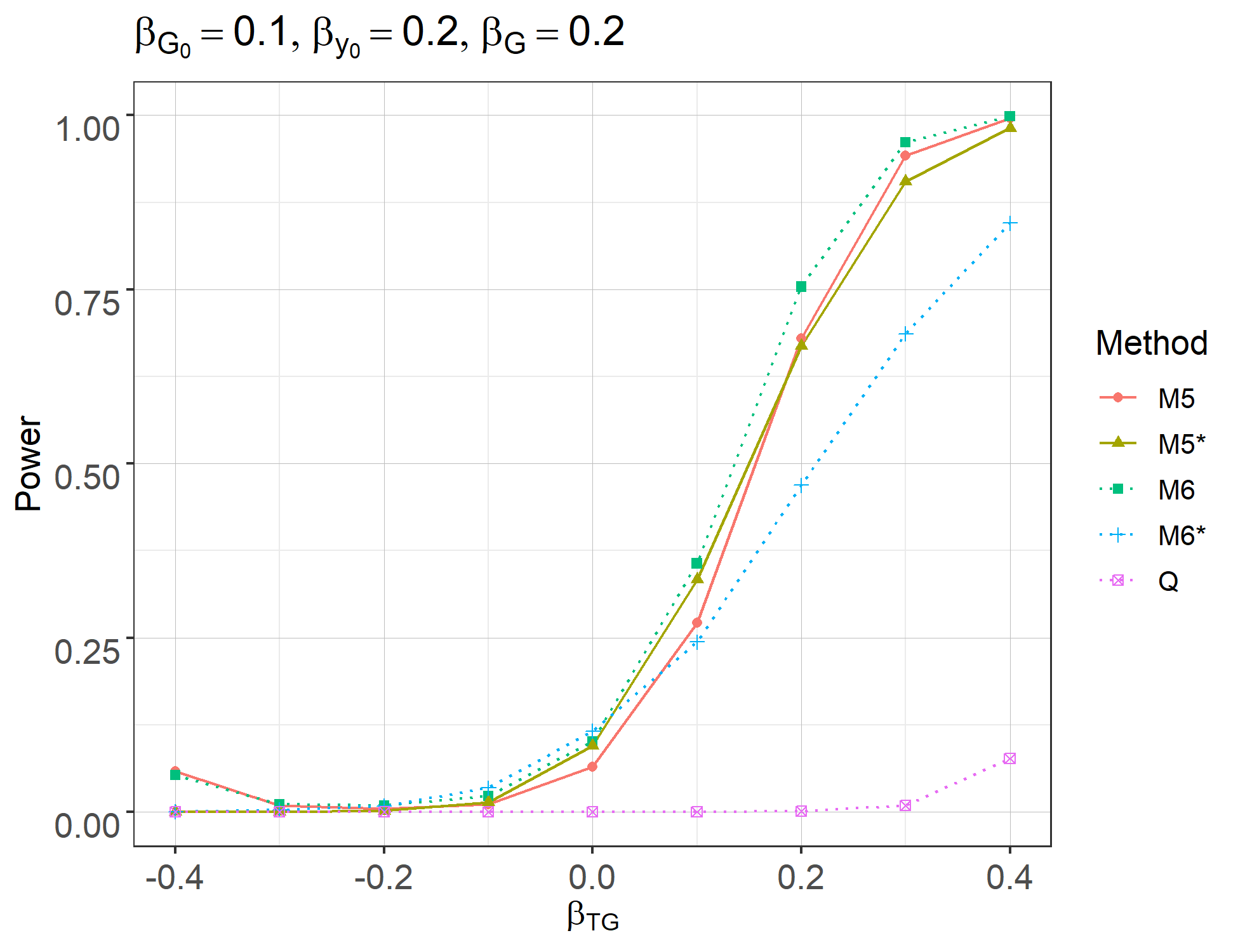

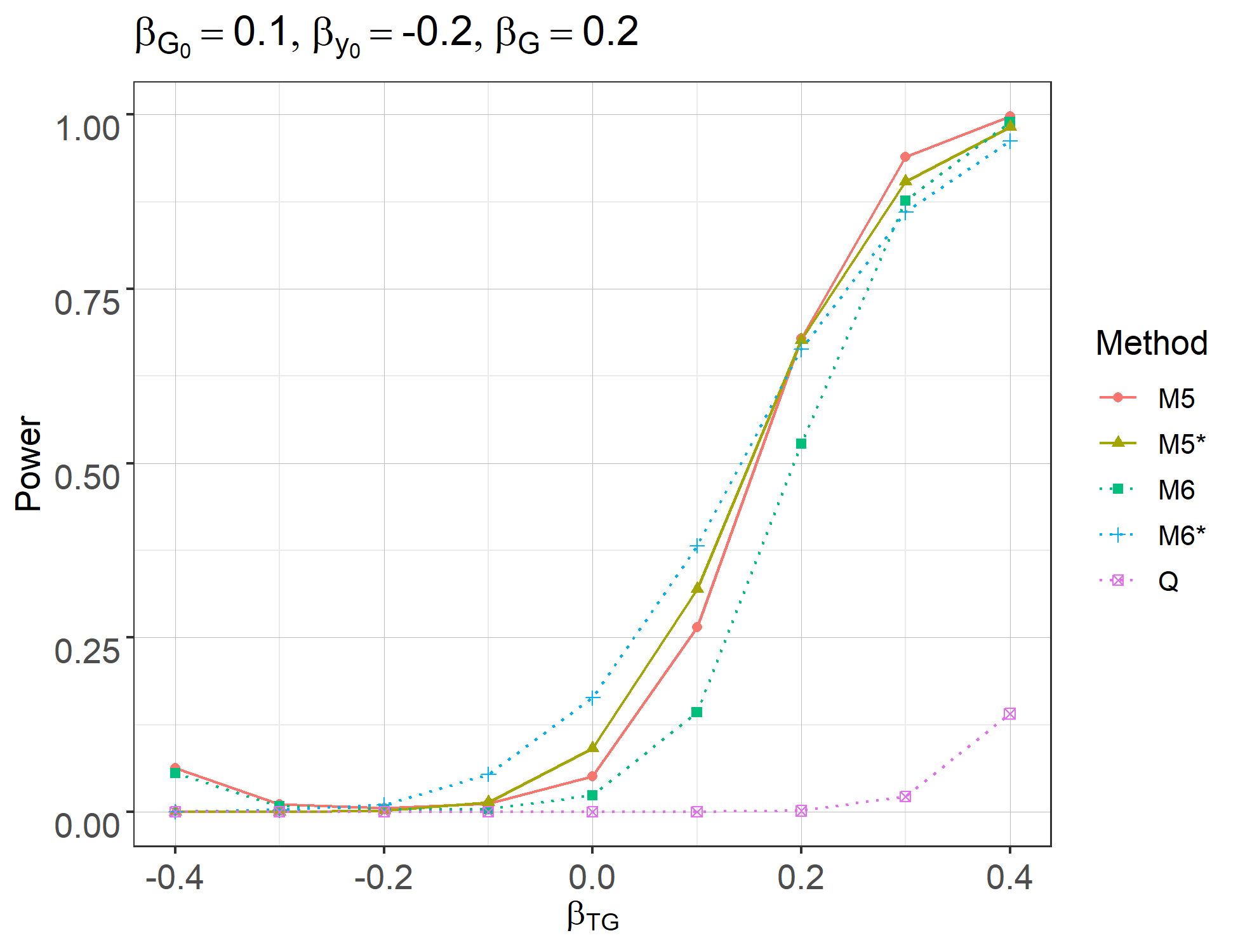

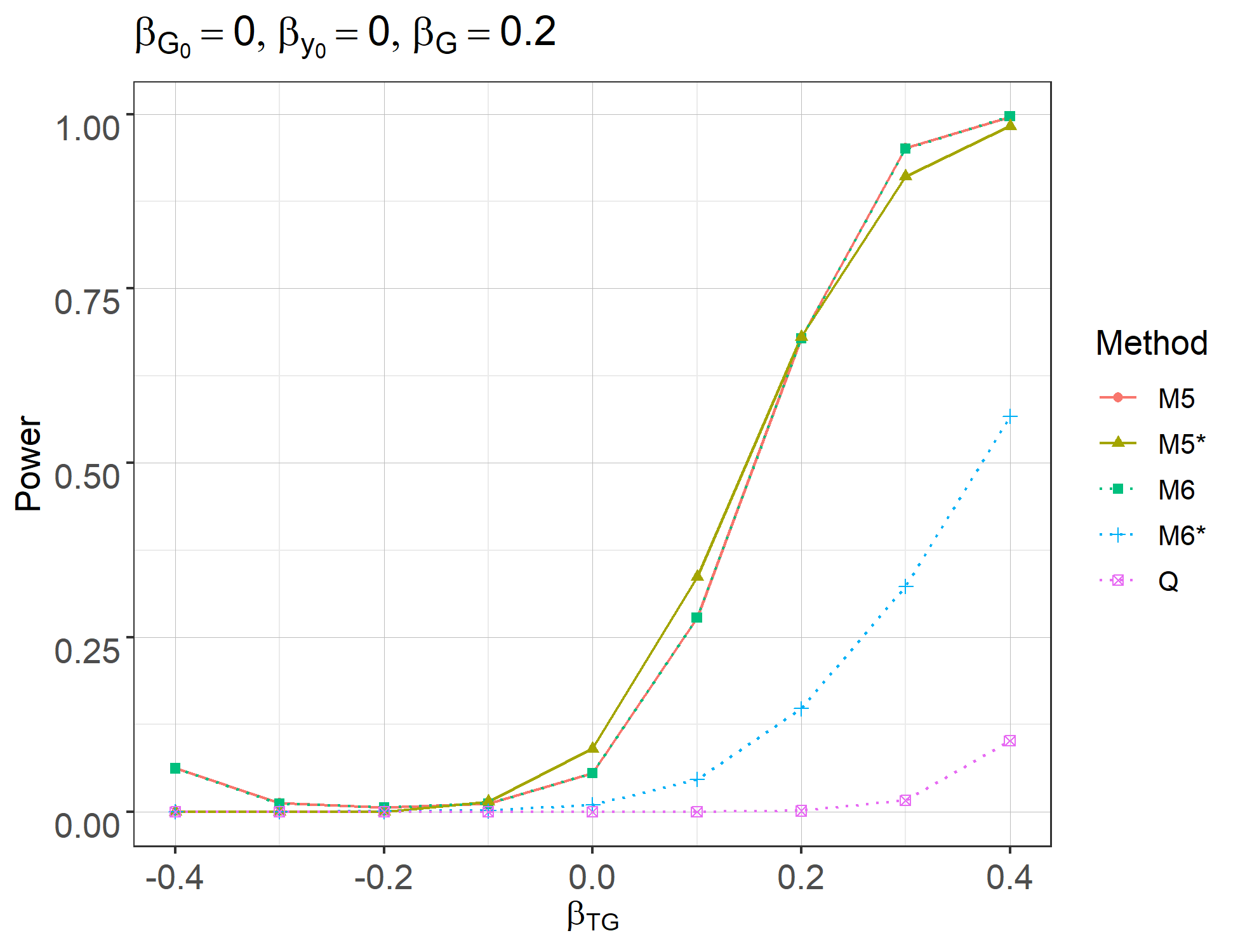


**Supplemental Figure 9**. Power comparison between models in the sensitivity analysis (M5*, M6* and Q test) with genotype effect $\beta_{G}=0.2$. Left, middle, right column: $\beta_{y_{0}}=0.2, 0, -0.2$, respectively. First, second, third row: $\beta_{G_{0}}=0.1, 0, -0.1$, respectively. $\alpha={10}^{-6}$, which is consistent with the type I error simulation. M5*-Q are defined in the Methods section.


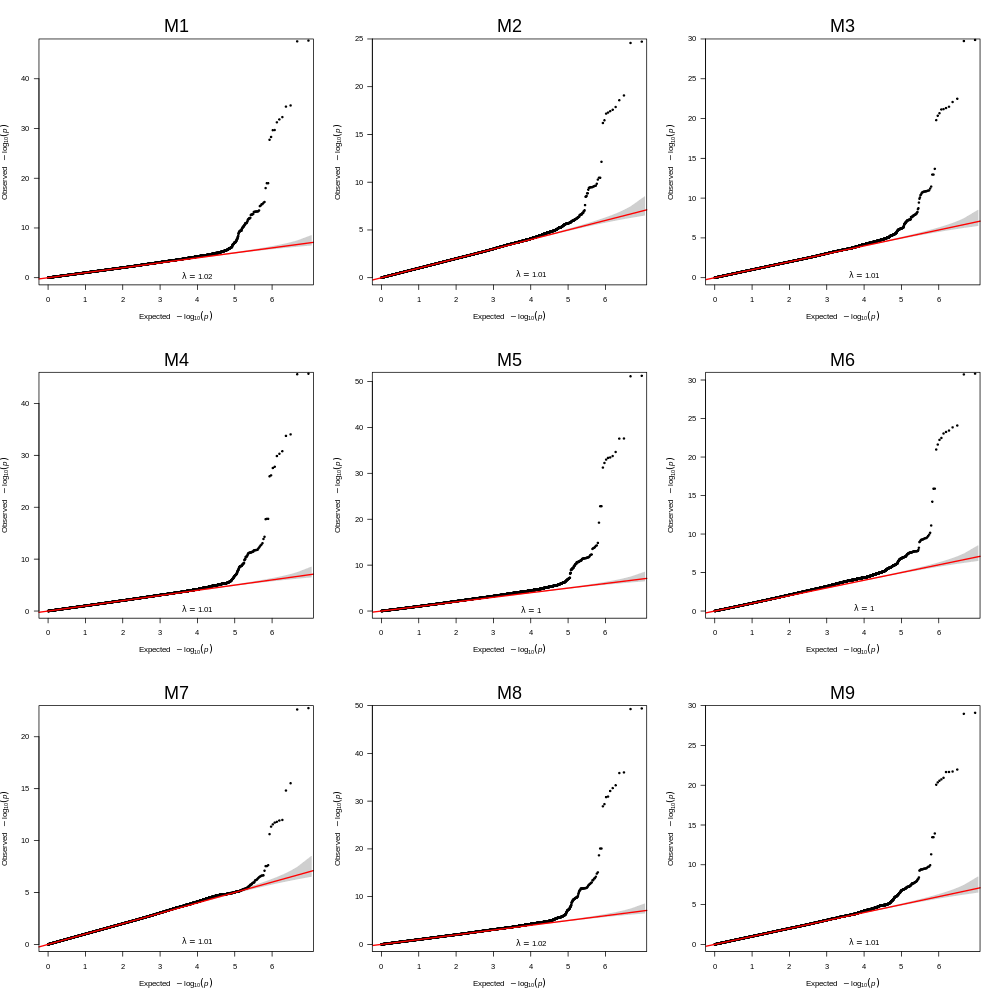


**Supplemental Figure 10**. QQ plot from GWAS analyses of change from baseline of LDL-C from IMPROVE-IT PGx study using Model 1 (M1) to Model 9 (M9). M1 uses log-fold-CFB as phenotype, adjusts for baseline LDL-C and uses a 2-step approach in the regression, in which residuals are inverse normally transformed. M2 uses CFB-ratio as phenotype, does not adjust for baseline LDL-C and uses a 2-step approach in the regression. M3 uses log-fold-CFB as phenotype, does not adjust for baseline LDL-C and uses a 2-step approach in the regression. M4 uses CFB-ratio as phenotype, adjusts for baseline LDL-C and uses 2-step approach in the regression. M5 uses log-fold-CFB as phenotype, adjusts for baseline LDL-C and uses a 2-df test in the regression. M6 uses log-fold-CFB as phenotype, does not adjust for baseline LDL-C and uses a 2-df test in the regression. M7 uses log-baseline as phenotype for baseline association test. M8 is the same as M1 except that it uses a 1-step approach in the regression instead. M9 is the same as M3 except that it uses a 1-step approach in the regression instead.


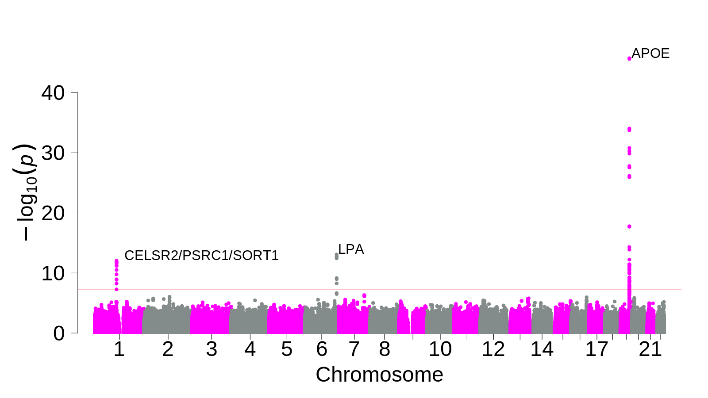

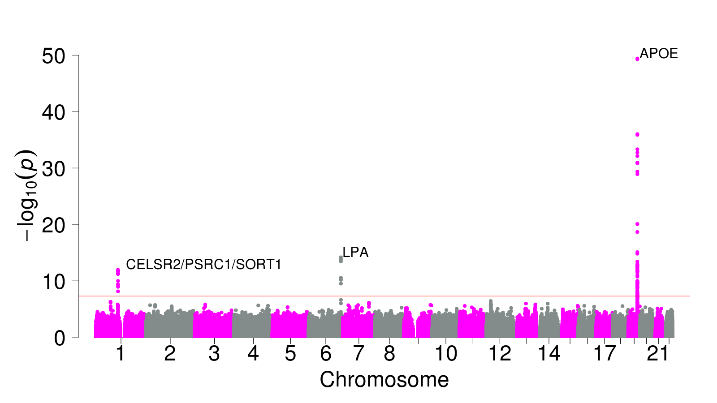


M9

M8

M2

M4


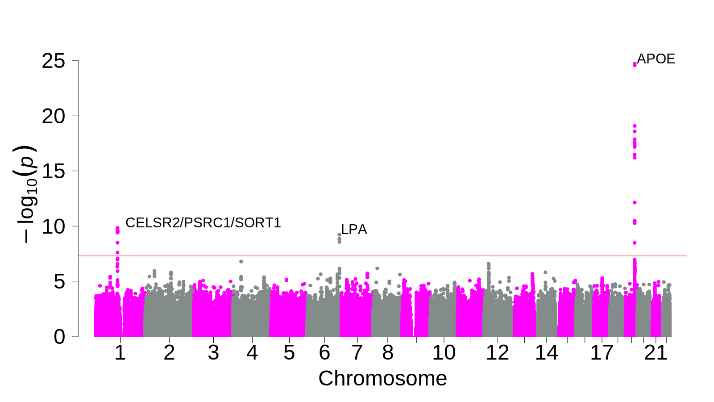

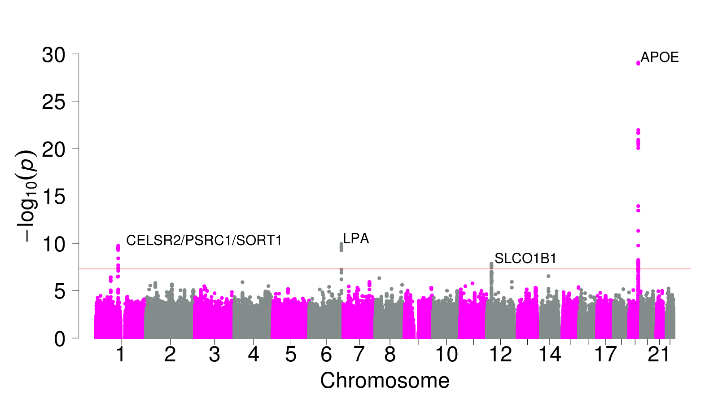


**Supplemental Figure 11**. Manhattan plots for four genome-wide association studies (GWAS) of drug-induced low-density lipoprotein cholesterol (LDL-C) response from IMPROVE-IT PGx study using Model 4 (M4), Model 2 (M2), Model 8 (M8) and Model 9 (M9). M4 used CFB ratio as phenotype, adjusted for baseline LDL-C and used a 2-step approach in the regression, in which residuals were obtained by regressing out the covariates and then inverse normally transformed. M2 was the same as M4 except that it did not adjust for baseline LDL-C in the model. Both M4 and M2 yielded the same three significant loci. M8 used log-fold-CFB as the phenotype, adjusted for baseline LDL-C and used a 1-step approach in the regression. M9 was the same as M8 except that it did not adjust for baseline LDL-C in the model. M8 yielded three significant loci while M9 yielded one more significant locus (SLCO1B1) on chromosome 12. The horizontal red line represents the whole-genome significant p-value threshold 5e-08. All tests were two-sided.


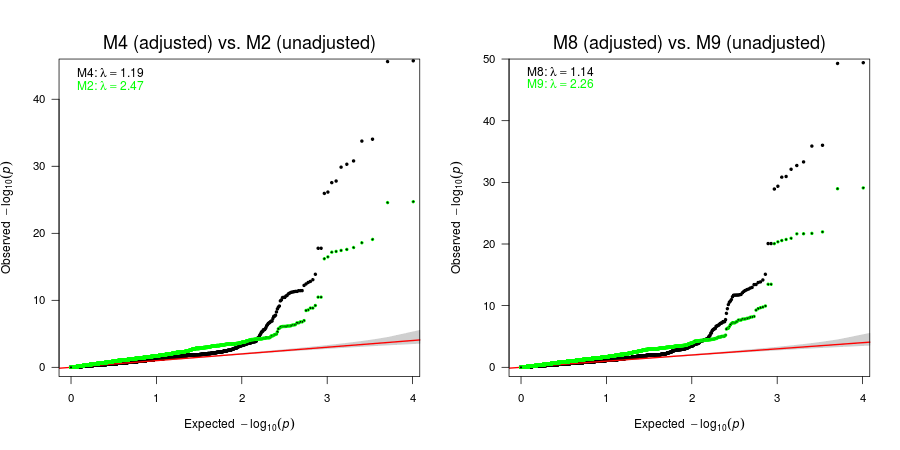


**Supplemental Figure 12**. QQ plots of the p-values between two sets of the baseline-adjusted models vs. the baseline-unadjusted models from the four GWAS analyses based on four models with CFB ratio endpoint (M4 and M2) and log-fold-CFB endpoint (M8 and M9). The variants were first filtered based on the baseline association p-value < 1e-03 from M7 and 10,187 SNPs were kept for both plots. These variants showed clear mediator effect $\beta_{G_{0}}*\beta_{y_{0}}\neq0$. The red line was the diagonal line and the 95% confidence interval polygon in each QQ plot was based on the P-values from the baseline adjusted model (M4 in the left plot and M8 in the right plot).


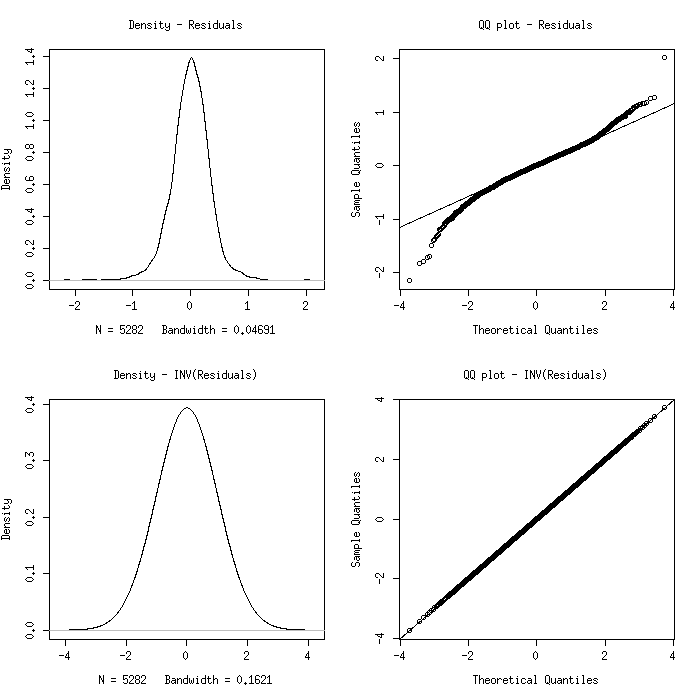


**Supplemental Figure 13**. Density and QQ plots of the residuals and inverse normal transformed residuals from the analysis of the log-fold-CFB of LDL-C from IMPROVE-IT PGx study using Model 1 (M1). M1 adjusts for baseline LDL-C and uses a 2-step approach in the regression, in which residuals are inverse normally transformed.


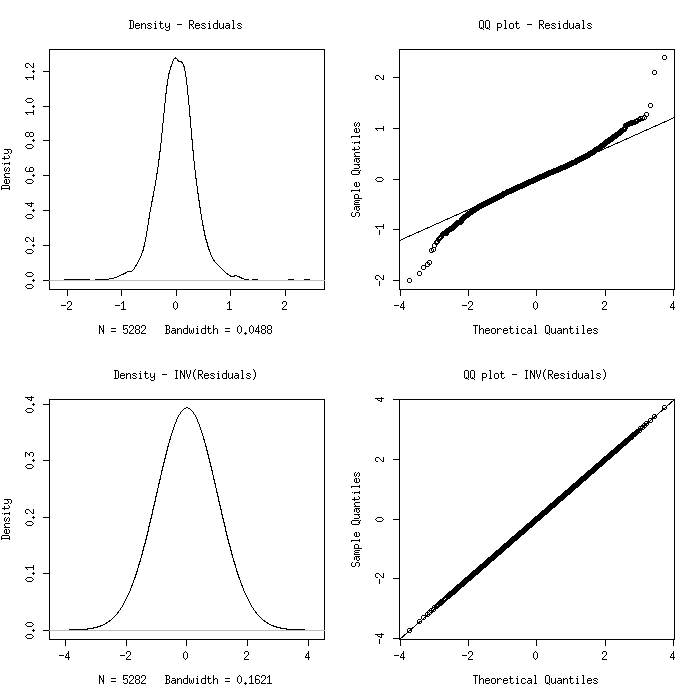


**Supplemental Figure 14**. Density and QQ plots of the residuals and inverse normal transformed residuals from the analysis of the log-fold-CFB of LDL-C from IMPROVE-IT PGx study using Model 3 (M3). M3 does not adjust for baseline LDL-C and uses a 2-step approach in the regression, in which residuals are inverse normally transformed.

**Supplemental Table 1**. All five lead variants the genome-wide significant loci for baseline-adjusted and baseline-unadjusted difference of natural log-transformed or percent change from baseline of Simvastatin & Ezetimibe/Simvastatin on low-density lipoprotein cholesterol levels using 1df test and 2df test respectively and their baseline associations in European population (N = 5,661). The association results from M4, M2, M8, M9 and M7 were included.

| **Gene** | **SNP** | **CHR** | **BP** | **MA** | **MAF** | **Model^a^** | $\boldsymbol{\beta}_{\boldsymbol{y}_{\boldsymbol{0}}}$**^b^** | $\boldsymbol{\beta}_{\boldsymbol{G}}$ **or** $\boldsymbol{\beta}_{\boldsymbol{G}_{\boldsymbol{0}}}$**^c^** | **P_G** |
| --- | --- | --- | --- | --- | --- | --- | --- | --- | --- |
| *CELSR2*/  *PSRC1*/  *SORT1* | rs599839 | 1 | 109822166 | G | 0.240 | M4 (BJ-1dfT-2SR) | -0.004 | -0.162 | 9.22E-13 |
|  |  |  |  |  |  | M2 (BuJ-1dfT-2S) |  | -0.145 | 1.46E-10 |
|  |  |  |  |  |  | M8 (BJ-1dfT-1SR) | -0.494 | -0.052 | 1.12E-12 |
|  |  |  |  |  |  | M9 (BuJ-1dfT-1SR) |  | -0.049 | 1.74E-10 |
|  |  |  |  |  |  | M7 (Baseline only) |  | -0.008 | 6.58E-02^d^ |
| *STAG1*/  *SLC35G2*/  *NCK1* | rs79929954 | 3 | 136623748 | G | 0.015 | M4 (BJ-1dfT-2SR) | -0.004 | 0.158 | 5.09E-02^d^ |
|  |  |  |  |  |  | M2 (BuJ-1dfT-2SR) |  | 0.162 | 4.46E-02^d^ |
|  |  |  |  |  |  | M8 (BJ-1dfT-1SR) | -0.490 | 0.079 | 2.68E-03^d^ |
|  |  |  |  |  |  | M9 (BuJ-1dfT-1SR) |  | 0.080 | 3.23E-03^d^ |
|  |  |  |  |  |  | M7 (Baseline only) |  | -0.002 | 8.92E-01^d^ |
| *LPA* | rs10455872 | 6 | 161010118 | G | 0.080 | M4 (BJ-1dfT-2SR) | -0.004 | 0.265 | 8.42E-14 |
|  |  |  |  |  |  | M2 (BuJ-1dfT-2SR) |  | 0.220 | 6.06E-10 |
|  |  |  |  |  |  | M8 (BJ-1dfT-1SR) | -0.499 | 0.090 | 6.82E-15 |
|  |  |  |  |  |  | M9 (BuJ-1dfT-1SR) |  | 0.078 | 1.13E-10 |
|  |  |  |  |  |  | M7 (Baseline only) |  | 0.024 | 3.68E-04^d^ |
| *SLCO1B1* | rs4149056 | 12 | 21331549 | C | 0.162 | M4 (BJ-1dfT-2SR) | -0.004 | 0.119 | 4.87E-06^d^ |
|  |  |  |  |  |  | M2 (BuJ-1dfT-2SR) |  | 0.134 | 2.47E-07^d^ |
|  |  |  |  |  |  | M8 (BJ-1dfT-1SR) | -0.486 | 0.043 | 3.76E-07^d^ |
|  |  |  |  |  |  | M9 (BuJ-1dfT-1SR) |  | 0.050 | 1.33E-08 |
|  |  |  |  |  |  | M7 (Baseline only) |  | -0.013 | 9.64E-03^d^ |
| *APOE* | rs1065853 | 19 | 45413233 | T | 0.089 | M4 (BJ-1dfT-2SR) | -0.004 | -0.481 | 1.84E-46 |
|  |  |  |  |  |  | M2 (BuJ-1dfT-2SR) |  | -0.352 | 1.92E-25 |
|  |  |  |  |  |  | M8 (BJ-1dfT-1SR) | -0.536 | -0.164 | 3.91E-50 |
|  |  |  |  |  |  | M9 (BuJ-1dfT-1SR) |  | -0.130 | 7.99E-30 |
|  |  |  |  |  |  | M7 (Baseline only) |  | -0.064 | 2.35E-23 |

*SNP* **single nucleotide polymorphism**, *CHR* **chromosome**, *BP* **base pair**, *MA* **minor allele**, *MAF* **minor allele frequency**, $\beta_{y_{0}}$**effect size of baseline variable**, *β_G_* **effect size of G (genotype) on CFB (from M4, M2, M8 and M9)**, $\beta_{G_{0}}$ **effect size of G (genotype) on baseline (from M7)**, *P_G* **P-value of G (genotype)**.

^a^BJ: Baseline-adjusted; BuJ: Baseline-unadjusted; 1dfT: 1 degree of freedom test; 2dfT: 2 degree of freedom test or joint test of genotype and genotype*treatment interaction; 1-SR: 1-step regression; 2-SR: 2-step regression. In M4 and M2, percentage of change from baseline of Simvastatin & Ezetimibe/Simvastatin on low-density lipoprotein cholesterol levels were used for analysis. M8 and M9, difference of natural log-transformed Simvastatin & Ezetimibe/Simvastatin on low-density lipoprotein cholesterol levels were used for analysis.

^b^Effects calculated for the baseline LDL-C (from M4) or the nature log transformed baseline LDL-C (from M8).

^c^Effects calculated with respect to the minor allele. A negative value indicates more intense drug (Simvastatin & Ezetimibe/Simvastatin) LDL-C lowering.

^d^Not reaching genome-wide significance (P < 5E-08). For 2df test methods, p-values from the 2df test (P_2df) were used.

**Supplemental Table 2**. Association results for the two loci at *LDLR* and *APOB* in IMPROVE-IT GWAS analysis which just met genome-wide significance thresholds in Oni-Orisan *et al.* (2020) in baseline adjusted analyses but were not detected in the IMPROVE-IT analysis. The association results from M1 – M9 were included.

| **Gene** | **SNP** | **CHR** | **BP** | **MA** | **MAF** | **Model^a^** | $\boldsymbol{\beta}_{\boldsymbol{G}}$ **or** $\boldsymbol{\beta}_{\boldsymbol{G}_{\boldsymbol{0}}}$**^b^** | **P_G** | $\boldsymbol{\beta}_{\boldsymbol{GT}}$**^c^** | **P_GT^c^** | **P_2df^c^** |
| --- | --- | --- | --- | --- | --- | --- | --- | --- | --- | --- | --- |
| *APOB* | rs1713222 | 2 | 21271323 | A | 0.158 | M1 (BJ-1dfT-2SR) | -0.034 | 1.95E-01 |  |  |  |
|  |  |  |  |  |  | M2 (BuJ-1dfT-2S) | -0.014 | 6.09E-01 |  |  |  |
|  |  |  |  |  |  | M3 (BuJ-1dfT-2SR) | -0.017 | 5.14E-01 |  |  |  |
|  |  |  |  |  |  | M4 (BJ-1dfT-2SR) | -0.037 | 1.67E-01 |  |  |  |
|  |  |  |  |  |  | M5 (BJ-2dfT) | -0.011 | 2.06E-01 | 0.002 | 8.86E-01 | 4.45E-01 |
|  |  |  |  |  |  | M6 (BuJ-2dfT) | -0.006 | 5.32E-01 | 0.001 | 9.61E-01 | 8.22E-01 |
|  |  |  |  |  |  | M7 (Baseline only) | -0.010 | 4.28E-02 |  |  |  |
|  |  |  |  |  |  | M8 (BJ-1dfT-1SR) | -0.011 | 2.06E-01 |  |  |  |
|  |  |  |  |  |  | M9 (BuJ-1dfT-1SR) | -0.006 | 5.32E-01 |  |  |  |
| *SMARCA4*/  *LDLR* | rs67337506 | 19 | 11207982 | C | 0.214 | M1 (BJ-1dfT-2SR) | -0.048 | 4.16E-02 |  |  |  |
|  |  |  |  |  |  | M2 (BuJ-1dfT-2S) | -0.041 | 8.59E-02 |  |  |  |
|  |  |  |  |  |  | M3 (BuJ-1dfT-2SR) | -0.037 | 1.13E-01 |  |  |  |
|  |  |  |  |  |  | M4 (BJ-1dfT-2SR) | -0.051 | 3.14E-02 |  |  |  |
|  |  |  |  |  |  | M5 (BJ-2dfT) | -0.016 | 3.49E-02 | 0.002 | 8.88E-01 | 1.07E-01 |
|  |  |  |  |  |  | M6 (BuJ-2dfT) | -0.013 | 9.69E-02 | 0.003 | 8.57E-01 | 2.48E-01 |
|  |  |  |  |  |  | M7 (Baseline only) | -0.005 | 2.24E-01 |  |  |  |
|  |  |  |  |  |  | M8 (BJ-1dfT-1SR) | -0.016 | 3.49E-02 |  |  |  |
|  |  |  |  |  |  | M9 (BuJ-1dfT-1SR) | -0.013 | 9.69E-02 |  |  |  |

*SNP* **single nucleotide polymorphism**, *CHR* **chromosome**, *BP* **base pair**, *MA* **minor allele**, *MAF* **minor allele frequency**, *β_G_* **effect size of G (genotype) on CFB (from M1-M6 and M8-M9)**, $\beta_{G_{0}}$ **effect size of G (genotype) on baseline (from M7)**, *P_G* **P-value of G (genotype)**, *β_GT_* **effect size of G*T (genotype by treatment interaction)**, *P_GT* **P-value of G*T (genotype by treatment interaction)**, *P_2df* **P-value of 2df test (joint test of genotype and genotype*treatment interaction)**.

^a^BJ: Baseline-adjusted; BuJ: Baseline-unadjusted; 1dfT: 1 degree of freedom test; 2dfT: 2 degree of freedom test or joint test of genotype and genotype*treatment interaction; 1SR: 1-step regression; 2SR: 2-step regression. In M1, M3, M5 and M6, difference of natural log-transformed Simvastatin & Ezetimibe/Simvastatin on low-density lipoprotein cholesterol levels were used for analysis.

^b^Effects calculated with respect to the minor allele. A negative value indicates more intense drug (Simvastatin & Ezetimibe/Simvastatin) LDL-C lowering

^c^Results were only available in the 2df test model M5 and M6, which also tests the genotype*treatment interaction and joint test of genotype and genotype*treatment interaction.
